# Synthesis and Structure–Activity Relationship of Thiourea Derivatives Against *Leishmania amazonensis*

**DOI:** 10.3390/ph17121573

**Published:** 2024-11-23

**Authors:** Gil Mendes Viana, Edézio Ferreira da Cunha-Junior, Paloma Wetler Meireles Carreiros Assumpção, Marianne Grilo Rezende, Yago Sousa dos Santos Emiliano, Laiza Maria da Silva Soares, Gabriel Rodrigues Coutinho Pereira, Carlos Rangel Rodrigues, Lucio Mendes Cabral, Eduardo Caio Torres-Santos

**Affiliations:** 1 Laboratório de Tecnologia Industrial Farmacêutica, Faculdade de Farmácia, Universidade Federal do Rio de Janeiro, Rio de Janeiro 21941-590, Brazil; gmviana@gmail.com (G.M.V.); palomawetler@yahoo.com.br (P.W.M.C.A.); mariannegrezende@gmail.com (M.G.R.); 2 Laboratório de Imunoparasitologia, Unidade Integrada de Pesquisa em Produtos Bioativos e Biociências, Centro Multidisciplinar UFRJ-Macaé, Universidade Federal do Rio de Janeiro, Macaé 27970-000, Brazil; edezio@macae.ufrj.br; 3 Laboratório de Bioquímica de Tripanosomatídeos, Instituto Oswaldo Cruz, Fundação Oswaldo Cruz, Rio de Janeiro 21040-900, Brazil; yago.emiliano@ioc.fiocruz.br (Y.S.d.S.E.); laizamss@hotmail.com (L.M.d.S.S.); 4 Laboratório ModMolQSAR, Faculdade de Farmácia, Universidade Federal do Rio de Janeiro, Rio de Janeiro 21941-590, Brazil; gabrielrodriguescp@gmail.com (G.R.C.P.); rangelfarmacia@gmail.com (C.R.R.)

**Keywords:** thiourea derivatives, leishmaniasis, *Leishmania amazonensis*, medicinal chemistry, SARs, drug discovery

## Abstract

**Background:** Leishmaniasis, caused by *Leishmania* protozoa and transmitted by vectors, presents varied clinical manifestations based on parasite species and host immunity. The lack of effective vaccines or treatments has prompted research into new therapies, including thiourea derivatives, which have demonstrated antiprotozoal activities. **Methods:** We synthesized two series of *N*,*N*′-disubstituted thiourea derivatives through the reaction of isothiocyanates with amines. These compounds were evaluated in vitro against promastigote and amastigote forms of *L. amazonensis*, alongside cytotoxicity assessments on macrophages. In silico studies were conducted to analyze structure–activity relationships (SARs) and drug-likeness. **Results:** A total of fifty thiourea derivatives were synthesized and tested. Compound **3e** from the first generation exhibited significant anti-leishmanial activity with an IC_50_ of 4.9 ± 1.2 µM and over 80-fold selectivity compared to that of miltefosine (IC_50_ = 7.5 ± 1.2 µM). The introduction of a piperazine ring in the second-generation thioureas enhanced potency and selectivity, with compound **5i** achieving an IC_50_ of 1.8 ± 0.5 µM and a selectivity index of approximately 70. Pharmacokinetic predictions indicated favorable profiles for the active compounds. **Conclusions:** SAR and ADMET analyses identified compound **5i** as the most promising candidate for further preclinical evaluation, suggesting that piperazine thiourea derivatives represent a novel class of anti-leishmanial agents.

## 1. Introduction

Leishmaniasis is a group of diseases classified by the World Health Organization (WHO) as Neglected Tropical Diseases. These diseases are transmitted by vectors and caused by protozoa of the genus *Leishmania* [1]. The WHO reported that in 2020, leishmaniasis was present in 98 countries or territories, and it posed a severe public health issue in four eco-epidemiological regions across the globe: the Americas, East Africa, North Africa, and West and Southeast Asia. That year, there were reports of 208,357 cases of Cutaneous Leishmaniasis (CL) and 12,838 cases of Visceral Leishmaniasis (VL). It is important to note that CL is the most common form, typically characterized by the development of skin ulcers. At the same time, VL is the most severe form and can be fatal if not treated [2]. Leishmaniasis ranked as the third most common parasitic disease in 2015, after schistosomiasis and malaria, based on measures of morbidity and disability-adjusted life years (DALYs) [3]. Clinical manifestations of leishmaniasis vary depending on the parasite’s tropism and the host’s immune status. Notably, of the 31 species that infect mammalian cells, 20 are described as pathogenic for humans [4].

The available treatment for leishmaniasis includes the following drugs: sodium stibogluconate (Pentostam^®^) and meglumine antimoniate (Glucantime^®^), which are derivatives of pentavalent antimony, amphotericin B and its lipid formulations, pentamidine, miltefosine, and paromomycin. Antimonial therapy lasts up to 28 days of parenteral administration, and its efficacy varies against different forms of leishmaniasis. The use of pentamidine is limited due to toxicity issues [5]. Amphotericin B has a broad spectrum of effectiveness but is associated with toxicity and requires slow parenteral infusion over four hours. Liposomal formulations of amphotericin B, such as AmBisome^®^, have reduced toxicity, have a longer plasma half-life, and have been approved for clinical use but are expensive [6,7]. A significant advancement in leishmaniasis therapy has been the introduction of miltefosine, a drug initially developed as an anticancer agent. Existing drugs generally have disadvantages in terms of therapeutic approaches, such as the route of administration, the need for trained professionals for administration in most cases, toxicity, and high cost, and considering the number of pathogenic species concerning therapeutic options, this arsenal still needs to be improved [5,8].

**Figure 1:**
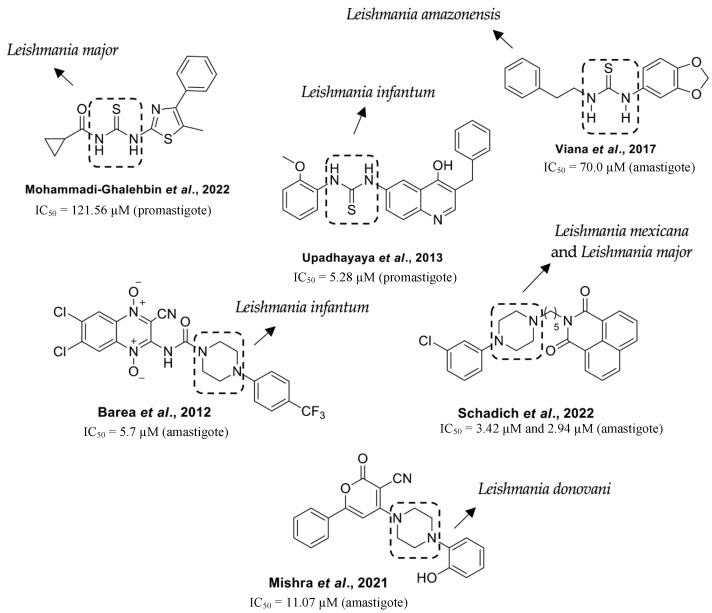
Thiourea and piperazine derivatives as anti-leishmanial agents [16,17,18,19,21,23].

The lack of a vaccine or effective chemotherapy has stimulated many studies involving the research and development of new drugs with potential leishmanicidal activity [9]. Thiourea derivatives are widely described in the literature and have proved helpful in drug research. Several studies have demonstrated the importance of these class of molecules in medicinal chemistry since they exhibited a wide range of biological activities such as anticancer [10], antimicrobial [11], antimycobacterial [12], antiviral [13], and anti-inflammatory ones [14]. In addition to these biological activities, thiourea derivatives also demonstrated antiprotozoal activities. Some studies have already shown their activities against *Plasmodium falciparum* [15], *Trypanosoma cruzi* [16], *Leishmania major* [17], and *L. infantum* [16]. In a previous work carried out by our research group, different *N*,*N*′-disubstituted thioureas were evaluated against promastigote and amastigote forms of *L. amazonensis*, with low cytotoxicity in murine macrophages, indicating that these derivatives are promising for the treatment of leishmaniasis [18]. Some examples of these thiourea derivatives are given in Figure 1. The incorporation of the piperazine group has been an attractive alternative since this heterocyclic ring is present in molecules with proven in vitro leishmanicidal activity for *L. infantum* [19,20], *L. major*, and *L. mexicana* [21,22] and in vivo for *L. donovani* [23]. The main objective of this work was to discover new compounds that demonstrate efficacy against *L. amazonensis*, ensuring safety and selectivity. For this, two generations of substituted thiourea derivatives were planned and synthesized, totaling fifty compounds. We evaluated its activity profile on *L. amazonensis* by carrying out a study on the structure–activity relationship, in which we verified that the optimization of the first generation (G1) of thioureas with the piperazine moiety forming a piperazine thioureas (second generation, G2).

## 2. Results

### 2.1. Chemistry

**Scheme 1:**
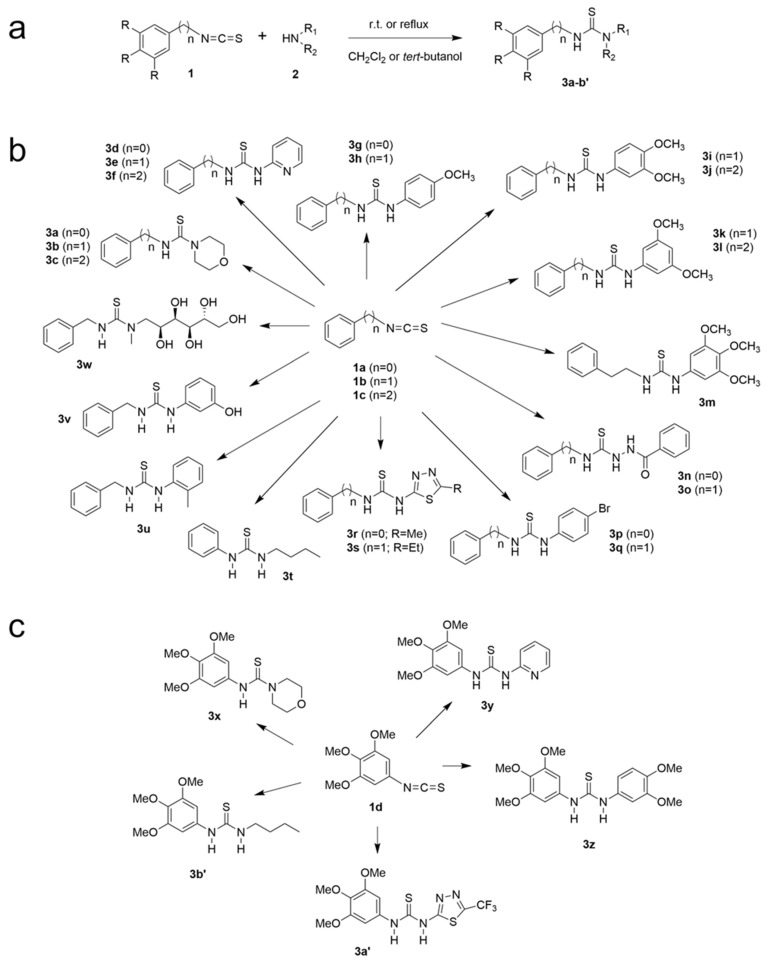
Synthesis of first-generation series of thiourea derivatives: (**a**) general synthesis scheme; (**b**) thioureas **3a**–**3w** obtained from phenyl isothiocyanate (**1a**), benzyl isothiocyanate (**1b**), and phenethyl isothiocyanate (**1c**); (**c**) thioureas **3x**–**3b′** obtained from 3,4,5-trimethoxyphenyl isothiocyanate (**1d**).

The first-generation series of thiourea compounds (**3a**–**3b′**) were obtained as summarized in Scheme 1. These thiourea derivatives were synthesized through the reaction of phenyl isothiocyanate (**1a**), benzyl isothiocyanate (**1b**), phenethyl isothiocyanate (**1c**), or 3,4,5-trimethoxyphenyl isothiocyanate (**1d**) with the respective amine **2**, in a dichloromethane or tert-butanol solution (Table 1). The reaction of isothiocyanates and amines was considered as a simple reaction, but it can exhibit high yields and it is possible to obtain a great structural diversity of substituted thioureas [18,24]. All aliphatic primary and secondary amines afforded the corresponding thioureas at room temperature in this protocol. In contrast, the reactions involving aromatic amines required a reflux period due to the lower reactivity of the amines, with *tert*-butanol as a non-volatile and non-nucleophilic solvent.

**Scheme 2:**
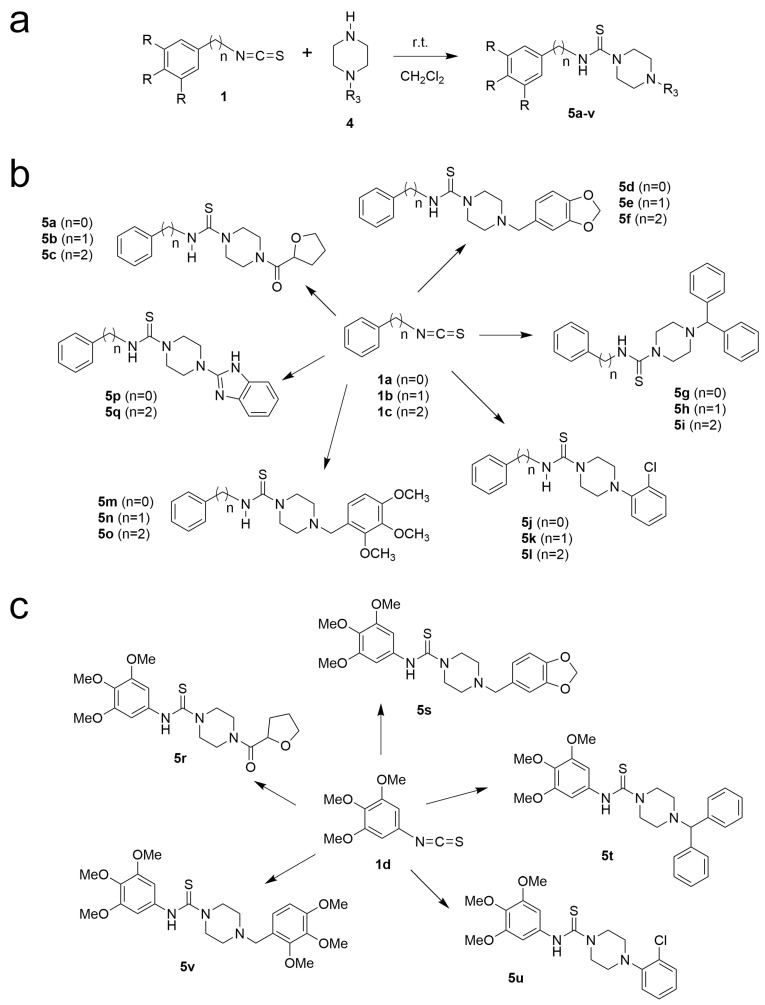
Synthesis of second-generation series of thiourea derivatives: (**a**) general synthesis scheme; (**b**) thioureas **5a**–**5q** obtained from phenyl isothiocyanate (**1a**), benzyl isothiocyanate (**1b**), and phenethyl isothiocyanate (**1c**); (**c**) thioureas **5r**–**5v** obtained from 3,4,5-trimethoxyphenyl isothiocyanate (**1d**).

In addition, the piperazine thioureas (**5a**–**5v**) were synthesized through the reaction of isothiocyanate **1a**–**d** and *N*-monosubstituted piperazine **4**, in a dichloromethane solution at room temperature since these amines have enough nucleophilicity for a short-time reaction (1 h). The synthesis of these thiourea derivatives is outlined in Scheme 2, and the corresponding details are provided in Table 2.

All compounds were obtained with a good to excellent yield (greater than 70%), demonstrating the good viability of the synthetic strategy employed, and they were completely characterized by ^1^H-NMR, ^13^C-NMR, FT-IR, and HR-MS. NMR spectra for thiourea derivatives are available in the Supplementary Materials (Figures S1–S32). The ^13^C-NMR spectra exhibit a chemical shift corresponding to the thiocarbonyl (C=S) of the thiourea moiety within the range of 178 to 184 ppm, providing confirmation of product formation. Additionally, the formation of the compounds is evident in the ^1^H-NMR spectra, where characteristic signals (broad singlets) of hydrogen atoms linked to the nitrogen atoms of the thiourea moiety are observed. Typically, these signals appear between 5.50 and 6.30 ppm (NH attached to alkyl chain) and 7.30 and 8.50 ppm (NH attached to aryl substituents). In the ^1^H-NMR spectra of piperazine thioureas, the hydrogen signals of the piperazine ring are also discernible between 3.70 and 4.10 ppm and 2.40 and 3.10 ppm, usually appearing as two triplets. Furthermore, the HR-MS values of all synthesized compounds confirmed their molecular weight.

Despite its simplicity, involving only a single reaction step, this synthetic approach afforded seventeen thioureas (**3r**, **3s**, **3a′**, **3b′**, **5c**, **5f**, **5h**, **5i**, **5k**, **5l**, **5o**, **5p**, **5r**, **5s**, **5t**, **5u**, and **5v**) that have not been previously described in the literature.

### 2.2. Biological Activity

#### 2.2.1. Anti-Leishmanial Activity and Toxicity of First-Generation Thioureas (G1)

Biological activity assays on the parasite were carried out in the promastigote and intracellular amastigote forms of *L. amazonensis*, and the toxicity was evaluated in murine macrophages. The first generation of thioureas (**3a**–**3b′**) was primarily assessed in the promastigote form. Out of the 28 compounds tested in this form, 12 exhibited activities with an IC_50_ ranging from 19.0 µM to 80.0 µM (Table 3). The thioureas **3d**, **3e**, **3p**, and **3q** exhibited IC_50_ values below 50.0 µM, following the respective order of potency, although none of them showed better results than miltefosine (6.9 ± 2.4 µM). Concerning toxicity, these compounds (**3d**, **3e**, **3p**, and **3q**) did not exhibit toxicity up to the maximum tested concentration (400 µM) (Table 4). Thioureas **3d**, **3e**, and **3p** maintained activity against intracellular amastigotes with the respective IC_50_ values as follows: 10.8 ± 2.4 µM, 4.9 ± 1.2 µM, and 8.7 ± 1.4 µM. However, compound **3q** showed no activity even at the highest tested concentration (25 µM). In this first generation of thioureas, compound **3e** emerged as the most promising, with an IC_50_ below 5.0 µM and a selectivity greater than 80 times, surpassing the positive control (miltefosine; IC_50_ = 7.5 ± 1.2 µM) against the amastigote form of *L. amazonensis*.

These first-generation tests revealed that the trimethoxylated compounds in R (Scheme 1, Table 3), except for thiourea **3a′**, did not exhibit activity in the promastigote form (compounds **3x**, **3y**, **3z**, and **3b′**).

#### 2.2.2. Anti-Leishmanial Activity and Toxicity of Second-Generation Thioureas (G2)

Thiourea derivatives containing a piperazine ring (**5a**–**5v**) were also evaluated as anti-leishmanial agents in assays using promastigotes and amastigotes of *L. amazonensis*. Eleven thioureas inhibited the proliferation of the promastigote form of the parasite, with IC_50_ values ranging from 6.9 to 90.2 µM (Table 5). Despite most of these compounds having IC_50_ values higher than those of miltefosine (6.9 ± 2.4 µM), thiourea **5i** (6.9 ± 2.0 µM) demonstrated equivalent potency. Subsequently, the most potent thioureas (**5e**, **5g**, **5h**, **5i, 5k**, **5l**, **5n**, **5q**, and **5t**) were evaluated against the amastigote form in a concentration of 10 µM, with only thioureas **5h**, **5i**, **5q**, and **5t** showing results within the tested concentration range and with IC_50_ values being between 1.8 and 8.8 µM (Table 6); however, compound **5q** in the dose–response curve test presented an IC_50_ > 10µM. Toxicity in murine macrophages was also assessed, and despite piperazine thioureas generally being more cytotoxic compared to first-generation thioureas, compound **5i** stood out with a selectivity index of about 70 times. Thiourea **5i** (1.8 ± 0.5 µM) demonstrated greater potency than miltefosine (7.5 ± 1.2 µM) against the amastigote form of *L. amazonensis*.

### 2.3. Structure–Activity Relationship Study

Our objective with the structure–activity relationship studies was to determine if there was a correlation between the calculated physicochemical properties data (shown in Tables S1 and S2—Supplementary Materials) and the pharmacological activity of thioureas G1 and G2. Therefore, we collected the calculated parameters and the corresponding IC_50_ values into Table 7 and Table 8. As the two series of thiourea derivatives have structural differences arising from the amines used in the synthesis (aliphatic/aromatic amines or piperazines), correlations between their physicochemical parameters and inhibitory potencies against promastigotes of *L. amazonensis* were considered individually. Based on the limited database of thioureas assayed against amastigotes of *L. amazonensis*, it was not possible to explore any correlation of their structural features that enables rational exploration in SAR studies.

The main physicochemical properties that could influence the antipromastigote activity of these derivatives, such as lipophilicity, polarity, and isoelectric point, showed no correlation with their respective IC_50_ values. Nonetheless, as shown in Table 7, the properties that exhibited a significant and strong correlation (*p*-value < 0.05) with activity were the number of hydrogen bond acceptors (0.66), rotatable bonds (0.58), and hydrogen bond donors (0.58). Notably, this represents a positive correlation, indicating that an increase in these properties negatively impacts the compound’s potency.

Overall, the results obtained with the second-generation series of thioureas (**G2**) show a significant negative correlation (*p*-value < 0.05) between lipophilicity-related descriptors, including LogP (−0.82), Log(Koc) (−0.82), Log(BCF) (−0.82), the C Ratio (−0.75), and the number of aromatic rings (−0.75), and the IC_50_ of the compounds (Table 8). These results show a negative Pearson correlation, as IC_50_ values decrease with increasing lipophilicity, which enhances the antipromastigote potency of the compounds. As expected, the NO ratio (0.71) and Hetero Ratio (0.70), which contribute to decreasing the compound’s lipophilicity, exhibited a strong and significant positive correlation (*p*-value < 0.05) with IC_50_.

### 2.4. In Silico Pharmacokinetics and Toxicological Assessment

Unsatisfactory ADMET properties (absorption, distribution, metabolism, excretion, and toxicity) are a major cause of drug candidate failure [25]. In this scenario, accurate in silico tools for ADMET prediction, such as ADMET Predictor^TM^, could provide a rational basis for decision-making in the early phases of drug discovery [26]. ADMET Predictor^TM^ is a machine learning tool trained on subsets of the World Drug Index (WDI), a comprehensive database of commercial drugs. The pharmacokinetics and toxicological assessment of ADMET Predictor^TM^ are based on a risk system in which the score values achieved by the target compound are compared with those of the WDI empirical distribution (Simulations Plus, Inc., Lancaster, CA, USA). Compounds that exceed the established safety or efficacy limits for commercial drugs signal potential concerns. As shown in Table S3 (Supplementary Materials), the active thioureas synthesized by our group generally presented drug-like characteristics with low toxicity. Nonetheless, compounds **5g**, **5h**, **5i**, **5k**, and **5l** may have metabolic problems due to elevated intrinsic clearance (CYP risk > 2). Despite that, compound **5i**, which is the most active thiourea in the series, i.e., IC_50_ = 1.8 ± 0.5 µM (Table 6), was predicted to have low toxicity and optimal absorption, distribution, and excretion considering oral administration.

## 3. Discussion

**Figure 2:**
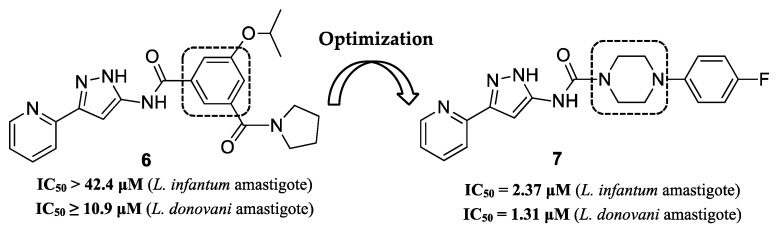
Hit optimization of urea derivatives containing piperazine ring designed by Mowbray et al. (2015) [28].

In the first-generation series of thiourea derivatives (**3a**–**3b′**), any one of them showed better activity than miltefosine against the promastigote form of *L. amazonensis*. Despite these results, thiourea **3e** stood out in the in vitro assay against the amastigote form (IC_50_ = 4.9 ± 1.2 µM), with better activity than that of the positive control (miltefosine IC_50_ = 7.5 ± 1.2 µM), showing the potential of this class of compounds against this protozoan. The success of compound **3e** (IC_50_ < 10µM) against intracellular amastigotes is particularly relevant since the amastigote stage is responsible for the clinical symptoms in mammals and represents a critical therapeutic target, being one of the criteria pointed out by Katsuno et al. (2015) for choosing a hit for leishmaniasis [27]. In our SAR studies, findings suggest that, among the tested thioureas, the number of spacer carbons plays a crucial role in maintaining activity against both promastigotes and amastigotes of *L. amazonensis*. The lipophilicity of the substituents derived from isothiocyanate (Scheme 1) was a determinant for the activity in the first generation of thiourea derivatives (**G1**). The results indicate that spacer length (n) in these series influences the inhibition of promastigote growth. When comparing thioureas **3d** and **3e**, **3p** and **3q**, **3n** and **3o**, it is possible to observe that as the spacer size (n) increases, the biological activity decreases. No thiourea derived from phenethyl isothiocyanate showed activity against the promastigote form. Thus, it can be said that the increase in the lipophilicity (LogP) of the substituent from isothiocyanate results in a decrease in antipromastigote activity. In order to obtain a new series of thioureas, with greater potency and selectivity against the parasite, it was decided to investigate the incorporation of the piperazine ring in the structure of these derivatives, as already described by Mowbray et al. [28]. The authors identified a series of amino-pyrazole ureas (**7**) with potent in vitro anti-leishmanial activity, developed from a single high-throughput screening (HTS) hit (**6**) (Figure 2). In this way, it was possible to design the second generation of thiourea derivatives (**5a**–**5v**), obtained from different substituted piperazines.

**Figure 3:**
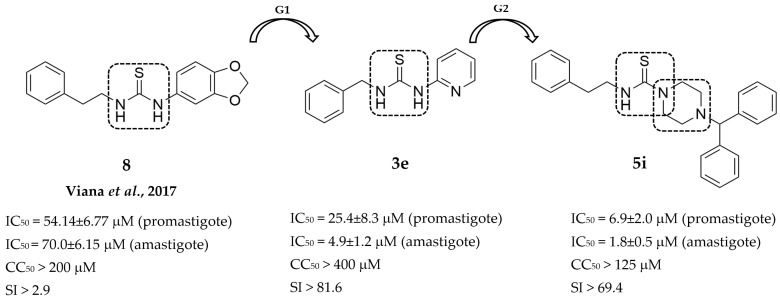
Hit optimization of thiourea derivatives.

In our studies, it was possible to observe that the incorporation of the piperazine ring into the thiourea function generally increased the potency of these derivatives, both against the promastigote and amastigote forms (Figure 3), also enhancing their respective selectivity indices.

**Figure 4:**
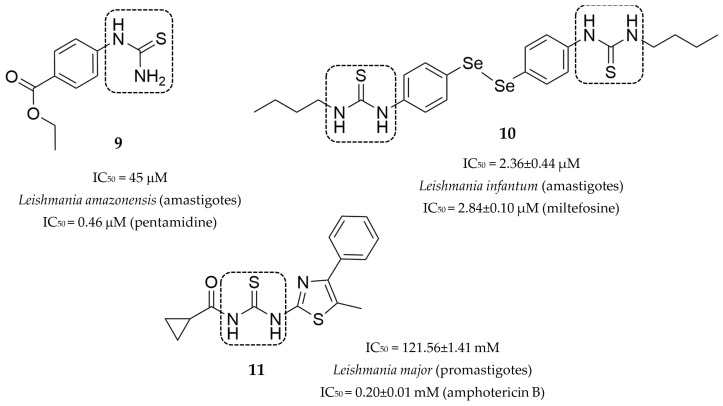
Thiourea derivatives as anti-leishmanial agents previously described in the literature [17,29,30].

Our research group previously demonstrated the leishmanicidal activity of several thiourea derivatives [18], identifying compound **8** as a prototype (Figure 3). This compound exhibited an IC_50_ value of 70.0 µM against *Leishmania amazonensis* amastigotes, with minimal cytotoxicity observed in host macrophages, and it showed greater potency than that of meglumine antimoniate (IC_50_ 212.30 µM). It is noteworthy that additional studies have also highlighted the leishmanicidal activity of thiourea derivatives against various *Leishmania* species. Boechat et al. (2013) synthesized several compounds from different classes with leishmanicidal activity against *L. amazonensis* promastigotes. Among them, thiourea **9** (Figure 4) was selected as a prototype (IC_50_ 45 µM), despite showing lower activity compared to that of pentamidine (IC_50_ 0.46 μM), emphasizing the thiourea class as a promising group of leishmanicidal agents [29].

In another study, Díaz et al. (2019) synthesized a series of *N*-substituted thioureas containing selenium and evaluated their cytotoxic activity against *Leishmania infantum* amastigotes, with selectivity assessed in human THP-1 cells. The most active thiourea derivative (compound **10**; Figure 4) exhibited anti-leishmanial activity (IC_50_ 2.36 ± 0.44 µM; SI = 10.6), which was comparable to that of the reference drug miltefosine (IC_50_ 2.84 ± 0.10 µM; SI = 7), but with a higher selectivity index [30]. Mohammadi-Ghalehbin et al. (2023) prepared a series of novel thiourea derivatives and subjected them to in vitro anti-leishmanial evaluation against *Leishmania major* promastigotes. Compound **11** (IC_50_ 121.56 ± 1.41 µM) exhibited the highest activity after 48 h (Figure 4) but was less potent than amphotericin B (IC_50_ 0.20 ± 0.01 µM), which was used as a positive control. Nevertheless, the potential of thioureas as a promising class in the search for new leishmanicidal agents was once again demonstrated [17].

Aligning with the correlation analysis presented in Table 8, in the second generation of thioureas (**G2**), the introduction of the benzhydryl group with piperazine, a bulky, hydrophobic moiety featuring two aromatic rings, into thioureas **5g**–**5i** and **5t** resulted in the formation of compounds with increased lipophilicity. Furthermore, when comparing **5a**–**5q** to **5r**–**5v** thioureas, the presence of a trimethoxy substituent within the R worsened the overall activity, indicating that the presence of heteroatoms negatively influences the compound’s potency despite both presenting aromatic groups. This trend aligns with the positive correlation existing between the NO ratio and heteroatoms, as indicated in Table 8.

In this series, increasing the spacer length (n) in thioureas **5g**, **5h**, and **5i** resulted in an increase in lipophilicity and, consequently, antipromastigote activity. Despite the absence of a spacer (n = 0) in compound **5t**, the presence of three methoxyl groups slightly increased its LogP value, thereby enhancing its potency. Although a similar trend can be observed in the antiamastigote activity of piperazine thioureas **5h**, **5i**, and **5t**, it is not possible to establish a valid correlation in structure–activity relationship studies due to the low number of active compounds against this form of the parasite.

According to ADMET Predictor^TM^, ≅90% of commercial drugs available in the WDI subset possess ADMET risk ≤ 7, TOX risk ≤ 2, Absn risk ≤ 5, and CYP risk ≤ 2. These cut-offs serve as reference values to guide the evaluation of candidate compounds, ensuring they meet safety and bioavailability standards for oral administration. In this context, the pharmacokinetic and toxicological profiles of thiourea derivatives with anti-leishmanial activity were assessed using ADMET Predictor™ models and presented in Table S3 (Supplementary Materials). Based on the general ADMET risk model, all active thioureas synthesized by our group meet the safety and bioavailability criteria for oral administration, as none of them exceeded the cut-off value of 7 (Table S3).

Overall, compounds presenting the highest ADMET risk values, like **5h**, **5i**, and **5t**, triggered risk flags primarily due to high lipophilicity (Kow), low water solubility (Sw), and elevated clearance by cytochrome P450 enzymes, particularly CYP2D6 and CYP3A4. Despite that, an assessment of individual models revealed that these compounds exhibit a favorable absorption profile, with relatively low absorption risks (≤4), suggesting their potential for favorable oral bioavailability. Conversely, **5g**, **5h**, **5i**, **5k**, and **5l** raised concerns about possible metabolic issues due to elevated intrinsic clearance (CYP risk > 2). This characteristic can reduce the compound’s half-life, potentially compromising its activity at the target organ and leading to suboptimal therapeutic levels.

Despite the potential metabolic drawbacks, compounds **5g**, **5h**, **5i**, **5k**, and **5l** were predicted to exhibit minimal toxicity (TOX Risk ≤ 2), along with favorable absorption profiles for oral administration (Absn Risk ≤ 4), suggesting their potential to mitigate these shortcomings. Notably, thiourea **5i**, the most active compound, presented a global risk (ADMET risk) within 90% of the empirical distribution derived from the WDI, thus being similar to compounds expected for commercial drugs. It may be, therefore, a promising strategy for the development of a potent and safe anti-leishmanial compound.

The leishmanicidal activity of the molecules studied here represents a novel contribution, as no previous reports describe their biological activity against *L. amazonensis*. The piperazine thiourea scaffold not only introduces a new class of leishmanicidal compounds but also offers a promising foundation for developing more potent and selective drug candidates as therapies for leishmaniasis. While these studies provide valuable insights, future research could expand by testing these series of molecules against other *Leishmania* species of clinical relevance, given the diversity of species that cause leishmaniasis. Additionally, exploring in vivo disease models and investigating the mechanism of action of these molecules against *Leishmania* parasites offer exciting opportunities for further advancing our understanding and therapeutic development.

The results of thiourea **5i** indicate a promising profile, making it a strong candidate for future tests aimed at evaluating its spectrum of action, pharmacokinetic profile, and efficacy in in vivo models. This derivative demonstrated both adequate potency and selectivity. When compared with other thiourea derivatives and piperazine-containing derivatives mentioned in Figure 1, it is evident that we have obtained a more potent and selective prototype relative to those previously reported.

## 4. Materials and Methods

### 4.1. Synthesis of Thioureas

#### 4.1.1. General

The reagents and solvents used in the experiments were obtained from commercial sources and used without further purification. ^1^H and ^13^C-NMR spectra were realized on a Bruker 400 or 500 MHz spectrometer using chloroform (CDCl_3_) or dimethyl sulfoxide (DMSO-*d*_6_) as solvent. Chemical shifts were given in ppm (*δ* scale) and coupling constants (*J*) were given in hertz (Hz). The Infrared (IR) spectra was performed on a Shimadzu IRPrestige-21 FTIR spectrometer using anhydrous KBr pellets. High-resolution mass spectrometry (HR-MS) was recorded on a Bruker micrOTOF II mass spectrometer using electrospray ionization (ESI). The analysis of the melting point was carried out on a SHIMADZU DSC-60 thermal analyzer with a heating rate of 10 °C/min, room temperature to 300 °C, under a nitrogen flow rate of 50 mL/min, and using an aluminum standard. Analytical Thin-Layer Chromatography (TLC) was performed on precoated silica gel plates (aluminum sheets 60 F254, Merck, Darmstadt, Germany) using ethyl acetate-hexane (1:5 *v*/*v*) as the eluent.

#### 4.1.2. Preparation of First-Generation (G1) Thioureas

Method A1: To a solution of isothiocyanate **1** (1.0 mmol; Scheme 1) in dichloromethane (10 mL), the corresponding amine **2** (1.2 mmol) was added, and the mixture was stirred at room temperature until isothiocyanate was consumed (TLC). After that, the reaction mixture was washed with 5% HCl_(aq)_ (3 × 10 mL), dried with anhydrous Na_2_SO_4_, and evaporated in a rotary evaporator to afford the pure thiourea **3**, which did not require further purification.

Method A2: In this method, the respective amine **2** (1.0 mmol) was added in a mixture of isothiocyanate **1** (1.2 mmol) and dichloromethane (10 mL) and was stirred at room temperature until amine was consumed (TLC). The solvent was then evaporated, and the crude product was washed at least three times with hexane until the excess of isothiocyanate was removed (TLC).

Method B1: To a solution of isothiocyanate **1** (1.0 mmol) in *tert*-butanol (10 mL), the corresponding amine **2** (1.2 mmol) was added, and the mixture was refluxed until isothiocyanate was consumed (TLC). Then, the solvent was evaporated, 10 mL of CH_2_Cl_2_ was added, and the same workup used in method A1 was followed.

Method B2: To a solution of isothiocyanate **1** (1.2 mmol) in *tert*-butanol (10 mL), the corresponding amine **2** (1.0 mmol) was added, and the mixture was refluxed until the amine was consumed (TLC). Then, the solvent was evaporated and the same workup used in method A2 was followed.

#### 4.1.3. Preparation of Second-Generation (G2) Thioureas

To a solution of isothiocyanate **1** (1.1 mmol; Scheme 2) in dichloromethane (20 mL), the corresponding piperazine **4** (1.0 mmol) was added, and the mixture was stirred at room temperature for 1 h. The solvent was then evaporated, and the crude product was washed at least three times with hexane until the excess of isothiocyanate was removed (TLC).

All structures of thioureas **3** and **5** were confirmed by ^1^H-NMR, ^13^C-NMR, FT-IR, and HR-MS (Supplementary Materials). The Supplementary Materials provide the characterization data for all thiourea derivatives, with the exception of compounds **3c**, **3d**, **3e**, **3g**, **3h**, **3m**, **3o**, **3p**, **3v**, **3w, 5a**, **5b**, **5d**, **5e**, **5j**, **5m**, **5n**, and **5q**, as these data have been previously reported by our research group [24].

### 4.2. Biological Assays

#### 4.2.1. Stock Solutions of Thioureas

The thioureas (**3a**–**3b′** and **5a**–**5v**) were solubilized in dimethyl sulfoxide (DMSO) to obtain a stock solution of 50 mmol/L and stored at −20 °C.

#### 4.2.2. Parasite Culture

*Leishmania amazonensis* promastigotes (strain MHOM/BR/77/LTB0016) were routinely obtained from the lesion of BALB/c mice and maintained in vitro as promastigotes to, at most, the fifth passage and maintained at 26 °C in Schneider’s medium supplemented with 10% fetal bovine serum (FBS), 100 μg/mL streptomycin, and 100 U/mL penicillin. Studies in *L. amazonensis*-infected BALB/c mice were performed following the guidelines of the Guide for the Care and Use of Laboratory Animals of the Brazilian National Council of Animal Experimentation (COBEA). This study was approved by the Animal Ethics Committee of Oswaldo Cruz Institute (L26/2015).

#### 4.2.3. Antipromastigote Assay

Promastigotes of *L. amazonensis* at 1 × 10^6^ cells/mL were incubated with different concentrations of thiourea compounds (0–100 µM) for 72 h at 26 °C. The assays were performed in triplicate in 96-well plates (Costar, New York, NY, USA). Inhibition parasite growth was assessed by a fluorescent assay, resazurin (Sigma-Aldrich, St. Louis, MO, USA) [31]. Briefly, 50 µM of resazurin (final concentration) was added per well, and then the samples were incubated for an additional 3 h. The fluorescence was measured using a Spectra Max GEMINI XPS spectrofluorometer (Molecular Devices, Silicon Valley, CA, USA) at excitation and emission wavelengths of 560 nm and 590 nm, respectively. IC_50_ value was calculated by nonlinear regression using Graph Pad Prism 9.0.

#### 4.2.4. Toxicity Assay in Murine Macrophages

Mouse peritoneal macrophages (2 × 10^6^ cells/mL) in 96-well plates were treated with different concentrations of thiourea compounds (G1: 0–400 µM and G2: 0–125 µM) for 72 h at 37 °C/5% CO_2_. After removing the supernatant, viable cells were quantified by adding resazurin in phosphate-buffered saline, a final concentration of 50 µM, and then the samples were incubated for an additional 3 h. The fluorescence was measured using a Spectra Max GEMINI XPS spectrofluorometer (Molecular Devices, Silicon Valley, CA, USA) at excitation and emission wavelengths of 560 nm and 590 nm, respectively. The percentage of viable cells was calculated relative to the control cells. The cytotoxic concentrations lethal to 50% of the cells (CC_50_) were obtained by nonlinear regression of the sigmoid growth curves using the Graph Pad Prism 9.0 software.

#### 4.2.5. Antiamastigote Assay

Resident macrophages from BALB/c mice were obtained by peritoneal lavage with 5 mL of cold RPMI medium. The cell suspension was adjusted to a 2 × 10^6^ cells/mL concentration and plated in LAB-TEK chambers. After 1 h, the cultures were washed with phosphate-buffered saline at 37 °C to remove non-adherent cells. The remaining cells were incubated at 37 °C/5% CO_2_ with promastigotes of *L. amazonensis* at a ratio of 3:1. After 3 h, the chambers were rewashed to remove free parasites. The monolayers were incubated with different concentrations of thiourea compounds (0–10 µM) for 72 h at 37 °C/5% CO_2_. For G2, we first tested only at a concentration of 10 µM, and derivatives with inhibition above 60% were evaluated using the dose–response curve. The antiamastigote activity was evaluated microscopically after staining the chambers with the Instant Prov hematological dye system (Newprov, Curitiba, Brazil); at least 100 macrophages were counted per sample. The results were expressed as the infection index (IF) using the following formula: IF = % infected cells X (number of amastigotes/total macrophages). The IC_50_ value was determined by nonlinear regression using Graph Pad Prism 9.0.

### 4.3. Structure–Activity Relationship Analysis

The scipy package available in Python [32] was used to calculate Pearson correlation coefficients between the experimental activity of anti-leishmanial compounds (IC_50_) and their corresponding physiochemical parameters as predicted by the ACD/Percepta software, version 14.52.0 (Advanced Chemistry Development. Inc., Toronto, ON, Canada).

### 4.4. In Silico Pharmacokinetics and Toxicological Assessment

The pharmacokinetics and toxicity profile of the anti-leishmanial thiourea compounds were characterized in silico using the ADMET Predictor™ 10.4 (Simulations Plus, Inc., Lancaster, CA, USA). This software integrates highly accurate QSAR (Quantitative Structure–Activity Relationship) models, typically yielding R^2^ values between 0.7 and 0.9, into a risk assessment system that establishes cut-off points based on the analysis of 2260 commercial drugs from the World Drug Index (WDI). As a result, chemical compounds that exceed the established safety or efficacy limits for commercial drugs incur risk penalties. The overall ADMET risk factor combines three statistical models: TOX risk (toxicity), Absn risk (absorption), and CYP risk (metabolism), in addition to two distribution/excretion parameters: volume of distribution (Vd) and fraction unbound (Fu). These components collectively provide a comprehensive evaluation of a compound’s pharmacokinetic and toxicological profiles.

Hepatotoxicity, mutagenicity, carcinogenicity, cardiotoxicity, and acute toxicity were combined into a toxicity model, henceforth called TOX risk. Hepatotoxicity was estimated by assessing the serum levels of liver-damage-related enzymes associated with hepatic damage, including alanine aminotransferase (ALT), aspartate aminotransferase (AST), lactate dehydrogenase (LDH), alkaline phosphatase (ALP), and gamma-glutamyltransferase (GGT). Mutagenicity was assessed using Ames’s test models, which simulate in silico assays with a variety of *Salmonella typhimurium* strains (i.e., TA97, TA98, TA100, TA1535, and TA1537), as well as the repair-deficient *Escherichia coli* strain WP2, both with and without activation by the metabolic S9 fraction. Acute rat toxicity was estimated based on the amount of orally administered chemicals required to kill half of the rats tested within 24 h. The cardiotoxicity model was based on hERG (Ether-à-go-go-Related Gene) inhibition. Carcinogenicity was evaluated using rat- and mouse-based models considering a chronic oral daily dose administration required to produce tumors in 50 percent of the population.

The absorption risk (Absn risk) model was used to better understand the potential oral absorption problems the anti-leishmanial thiourea compounds might have based on their physiochemical characteristics: molar weight (MW, g/mol), octanol/water partition coefficient (logP), octanol/water distribution coefficient at pH 7.4 (logD), topological polar surface area (TPSA), number of hydrogen bond donors (HBDs), hydrogen bond acceptors (HBAs), number of rotatable bonds, and the ionization constant (pKa) of the most basic chemical group. At last, the metabolic risk (CYP risk) was assessed based on the compounds’ oxidation by cytochrome P450 (CYP) enzymes. Compounds are flagged as high-risk if they exhibit elevated intrinsic clearance levels, assessed through recombinant assays for CYP1A2, CYP2C9, CYP2C19, CYP2D6, and CYP3A4, in addition to a human liver microsomal model.

## 5. Conclusions

In summary, our study highlights the potential of thiourea derivatives as promising candidates for anti-leishmanial drug development, particularly against *Leishmania amazonensis*. Compound **3e** from the first generation and compound **5i** from the second generation demonstrated significant activity, with **5i** showing both high potency and selectivity. SAR analysis revealed that the structural features, such as carbon spacer length and lipophilicity, are key to optimizing anti-leishmanial activity. Moreover, ADMET predictions confirmed that compound **5i** possesses a favorable pharmacokinetic and toxicity profile, suggesting its potential for further in vivo studies. These findings provide valuable insights for the development of potent and safe anti-leishmanial agents.

## Figures and Tables

**Table 1:** Reaction conditions and yields for first-generation thioureas **3a**–**3b′**.

Thiourea ^a^	Method ^b^	Time (h)	Yield (%) ^c^	R	R_1_	R_2_	n
**3a**	A1	2.0	70	H	4-Morpholinyl	-	0
**3b**	A2	2.0	99	H	4-Morpholinyl	-	1
**3c**	A1	2.0	70	H	4-Morpholinyl	-	2
**3d**	B2	20.0	92	H	Pyridin-2-yl	H	0
**3e**	B2	8.0	99	H	Pyridin-2-yl	H	1
**3f**	B2	7.0	76	H	Pyridin-2-yl	H	2
**3g**	B2	4.0	96	H	4-OMe-Ph	H	0
**3h**	B2	3.0	97	H	4-OMe-Ph	H	1
**3i**	B1	2.0	70	H	3,4-OMe-Ph	H	1
**3j**	B1	3.0	99	H	3,4-OMe-Ph	H	2
**3k**	B1	2.0	76	H	3,5-OMe-Ph	H	1
**3l**	B1	6.0	75	H	3,5-OMe-Ph	H	2
**3m**	B1	3.0	99	H	3,4,5-OMe-Ph	H	2
**3n**	A2	6.0	70	H	NHCOPh	H	0
**3o**	A2	6.0	70	H	NHCOPh	H	1
**3p**	B2	2.0	96	H	4-Br-Ph	H	0
**3q**	B2	3.0	92	H	4-Br-Ph	H	1
**3r**	B1	3.0	97	H	5-Me-1,3,4-thiadiazol-2-yl	H	0
**3s**	B1	2.0	80	H	5-Et-1,3,4-thiadiazol-2-yl	H	1
**3t**	A1	1.0	75	H	Bu	H	0
**3u**	B1	2.0	75	H	2-Me-Ph	H	1
**3v**	B2	3.0	99	H	3-OH-Ph	H	1
**3w**	A2	4.0	92	H	(*S*,*R*,*R*,*R*)-CH_2_[CH(OH)]_4_CH_2_OH	Me	1
**3x**	A1	2.0	89	OMe	4-Morpholinyl	-	0
**3y**	A2	10.0	76	OMe	Pyridin-2-yl	H	0
**3z**	B1	6.0	94	OMe	3,4-OMe-Ph	H	0
**3a′**	B1	4.0	95	OMe	5-CF_3_-1,3,4-thiadiazol-2-yl	H	0
**3b′**	A1	2.0	94	OMe	Bu	H	0

^a^ Products were characterized by physical and spectroscopic methods. ^b^ Method A1: isothiocyanate (1.0 mmol), amine (1.2 mmol), dichloromethane, r.t. Method A2: isothiocyanate (1.2 mmol), amine (1.0 mmol), dichloromethane, r.t. Method B1: isothiocyanate (1.0 mmol), amine (1.2 mmol), tert-butanol, reflux. Method B2: isothiocyanate (1.2 mmol), amine (1.0 mmol), tert-butanol, reflux. ^c^ Isolated yield.

**Table 2:** Reaction conditions and yields for second-generation thioureas **5a**–**v**.

Thiourea ^a^	Yield (%) ^b^	R	R_3_	n
**5a**	72	H	Tetrahydrofuran-2-carbonyl	0
**5b**	90	H	Tetrahydrofuran-2-carbonyl	1
**5c**	99	H	Tetrahydrofuran-2-carbonyl	2
**5d**	99	H	3,4-Methylenedioxy-benzyl	0
**5e**	99	H	3,4-Methylenedioxy-benzyl	1
**5f**	93	H	3,4-Methylenedioxy-benzyl	2
**5g**	99	H	Benzhydryl	0
**5h**	99	H	Benzhydryl	1
**5i**	99	H	Benzhydryl	2
**5j**	99	H	2-Cl-Ph	0
**5k**	99	H	2-Cl-Ph	1
**5l**	97	H	2-Cl-Ph	2
**5m**	93	H	2,3,4-OMe-Benzyl	0
**5n**	92	H	2,3,4-OMe-Benzyl	1
**5o**	76	H	2,3,4-OMe-Benzyl	2
**5p**	86	H	Benzoimidazol-2-yl	0
**5q**	85	H	Benzoimidazol-2-yl	2
**5r**	95	OMe	Tetrahydrofuran-2-carbonyl	0
**5s**	99	OMe	3,4-Methylenedioxy-benzyl	0
**5t**	99	OMe	Benzhydryl	0
**5u**	99	OMe	2-Cl-Ph	0
**5v**	70	OMe	2,3,4-OMe-Benzyl	0

Method: isothiocyanate (1.1 mmol), piperazine (1.0 mmol), dichloromethane, r.t., 1.0 h. ^a^ Products were characterized by physical and spectroscopic methods. ^b^ Isolated yield.

**Table 3:** Activity of the thiourea derivatives (G1) against *L. amazonensis* promastigote.

Thiourea	IC_50_ (µM)
**3a**	>100 ^a^
**3b**	>100 ^a^
**3c**	>100 ^a^
**3d**	19.9 ± 3.9
**3e**	25.4 ± 8.3
**3f**	>100 ^a^
**3g**	>100 ^a^
**3h**	61.6 ± 6.5
**3i**	>100 ^a^
**3j**	>100 ^a^
**3k**	>100 ^a^
**3l**	>100 ^a^
**3m**	>100 ^a^
**3n**	77.0 ± 6.8
**3o**	80.4 ± 10.0
**3p**	32.9 ± 9.5
**3q**	44.4 ± 10.6
**3r**	65.8 ± 8.2
**3s**	58.4 ± 1.9
**3t**	60.0 ± 6.4
**3u**	56.2 ± 4.2
**3v**	>100 ^a^
**3w**	>100 ^a^
**3x**	>100 ^a^
**3y**	>100 ^a^
**3z**	>100 ^a^
**3a′**	77.8 ± 6.7
**3b′**	>100 ^a^
**Miltefosine**	6.9 ± 2.4

^a^ Compounds did not show activity up to the maximum concentration tested (100 µM).

**Table 4:** Antiamastigote activity and murine macrophage toxicity of the thiourea derivatives (G1).

Thiourea	IC_50_ (μM)	CC_50_	SI
**3d**	10.8 ± 2.4	>400	>37
**3e**	4.9 ± 1.2	>400	>81.6
**3p**	8.7 ± 1.4	>400	>45.9
**3q**	>25 ^a^	>400	-
**Miltefosine**	7.5 ± 1.2	109.7 ± 2.0	14.6

^a^ Compounds did not show activity up to the maximum concentration tested (25 µM).

**Table 5:** Activity of the thiourea derivatives (G2) against *L. amazonensis* promastigote.

Thiourea	IC_50_ (µM)
**5a**	>128
**5b**	77.5 ± 4.5
**5c**	>128
**5d**	90.2 ± 2.9
**5e**	30.3 ± 4.6
**5f**	>128
**5g**	30.7 ± 3.2
**5h**	7.9 ± 1.9
**5i**	6.9 ± 2.0
**5j**	>128
**5k**	30.7 ± 3.0
**5l**	28.2 ± 2.5
**5m**	>128
**5n**	42.5 ± 1.6
**5o**	>128
**5p**	>128
**5q**	18.8 ± 3.1
**5r**	>128
**5s**	>128
**5t**	22.3 ± 1.6
**5u**	>128
**5v**	>128
**Miltefosine**	6.9 ± 2.4

**Table 6:** Antiamastigote activity and murine macrophage toxicity of the thiourea derivatives (G2).

Thiourea	IC_50_	CC_50_	SI
**5e**	>10 ^a^	121.8 ± 2.8	-
**5g**	>10 ^a^	>125	-
**5h**	8.8 ± 3.5	>125	>14.2
**5i**	1.8 ± 0.5	>125	>69.4
**5k**	>10 ^a^	68.3 ± 2.8	-
**5l**	>10 ^a^	79.5 ± 19.8	-
**5n**	>10 ^a^	88.3 ± 5.6	-
**5q**	>10	72.9 ± 32.2	
**5t**	4.9 ± 1.2	81.3 ± 16.9	16.6
**Miltefosine**	7.5 ± 1.2	109.7 ± 2.0	14.6

^a^ Compounds did not show activity up to the maximum concentration tested (10 µM).

**Table 7:** Correlation between the experimental IC_50_ for the active thiourea compounds G1 against *L. amazonensis* promastigotes and their corresponding physiochemical parameters predicted by ACD/Percepta.

Physiochemical Parameter	Pearson Correlation Coefficient	*p*-Value
Hydrogen Bond Acceptors	0.65756	0.02012
Rotatable Bonds	0.58450	0.04594
Hydrogen Bond Donors	0.58297	0.04665
NO Ratio	0.55691	0.05998
TPSA	0.5475	0.06536
Parachor (cm^3^)	0.52447	0.08001
Molar Volume (cm^3^)	0.49650	0.10060
Hetero Ratio	0.44640	0.1457
C Ratio	−0.40422	0.19248
Molar Refractivity	0.36363	0.24526
Polarizability	0.36363	0.24526
Isoelectric point	−0.3496	0.26523
Surface Tension (dyne/cm)	−0.32867	0.29690
Molecular Weight	0.32867	0.29690
Log(BCF)	0.23076	0.47053
Log(Koc)	0.23076	0.47053
LogP	0.23076	0.47053
Number of Aromatic Rings	−0.04367	0.89280
N Ratio	0.03880	0.90470

**Table 8:** Correlation between the experimental IC_50_ for the active thiourea compounds **G2** against *L. amazonensis* promastigotes and their corresponding physiochemical parameters predicted by ACD/Percepta.

Physiochemical Parameter	Pearson Correlation Coefficient	*p*-Value
LogP	−0.8246	0.00178
Log(Koc)	−0.8246	0.00178
Log(BCF)	−0.8246	0.00178
C Ratio	−0.7517	0.0076
Number of Aromatic Rings	−0.7504	0.00779
NO Ratio	0.7133	0.01371
Hetero Ratio	0.70346	0.01571
Molar Refractivity	−0.6833	0.0204
Polarizability	−0.6833	0.02044
Parachor (cm^3^)	−0.6059	0.04816
Molar Volume (cm^3^)	−0.6013	0.05033
Molecular Weight	−0.5785	0.06220
Rotatable Bonds	−0.5592	0.07365
Isoelectric point	−0.4100	0.21040
Surface Tension (dyne/cm)	0.36446	0.27046
Hydrogen Bond Acceptors	0.33915	0.30757
Hydrogen Bond Donors	−0.30068	0.36893
N Ratio	0.25585	0.44762
TPSA	0.19209	0.5715

## Data Availability

Data are contained within the article and the Supplementary Materials.

## Supplementary Materials

“References [33,34,35,36,37,38,39,40] are cited in the Supplementary Materials” The following supporting information can be downloaded at https://www.mdpi.com/article/10.3390/ph17121573/s1, Figure S1: ^1^H-NMR spectrum (A) and ^13^C-NMR spectrum (B) of thiourea 3a; Figure S2: ^1^H-NMR spectrum (A) and ^13^C-NMR spectrum (B) of thiourea 3b; Figure S3: ^1^H-NMR spectrum (A) and ^13^C-NMR spectrum (B) of thiourea 3f; Figure S4: ^1^H-NMR spectrum (A) and ^13^C-NMR spectrum (B) of thiourea 3i; Figure S5: ^1^H-NMR spectrum (A) and ^13^C-NMR spectrum (B) of thiourea 3j; Figure S6: ^1^H-NMR spectrum (A) and ^13^C-NMR spectrum (B) of thiourea 3k; Figure S7: ^1^H-NMR spectrum (A) and ^13^C-NMR spectrum (B) of thiourea 3l; Figure S8: ^1^H-NMR spectrum (A) and ^13^C-NMR spectrum (B) of thiourea 3n; Figure S9: ^1^H-NMR spectrum (A) and ^13^C-NMR spectrum (B) of thiourea 3q; Figure S10: ^1^H-NMR spectrum (A) and ^13^C-NMR spectrum (B) of thiourea 3r; Figure S11: FT-IR spectrum of thiourea 3r; Figure S12: HR-MS spectrum of thiourea 3r; Figure S13: ^1^H-NMR spectrum (A) and ^13^C-NMR spectrum (B) of thiourea 3s; Figure S14: FT-IR spectrum of thiourea 3s; Figure S15: HR-MS spectrum of thiourea 3s; Figure S16: ^1^H-NMR spectrum (A) and ^13^C-NMR spectrum (B) of thiourea 3t; Figure S17: ^1^H-NMR spectrum (A) and ^13^C-NMR spectrum (B) of thiourea 3u; Figure S18: ^1^H-NMR spectrum (A) and ^13^C-NMR spectrum (B) of thiourea 3x; Figure S19: ^1^H-NMR spectrum (A) and ^13^C-NMR spectrum (B) of thiourea 3y; Figure S20: ^1^H-NMR spectrum (A) and ^13^C-NMR spectrum (B) of thiourea 3z; Figure S21: ^1^H-NMR spectrum (A) and ^13^C-NMR spectrum (B) of thiourea 3a’; Figure S22: FT-IR spectrum of thiourea 3a’; Figure S23: HR-MS spectrum of thiourea 3a’; Figure S24: ^1^H-NMR spectrum (A) and ^13^C-NMR spectrum (B) of thiourea 3b’; Figure S25: FT-IR spectrum of thiourea 3b’; Figure S26: HR-MS spectrum of thiourea 3b’; Figure S27: ^1^H-NMR spectrum (A) and ^13^C-NMR spectrum (B) of thiourea 5c; Figure S28: FT-IR spectrum of thiourea 5c; Figure S29: HR-MS spectrum of thiourea 5c; Figure S30: ^1^H-NMR spectrum (A) and ^13^C-NMR spectrum (B) of thiourea 5f; Figure S31: FT-IR spectrum of thiourea 5f; Figure S32: HR-MS spectrum of thiourea 5f; Figure S33: ^1^H-NMR spectrum (A) and ^13^C-NMR spectrum (B) of thiourea 5g; Figure S34: ^1^H-NMR spectrum (A) and ^13^C-NMR spectrum (B) of thiourea 5h; Figure S35: FT-IR spectrum of thiourea 5h; Figure S36: HR-MS spectrum of thiourea 5h; Figure S37: ^1^H-NMR spectrum (A) and ^13^C-NMR spectrum (B) of thiourea 5i; Figure S38: FT-IR spectrum of thiourea 5i; Figure S39: HR-MS spectrum of thiourea 5i; Figure S40: ^1^H-NMR spectrum (A) and ^13^C-NMR spectrum (B) of thiourea 5k; Figure S41: FT-IR spectrum of thiourea 5k; Figure S42: HR-MS spectrum of thiourea 5k; Figure S43: ^1^H-NMR spectrum (A) and ^13^C-NMR spectrum (B) of thiourea 5l; Figure S44: FT-IR spectrum of thiourea 5l; Figure S45: HR-MS spectrum of thiourea 5l; Figure S46: ^1^H-NMR spectrum (A) and ^13^C-NMR spectrum (B) of thiourea 5o; Figure S47: FT-IR spectrum of thiourea 5o; Figure S48: HR-MS spectrum of thiourea 5o; Figure S49: ^1^H-NMR spectrum (A) and ^13^C-NMR spectrum (B) of thiourea 5p; Figure S50: FT-IR spectrum of thiourea 5p; Figure S51: HR-MS spectrum of thiourea 5p; Figure S52: ^1^H-NMR spectrum (A) and ^13^C-NMR spectrum (B) of thiourea 5r; Figure S53: FT-IR spectrum of thiourea 5r; Figure S54: HR-MS spectrum of thiourea 5r; Figure S55: ^1^H-NMR spectrum (A) and ^13^C-NMR spectrum (B) of thiourea 5s; Figure S56: FT-IR spectrum of thiourea 5s; Figure S57: HR-MS spectrum of thiourea 5s; Figure S58: ^1^H-NMR spectrum (A) and ^13^C-NMR spectrum (B) of thiourea 5t; Figure S59: FT-IR spectrum of thiourea 5t; Figure S60: HR-MS spectrum of thiourea 5t; Figure S61: ^1^H-NMR spectrum (A) and ^13^C-NMR spectrum (B) of thiourea 5u; Figure S62: FT-IR spectrum of thiourea 5u; Figure S63: HR-MS spectrum of thiourea 5u; Figure S64: ^1^H-NMR spectrum (A) and ^13^C-NMR spectrum (B) of thiourea 5v; Figure S65: FT-IR spectrum of thiourea 5v; Figure S66: HR-MS spectrum of thiourea 5v; Table S1: Physiochemical parameters predicted by ACD/Percepta for active thiourea compound 3 against *L. amazonensis* promastigotes and its respective experimental IC_50_; Table S2: Physiochemical parameters predicted by ACD/Percepta for active thiourea compound 5 against *L. amazonensis* promastigotes and its respective experimental IC_50_; Table S3: Pharmacokinetics and toxicological analysis of thiourea compounds with anti-leishmanial activity.
