# Supplementary material for: Synthesis and Structure–Activity Relationship of Thiourea Derivatives Against Leishmania amazonensis
Source: Pharmaceuticals (Basel). 2024 Nov 23;17(12):1573. doi: 10.3390/ph17121573 (PMC11677126; doi:10.3390/ph17121573)
Supplement: Supplementary file 1 [file pharmaceuticals-17-01573-s001.zip › pharmaceuticals-3306902-supplementary.pdf]

Supporting Information

Synthesis and Structure–Activity Relationship of Thiourea Derivatives Against *Leishmania amazonensis*

Gil Mendes Viana [1], Edézio Ferreira da Cunha-Junior [2], Paloma Wetler Meireles Carreiros Assumpção [1], Marianne Grilo Rezende [1], Yago Sousa dos Santos Emiliano[3], Laiza Maria da Silva Soares [3], Gabriel Rodrigues Coutinho Pereira [4], Carlos Rangel Rodrigues [4], Lucio Mendes Cabral [1,*] and Eduardo Caio Torres-Santos [3,*]

1 Laboratório de Tecnologia Industrial Farmacêutica, Faculdade de Farmácia, Universidade Federal do Rio de Janeiro, 21941-590 Rio de Janeiro, Brazil; gmviana@gmail.com (G.M.V.); palomawetler@yahoo.com.br (P.W.M.C.A.); mariannegrezende@gmail.com (M.G.R.)
2 Laboratório de Imunoparasitologia, Unidade Integrada de Pesquisa em Produtos Bioativos e Biociências, Centro Multidisciplinar UFRJ-Macaé, Universidade Federal do Rio de Janeiro, 27970-000 Macaé, Brazil; edezio@macae.ufrj.br
3 Laboratório de Bioquímica de Tripanosomatídeos, Instituto Oswaldo Cruz, Fundação Oswaldo Cruz, 21040-900 Rio de Janeiro, Brazil; yago.emiliano@ioc.fiocruz.br (Y.S.d.S.E.); laizamss@hotmail.com (L.M.d.S.S.)
4 Laboratório ModMolQSAR, Faculdade de Farmácia, Universidade Federal do Rio de Janeiro, 21941-590 Rio de Janeiro, Brazil; gabrielrodriguescp@gmail.com (G.R.C.P.); rangelfarmacia@gmail.com (C.R.R.)
* Correspondence: lmcabral2@yahoo.com.br (L.M.C.); ects@ioc.fiocruz.br (E.C.T.-S.)

1 - Characterization data of Thiourea Derivatives (3 and 5)

Option 1 (These are the data as typically described when reporting the synthesis of substances)

N-phenylmorpholine-4-carbothioamide (**3a**) [1] White solid; 130-132 ºC; IR (KBr): 3160, 3090, 3020, 2910, 2850, 1590, 1530, 1460, 1400, 1320, 1200, 935 cm^{-1}; ^{1}H-NMR (500 MHz, DMSO-d_6) δ 9.36 (br s, 1H), 7.30 (d, *J* = 4.22 Hz, 4H), 7.14-7.07 (m, 1H), 3.88 (t, *J* = 4.70 Hz, 4H), 3.65 (t, *J* = 4.70 Hz, 4H); ^{13}C-NMR (125 MHz, DMSO-d_6) δ 182.25, 141.39, 128.49, 125.73, 124.85, 66.25, 48.90; HR-MS-ESI: m/z [M+H]$^+$ calculated for $C_{11}H_{14}N_2OS$: 223.0890; found: 223.0900.

N-benzylmorpholine-4-carbothioamide (**3b**) [1] Pale yellow solid; 97-99 ºC; IR (KBr): 3246, 3043, 2962, 2852, 1603, 1537, 1448, 1336, 1196, 962, 694 cm^{-1}; ^{1}H-NMR (400 MHz, $CDCl_3$) δ: 7.45-7.15 (m, 5H), 5.75 (br s, 1H), 4.86 (d, *J* = 4.72 Hz, 2H), 3.86- 3.61 (m, 8H); ^{13}C-NMR (100 MHz, $CDCl_3$) δ: 182.76, 137.72, 128.86, 128.14, 127.87, 66.14, 50.38, 47.58; HR-MS-ESI: m/z [M+H]$^+$ calculated for $C_{12}H_{16}N_2OS$: 237.1044; found: 237.1057.

***N*-phenethyl-*N'*-(pyridin-2-yl)thiourea** (**3f**) [2] White solid; 154-156 ºC; IR (KBr): 3480, 3223, 2998, 1604, 1146, 1090, 1040, 868, 778 cm^{-1}; ^{1}H-NMR (400 MHz, $CDCl_3$) δ 11.78 (br s, 1H), 9.29 (br s, 1H), 7.95 (d, J = 4.07 Hz, 1H), 7.62-7.55 (m, 1H), 7.35-7.20 (m, 5H), 6.92-6.84 (m, 2H), 4.04 (q, *J* = 6.81 Hz, 2H), 3.02 (t, *J* = 6.92 Hz, 2H); ^{13}C-NMR (100 MHz, $CDCl_3$) δ 179.44, 153.42, 145.51, 138.98, 138.58, 128.99, 128.56, 126.55, 117.84, 112.09, 47.00, 35.00; HR-MS-ESI: m/z $[M+H]^+$ calculated for $C_{14}H_{15}N_3S$: 258.1059; found: 258.1060.

1-benzyl-3-(3,4-dimethoxyphenyl)thiourea (**3i**) [3] Purple solid; 149-151 ºC; IR (KBr): 3332, 3149, 2981, 1595, 1544, 1516, 1437, 1324, 1233, 1134, 1069, 1019, 960, 855, 789, 745, 700 cm^{-1}; ^{1}H-NMR (200 MHz, $CDCl_3$) δ 8.11 (br s, 1H), 7.34-7.23 (m, 5H), 6.87-6.69 (m, 3H), 6.23 (br s, 1H), 4.86 (d, *J* = 5.33 Hz, 2H), 3.84 (s, 3H), 3.78 (s, 3H); ^{13}C-NMR (50 MHz, $CDCl_3$) δ 180.57, 150.11, 148.67, 137.59, 128.89, 128.74, 127.85, 127.79, 118.53, 111.98, 109.88, 56.20, 56.13, 49.66; HR-MS-ESI: m/z $[M+H]^+$ calculated for $C_{16}H_{18}N_2O_2S$: 303.1154; found: 303.1162.

1-(3,4-dimethoxyphenyl)-3-phenethylthiourea (**3j**) [3] Purple solid; 184-186 ºC; IR (KBr): 3353, 3178, 3010, 2931, 2832, 1597, 1547, 1525, 1444, 1355, 1289, 1239, 1134, 1029, 842, 751, 702 cm^{-1}; ^{1}H-NMR (200 MHz, $CDCl_3$) δ 7.52 (br s, 1H), 7.27-7.06 (m, 5H), 6.81-6.73 (m, 1H), 6.65-6.54 (m, 2H), 5.85 (br s, 1H), 3.94-3.80 (m, 5H), 3.74 (s, 3H), 2.90 (t, *J* = 6.74 Hz, 2H); ^{13}C-NMR (50 MHz, $CDCl_3$) δ 181.26, 150.09, 148.75, 138.59, 128.80, 128.38, 126.72, 118.75, 111.80, 109.91, 56.22, 56.14, 46.32, 34.92; HR-MS-ESI: m/z $[M+H]^+$ calculated for $C_{17}H_{20}N_2O_2S$: 317.1308; found: 317.1318.

1-benzyl-3-(3,5-dimethoxyphenyl)thiourea (**3k**) [3] Beige solid; 124-126 ºC; IR (KBr): 3340, 3200, 3040, 3000, 2830, 1600, 1530, 1450, 1310, 1260, 1200, 1150, 1050, 924, 818, 698 cm^{-1}; ^{1}H-NMR (400 MHz, $CDCl_3$) δ 8.12 (br s, 1H), 7.37-7.24 (m, 5H), 6.51 (br s, 1H), 6.33 (s, 3H), 4.88 (d, *J* = 4.92 Hz, 2H), 3.76-3.65 (m, 6H); ^{13}C-NMR (100 MHz, $CDCl_3$) δ 180.52, 161.89, 137.62, 137.28, 128.81, 127.79, 127.74, 102.87, 99.20, 55.50, 49.51; HR-MS-ESI: m/z $[M+H]^+$ calculated for $C_{16}H_{18}N_2O_2S$: 303.1152; found: 303.1162.

1-(3,5-dimethoxyphenyl)-3-phenethylthiourea (**3l**) [3] White solid; 115-117 ºC; IR (KBr): 3350, 3159, 3005, 1686, 1610, 1587, 1525, 1443, 1315, 1223, 1190, 1153, 1005, 845, 661 cm^{-1}; ^{1}H-NMR (200 MHz, $CDCl_3$) δ 8.10 (br s, 1H), 7.32-7.09 (m, 5H), 6.35-6.29 (m, 1H), 6.28-6.15 (m, 3H), 3.88 (q, *J* = 6.40 Hz, 2H), 3.69 (s, 6H), 2.91 (t, *J* = 6.80 Hz, 2H); ^{13}C-NMR (50 MHz, $CDCl_3$) δ 180,45, 161.96, 138.62, 137.69, 128.84 (2C), 126.72, 103.15, 99.31, 55.63, 46.54, 34.97; HR-MS-ESI: m/z $[M+H]^+$ calculated for $C_{17}H_{20}N_2O_2S$: 317.1308; found: 317.1319.

2-benzoyl-N-phenylhydrazinecarbothioamide (**3n**) [4] White solid; 164-166 ºC; IR (KBr): 3302, 3221, 1641, 1606, 1556, 1497, 1462, 1361, 1231, 761, 715, 695 cm^{-1}; ^{1}H-NMR (500 MHz, DMSO-d_6) δ 10.65 (br s, 1H), 9.93 (br s, 1H), 9.82 (br s, 1H), 8.06 (d, *J* = 7.52 Hz, 2H), 7.68 (t, *J* = 7.36 Hz, 1H), 7.60 (t, *J* = 7.62 Hz, 2H), 7.54 (br s, 2H), 7.43 (t, *J* = 7.84 Hz, 2H), 7.26 (t, *J* = 7.36 Hz, 1H); ^{13}C-NMR (125 MHz, DMSO-d_6) δ 181.62, 166.46, 139.75, 133.02, 132.31, 128.71, 128.37 (2C), 126.52, 125.49; HR-MS-ESI: m/z $[M+H]^+$ calculated for $C_{14}H_{13}N_3OS$: 272.0852; found: 272.0852.

N-benzyl-N'-(4-bromophenyl)thiourea (**3q**) [5] Gray solid; 139-141 ºC; IR (KBr): 3397, 3153, 2981, 1536, 1515, 1301, 1236, 1069, 825, 739 cm^{-1}; ^{1}H-NMR (400 MHz, $CDCl_3$) δ 8.36 (br s, 1H), 7.51 (d, *J* = 8.06 Hz, 2H), 7.41-7.24 (m, 5H), 7.11 (d, *J* = 8.06 Hz, 2H), 6.29 (br s, 1H), 4.86 (d, *J* = 4.55 Hz, 2H); ^{13}C-NMR (100 MHz, $CDCl_3$) δ 180.72, 136.97, 135.20, 133.29, 128.90, 127.94, 127.74, 126.77, 120.71, 49.46; HR-MS-ESI: m/z $[M+H]^+$ calculated for $C_{14}H_{13}BrN_2S$: 321.0056; found: 321.0056.

1-(5-methyl-1,3,4-thiadiazol-2-yl)-3-phenylthiourea (**3r**) White solid; 219-221 ºC; IR (KBr): 3325, 3252, 3194, 2671, 1653, 1598, 1552, 1498, 1361, 1323, 1237, 1059, 752, 652 cm^{-1}; ^{1}H-NMR (200 MHz, DMSO-d_6) δ 10.46 (br s, 1H), 7.66 (d, *J* = 8.20 Hz, 2H), 7.33 (t, *J* = 7.72 Hz, 2H), 7.11 (t, *J* = 7.25 Hz, 1H), 2.67 (s, 3H); ^{13}C-NMR (50 MHz, DMSO-d_6) δ 183.57, 156.23, 140.00, 128.98, 124.81, 123.23, 119.45, 15.72. HR-MS-ESI: m/z $[M+H]^+$ calculated for $C_{10}H_{10}N_4S_2$: 251.0420; found: 251.2516.

N-benzyl-N′-(5-ethyl-1,3,4-thiadiazol-2-yl)thiourea **(3s)** White solid; 163-165 ºC; IR (KBr): 3341, 2973, 1632, 1533, 1454, 1379, 1348, 1276, 782, 699, 656 cm^{-1}; ^{1}H-NMR (500 MHz, DMSO-d_6) δ 7.54-749 (m, 2H), 7.48-7.36 (m, 3H), 4.99 (d, *J* = 5.15 Hz, 2H), 2.93 (q, *J* = 7.44 Hz, 2H), 1.36 (t, *J* = 7.44 Hz, 3H); ^{13}C-NMR (125 MHz, DMSO-d_6) δ 178.86, 164.50, 164.15, 136.79, 128.65, 128.19, 127.74, 49.37, 23.22, 13.55; HR-MS-ESI: m/z $[M+H]^+$ calculated for $C_{12}H_{14}N_4S_2$: 279.0733; found: 279.0726.

N-butyl-N′-phenylthiourea **(3t)** [6] White solid; 63-65 ºC; IR (KBr): 3295, 3171, 3003, 2950, 2925, 2857, 1596, 1551, 1534, 1321, 1242, 1066, 932, 693, 605 cm^{-1}; ^{1}H-NMR (200 MHz, $CDCl_3$) δ 8.21 (br s, 1H), 7.51-7.36 (m, 2H), 7.35-7.15 (m, 3H), 6.05 (br s, 1H), 3.62 (q, *J* = 6.47 Hz, 2H), 1.55 (quint, *J* = 7.17 Hz, 2H), 1.44-1.20 (m, 2H), 0.91 (t, *J* = 7.14 Hz, 3H); ^{13}C-NMR (50 MHz, $CDCl_3$) δ 180.57, 136.35, 130.27, 127.27, 125.28, 45.36, 31.12, 20.15, 13.83; HR-MS-ESI: m/z $[M+H]^+$ calculated for $C_{11}H_{16}N_2S$: 209.1097; found: 209.1107.

N-benzyl-N′-(o-tolyl)thiourea **(3u)** [7] Light brown solid; 136-138 ºC; IR (KBr): 3500, 3169, 2968, 1618, 1539, 1249, 970, 743, 642 cm^{-1}; ^{1}H-NMR (500 MHz, $CDCl_3$) δ 7.84 (br s, 1H), 7.33-7.18 (m, 9H), 5.94 (br s, 1H), 4.85 (d, *J* = 5.46 Hz, 2H), 2.28 (s, 3H); ^{13}C-NMR (125 MHz, $CDCl_3$) δ 181.34, 137.45, 135.85, 134.20, 131.78, 130.99, 128.72, 128.61, 127.66, 127.61, 127.55, 49.30, 17.81; HR-MS-ESI: m/z $[M+H]^+$ calculated for $C_{15}H_{16}N_2S$: 257.1107; found: 257.1121.

N-(3,4,5-trimethoxyphenyl)morpholine-4-carbothioamide (**3x**) [1] Light brown solid; 162-164 ºC; IR (KBr): 3165, 3109, 2972, 2904, 2852, 1603, 1531, 1464, 1423, 1317, 1230, 1128, 1030, 893, 725 cm^{-1}; ^{1}H-NMR (400 MHz, $CDCl_3$) δ 7.28 (br s, 1H), 6.42 (s, 2H), 3.89-3.68 (m, 17H); ^{13}C-NMR (100 MHz, $CDCl_3$) δ 183.54, 153.48, 135.75, 135.63, 101.30, 66.17, 60.97, 56.20, 49.46; HR-MS-ESI: m/z $[M+H]^+$ calculated for $C_{14}H_{20}N_2O_4S$: 313.1133; found: 313.1216.

N-(pyridin-2-yl)-N′-(3,4,5-trimethoxyphenyl)thiourea **(3y)** [2] Beige solid; 63-65 ºC; IR (KBr): 3560, 3406, 3238, 2837, 2133, 1595, 1236, 1348, 1114, 995, 821, 607 cm^{-1}; ^{1}H-NMR (200 MHz, $CDCl_3$) δ 8.22 (d, *J* = 4.52 Hz, 1H), 7.71 (t, *J* = 7.30 Hz, 1H), 7.18-6.89 (m, 4H), 3.89 (s, 6H), 3.87 (s, 3H); ^{13}C-NMR (50 MHz, $CDCl_3$) δ 178.43, 153.20 (2C), 139.51, 136.47, 134.25, 118.44, 113.15, 102.87 (2C), 60.99, 56.35; HR-MS-ESI: m/z $[M+Na]^+$ calculated for $C_{15}H_{17}N_3O_3S$: 342.0888; found: 342.0883.

N-(3,4-dimethoxyphenyl)-N′-(3,4,5-trimethoxyphenyl)thiourea (**3z**) [3] Purple solid; 181-183 ºC; IR (KBr): 3348, 3293, 2990, 2934, 2837, 1606, 1532, 1506, 1232, 1122, 1027, 815, 721 cm^{-1}; ^{1}H-NMR (400 MHz, $CDCl_3$) δ 8.05 (br s, 2H), 7.02-6.95 (m, 1H), 6.92-6.82 (m, 2H), 6.71-6.60 (m, 2H), 4.02-3.77 (m, 15H); ^{13}C-NMR (100 MHz, $CDCl_3$) δ 179.75, 153.58, 149.49, 148.25, 136.76, 133.02, 129.89, 118.09, 111.34, 109.88, 102.94, 60.92, 56.28, 56.10; HR-MS-ESI: m/z $[M+H]^+$ calculated for $C_{18}H_{22}N_2O_5S$: 379.1322; found: 379.1323.

N-(5-(trifluoromethyl)-1,3,4-thiadiazol-2-yl)-N′-(3,4,5-trimethoxyphenyl)thiourea (**3a'**) Pale yellow solid; 203-205 ºC; IR (KBr): 3440, 3335, 2935, 2839, 1598, 1552, 1509, 1378, 1233, 1132, 1039, 678 cm^{-1}; ^{1}H-NMR (400 MHz, DMSO-d_6) δ 10.62 (br s, 1H), 6.98 (s, 2H), 3.77 (s, 6H), 3.66 (s, 3H); ^{13}C-NMR (100 MHz, DMSO-d_6) δ 153.03, 135.42, 135.00, 121.65, 118.94,

101.71, 60.54, 56.39; HR-MS-ESI: m/z [M+Na]$^+$ calculated for $C_{13}H_{13}F_3N_4O_3S_2$: 417.0273; found: 417.0273.

1-butyl-3-(3,4,5-trimethoxyphenyl)thiourea (**3b′**) Pale yellow solid; 93-95 ºC; IR (KBr): 3317, 3180, 3014, 2962, 1603, 1547, 1504, 1450, 1417, 1313, 1228, 1082, 987, 837, 669 cm^{-1}; ^{1}H-NMR (200 MHz, $CDCl_3$) δ 8.21 (br s, 1H), 6.46 (s, 2H), 6.09 (br s, 1H), 3.86-3.80 (m, 9H), 3.62 (q, *J* = 7.05 Hz, 2H), 1.66-1.47 (m, 2H), 1.45-1.23 (m, 2H), 0.92 (t, *J* = 7.19 Hz, 3H); ^{13}C-NMR (50 MHz, $CDCl_3$) δ 180.63, 154.23, 137.17, 131.87, 103.10, 61.02, 56.35, 45.29, 31.17, 20.23, 13.85; HR-MS-ESI: m/z [M+H]$^+$ calculated for $C_{14}H_{22}N_2O_3S$: 299.1416; found: 299.1425.

N-phenethyl-4-(tetrahydrofuran-2-carbonyl)piperazine-1-carbothioamide (**5c**) Pale yellow solid; 111-113 °C; IR (KBr): 3305, 2922, 2858, 1637, 1631, 1533, 1442, 1298, 1230, 1188, 1056, 1007, 885, 742, 700, 489 cm^{-1}; ^{1}H-NMR (400 MHz, $CDCl_3$) δ 7.36-7.28 (m, 2H), 7.27- 7.19 (m, 3H), 5.63 (br s, 1H), 4.59-4.53 (m, 1H), 4.04-3.70 (m, 9H), 3.64-3.48 (m, 3H), 2.96 (t, *J* = 6.92 Hz, 2H), 2.33-2.23 (m, 1H), 2.09-1.85 (m, 3H); ^{13}C-NMR (100 MHz, $CDCl_3$) δ 182.21, 170.33, 138.76, 128.79, 126.73, 75.96, 69.15, 47.18, 46.96, 46.10, 44.38, 41.09, 35.06, 28.20, 25.76; HR-MS-ESI: m/z [M+Na]$^+$ calculated for $C_{18}H_{25}N_3O_2S$: 370.1552; found: 370.1560.

4-(benzo[d][1,3]dioxol-5-ylmethyl)-N-phenethylpiperazine-1-carbothioamide (**5f**) Beige solid; 152-154 °C; IR (KBr): 3324, 2917, 2802, 1542, 1486, 1448, 1338, 1236, 1180, 1031, 997, 925, 790, 701, 495 cm^{-1}; ^{1}H-NMR (500 MHz, $CDCl_3$) δ 7.33-7.27 (m, 2H), 7.25-7.17 (m, 3H), 6.83 (br s, 1H), 6.76-6.68 (m, 2H), 5.93 (s, 2H), 5.49 (br s, 1H), 3.92 (q, *J* = 6.26 Hz, 2H), 3.70 (t, *J* = 4.74 Hz, 4H), 3.40 (s, 2H), 2.94 (t, *J* = 6.79 Hz, 2H), 2.41 (t, *J* = 4.93 Hz, 4H); ^{13}C-NMR (125 MHz, $CDCl_3$) δ 181.87, 147.74, 146.81, 138.91, 131.40, 128.83, 128.74, 126.63, 122.21, 109.36, 107.94, 100.96, 62.38, 52.22, 47.27, 46.85, 35.16; HR-MS-ESI: m/z [M+H]$^+$ calculated for $C_{21}H_{25}N_3O_2S$: 384.1733; found: 384.1740.

4-benzhydryl-N-phenylpiperazine-1-carbothioamide (**5g**) [8] White solid; 216-218 °C; IR (KBr): 3440, 3193, 2798, 1594, 1513, 1450, 1324, 1226, 1035, 995, 929, 757, 692, 609 cm^{-1}; ^{1}H-NMR (500 MHz, $CDCl_3$) δ 7.39 (d, *J* = 7.57 Hz, 4H), 7.31-7.23 (m, 7H), 7.17 (t, *J* = 7.18 Hz, 2H), 7.10 (t, *J* = 7.35 Hz, 1H), 7.06 (d, *J* = 7.79 Hz, 2H), 4.23 (s, 1H), 3.78 (br s, 4H), 2.42 (t, *J* = 4.54 Hz, 4H); ^{13}C-NMR (125 MHz, $CDCl_3$) δ 183.12, 142.00, 140.12, 129.17, 128.66, 127.84, 127.25, 125.03, 122.70, 75.78, 51.28, 49.78; HR-MS-ESI: m/z [M+H]$^+$ calculated for $C_{24}H_{25}N_3S$: 388.1835; found: 388.1842.

4-benzhydryl-N-benzylpiperazine-1-carbothioamide (**5h**) White solid; 156-158 ºC; IR (KBr): 3315, 3021, 2800, 1598, 1535, 1450, 1330, 1220, 1143, 997, 879, 732, 694, 609, 453 cm^{-1}; ^{1}H-NMR (500 MHz, $CDCl_3$) δ 7.38 (d, *J* = 7.45 Hz, 4H), 7.33-7.23 (m, 9H), 7.17 (t, *J* = 7.26 Hz, 2H), 5.64 (br s, 1H), 4.82 (d, *J* = 4.77 Hz, 2H), 4.22 (s, 1H), 3.77 (t, *J* = 4.86 Hz, 4H), 2.41 (t, *J* = 4.86 Hz, 4H); ^{13}C-NMR (125 MHz, $CDCl_3$) δ 182.11, 142.00, 137.98, 128.82, 128.67, 128.09, 127.86, 127.76, 127.27, 75.76, 51.29, 50.32, 47.62; HR-MS-ESI: m/z [M+H]$^+$ calculated for $C_{25}H_{27}N_3S$: 402.1992; found: 402.1998.

4-benzhydryl-N-phenethylpiperazine-1-carbothioamide (**5i**) White solid; 138-140 °C; IR (KBr): 3345, 2927, 2805, 1540, 1407, 1338, 1238, 1143, 997, 881, 734, 705, 593, 499 cm^{-1}; ^{1}H-NMR (500 MHz, $CDCl_3$) δ 7.39 (d, *J* = 7.47 Hz, 4H), 7.30-7.23 (m, 6H), 7.22-7.15 (m, 5H), 5.43 (br s, 1H), 4.21 (s, 1H), 3.90 (q, *J* = 6.30 Hz, 2H), 3.68 (t, *J* = 4.91 Hz, 4H), 2.91 (t, *J* = 6.89 Hz, 2H), 2.38 (t, *J* = 5.02 Hz, 4H); ^{13}C-NMR (125 MHz, $CDCl_3$) δ 181.91, 142.04, 138.89, 128.81, 128.76, 128.66, 127.85, 127.25, 126.65, 75.79, 51.27, 47.33, 46.82, 35.16; HR-MS-ESI: m/z [M+H]$^+$ calculated for $C_{26}H_{29}N_3S$: 416.2148; found: 416.2154.

***N*-benzyl-4-(2-chlorophenyl)piperazine-1-carbothioamide** (**5k**) White solid; 110-112 ºC; IR (KBr): 3295, 2898, 2819, 1589, 1548, 1481, 1322, 1222, 1145, 1022, 968, 863, 738, 686, 549 cm^{-1}; ^{1}H-NMR (500 MHz, $CDCl_3$) δ 7.38-7.33 (m, 5H), 7.32-7.27 (m, 1H), 7.26-7.20 (m, 1H), 7.03-6.98 (m, 2H), 5.80 (br s, 1H), 4.89 (d, *J* = 4.77 Hz, 2H), 3.99 (t, *J* = 5.00 Hz, 4H), 3.08 (t, *J* = 5.00 Hz, 4H); ^{13}C-NMR (125 MHz, $CDCl_3$) δ 182.46, 148.34, 137.85, 130.76, 128.87, 128.18, 127.85, 127.77, 124.41, 120.50, 50.73, 50.47, 47.79; HR-MS-ESI: m/z $[M+Na]^+$ calculated for $C_{18}H_{20}ClN_3S$: 368.0889; found: 368.0958.

4-(2-chlorophenyl)-N-phenethylpiperazine-1-carbothioamide (**5l**) White solid; 111-113 °C; IR (KBr): 3301, 2939, 2809, 1587, 1527, 1479, 1346, 1228, 1124, 1008, 931, 862, 742, 701, 493 cm^{-1}; ^{1}H-NMR (500 MHz, $CDCl_3$) δ 7.36 (dd, *J* = 1.45 and 7.80 Hz, 1H), 7.32 (t, *J* = 7.46 Hz, 2H), 7.25-7.20 (m, 4H), 7.02-6.97 (m, 2H), 5.58 (t, *J* = 4.57 Hz, 1H), 3.96 (q, *J* = 6.80 Hz, 2H), 3.89 (t, *J* = 5.02 Hz, 4H), 3.04 (t, *J* = 5.07 Hz, 4H), 2.97 (t, *J* = 6.90 Hz, 2H); ^{13}C-NMR (125 MHz, $CDCl_3$) δ 182.30, 148.36, 138.88, 130.75, 128.88, 128.85, 128.78, 127.76, 126.69, 124.40, 120.49, 50.69, 47.53, 46.95, 35.16; HR-MS-ESI: m/z $[M+H]^+$ calculated for $C_{19}H_{22}ClN_3S$: 360.1226; found: 360.1295.

N-phenethyl-4-(2,3,4-trimethoxybenzyl)piperazine-1-carbothioamide (**5o**) White solid; 145-147 °C; IR (KBr): 3365, 2927, 2796, 1596, 1544, 1492, 1409, 1307, 1238, 1186, 1093, 998, 871, 754, 700, 499 cm^{-1}; ^{1}H-NMR (500 MHz, $CDCl_3$) δ 7.32-7.26 (m, 2H), 7.25-7.18 (m, 3H), 6.95 (d, *J* = 8.52 Hz, 1H), 6.63 (d, *J* = 8.52 Hz, 1H), 5.49 (br t, *J* = 4.91 Hz, 1H), 3.92 (q, *J* = 6.58 Hz, 2H), 3.87 (s, 6H), 3.85 (s, 3H), 3.70 (t, *J* = 5.05 Hz, 4H), 3.48 (s, 2H), 2.94 (t, *J* = 6.81 Hz, 2H), 2.46 (t, *J* = 5.05 Hz, 4H); ^{13}C-NMR (125 MHz, $CDCl_3$) δ 181.86, 153.14, 152.63, 142.33, 138.92, 128.83, 128.72, 126.61, 125.14, 123.24, 107.05, 61.21, 60.80, 56.23, 56.00, 52.20, 47.32, 46.85, 35.17; HR-MS-ESI: m/z $[M+H]^+$ calculated for $C_{23}H_{31}N_3O_3S$: 430.2152; found: 430.2158.

4-(1H-benzo[d]imidazol-2-yl)-N-phenylpiperazine-1-carbothioamide (**5p**) White solid; 229-231 °C; IR (KBr): 3401, 3171, 2927, 1626, 1563, 1531, 1427, 1225, 998, 742, 707 cm^{-1}; ^{1}H-NMR (200 MHz, DMSO-*d*$_6$) δ 9.46 (br s, 1H), 7.39-7.18 (m, 6H), 7.18-7.06 (m, 1H), 7.02-6.89 (m, 2H), 4.16-3.99 (m, 4H), 3.72-3.54 (m, 4H); ^{13}C-NMR (50 MHz, DMSO-*d*$_6$) δ 182.29; 156.26; 141.49; 130.46; 128.58; 126.48; 125.91; 124.99; 120.29; 47.89; 46.15; HR-MS-ESI: m/z $[M+H]^+$ calculated for $C_{18}H_{19}N_5S$: 338.1434; found: 338.1430.

4-(tetrahydrofuran-2-carbonyl)-N-(3,4,5-trimethoxyphenyl)piperazine-1-carbothioamide (**5r**) Pale yellow solid; 157-159 °C; IR (KBr): 3421, 3255, 2929, 1631, 1600, 1508, 1319, 1230, 1122, 1014, 831, 734 cm^{-1}; ^{1}H-NMR (400 MHz, $CDCl_3$) δ 7.57 (br s, 1H), 6.46 (s, 2H), 4.57 (t, *J* = 6.54 Hz, 1H), 4.11-4.01 (m, 1H), 3.97-3.78 (m, 15H), 3.77-3.67 (m, 1H), 3.66-3.50 (m, 2H), 2.35-2.24 (m, 1H), 2.10-1.85 (m, 3H); ^{13}C-NMR (100 MHz, $CDCl_3$) δ 183.27, 170.24, 153.35, 135.77, 135.66, 101.84, 76.01, 69.16, 60.95, 56.21, 48.95, 48.24, 44.50, 41.35, 28.18, 25.75; HR-MS-ESI: m/z $[M+Na]^+$ calculated for $C_{19}H_{27}N_3O_5S$: 432.1557; found: 432.1563.

4-(benzo[d][1,3]dioxol-5-ylmethyl)-N-(3,4,5-trimethoxyphenyl)piperazine-1-carbothioamide (**5s**) White solid; 156-158 °C; IR (KBr): 3353, 2937, 2804, 1596, 1500, 1438, 1321, 1228, 1122, 1035, 997, 921, 817, 721, 524 cm^{-1}; ^{1}H-NMR (400 MHz, $CDCl_3$) δ 7.28 (br s, 1H), 6.83 (s, 1H), 6.77-6.69 (m, 2H), 6.38 (s, 2H), 5.94 (s, 2H), 3.87-3.77 (m, 13H), 3.43 (s, 2H), 2.48 (t, *J* = 4.87 Hz, 4H); ^{13}C-NMR (100 MHz, $CDCl_3$) δ 182.93, 153.40, 147.76, 146.85, 135.94, 135.48, 131.22, 122.26, 109.37, 107.95, 101.02, 100.98, 62.38, 60.95, 56.16, 52.23, 49.42; HR-MS-ESI: m/z $[M+H]^+$ calculated for $C_{22}H_{27}N_3O_5S$: 446.1738; found: 446.1744.

4-benzhydryl-N-(3,4,5-trimethoxyphenyl)piperazine-1-carbothioamide (**5t**) White solid; 151-153 °C; IR (KBr): 3415, 2917, 2805, 1596, 1504, 1317, 1228, 1126, 995, 892, 823, 705, 609 cm^{-1}; ^{1}H-NMR (400 MHz, $CDCl_3$) δ 7.43-7.36 (m, 4H), 7.31-7.15 (m, 7H), 6.36 (s, 2H), 4.25 (s, 1H),

3.85-3.76 (m, 13H), 2.44 (t, *J* = 4.80 Hz, 4H); ^{13}C-NMR (100 MHz, $CDCl_3$) δ 183.00, 153.43, 141.82, 135.97, 135.49, 128.66, 127.87, 127.28, 100.88, 75.76, 60.95, 56.17, 51.29, 49.62; HR-MS-ESI: m/z [M+H]$^+$ calculated for $C_{27}H_{31}N_3O_3S$: 478.2153; found: 478.2159.

4-(2-chlorophenyl)-N-(3,4,5-trimethoxyphenyl)piperazine-1-carbothioamide (**5u**) White solide; 180-182 °C; IR (KBr): 3160, 2813, 2811, 1602, 1479, 1317, 1216, 1124, 1027, 931, 813, 730, 615, 439 cm^{-1}; ^{1}H-NMR (500 MHz, $CDCl_3$) δ 7.43 (br s, 1H), 7.38 (d, *J* = 7.75 Hz, 1H), 7.24 (t, *J* = 7.75 Hz, 1H), 7.02 (t, *J* = 7.75 Hz, 2H), 6.46 (s, 2H), 4.03 (t, *J* = 4.70 Hz, 4H), 3.83 (s, 9H), 3.11 (t, *J* = 4.70 Hz, 4H); ^{13}C-NMR (125 MHz, $CDCl_3$) δ 183.25, 153.43, 148.24, 135.89, 135.54, 130.78, 128.88, 127.79, 124.48, 120.48, 101.16, 60.98, 56.21, 50.75, 49.61; HR-MS-ESI: m/z [M+Na]$^+$ calculated for $C_{20}H_{24}ClN_3O_3S$: 444.1125; found: 444.1119.

4-(2,3,4-trimethoxybenzyl)-N-(3,4,5-trimethoxyphenyl)piperazine-1-carbothioamide (**5v**) Pale yellow solid; 116-118 °C; IR (KBr): 3330, 2937, 2828, 2124, 1601, 1507, 1464, 1318, 1231, 1126, 997, 731, 525 cm^{-1}; ^{1}H-NMR (400 MHz, $CDCl_3$) δ 7.27 (br s, 1H), 6.96 (d, *J* = 8.58 Hz, 1H), 6.64 (d, *J* = 8.58 Hz, 1H), 6.39 (s, 2H), 3.90-3.77 (m, 22H), 3.51 (s, 2H), 2.53 (t, *J* = 4.55 Hz, 4H); ^{13}C-NMR (100 MHz, $CDCl_3$) δ 182.89, 153.38, 153.19, 152.63, 142.32, 135.96, 135.48, 125.20, 122.99, 107.04, 101.07, 61.19, 60.93, 60.80, 56.20, 56.14, 56.00, 52.19, 49.45; HR-MS-ESI: m/z [M+H]$^+$ calculated for $C_{24}H_{33}N_3O_6S$: 492.2157; found: 492.2163.

Option 2 (These are the data as suggested by the reviewer, formatted in a structured table)

					HR-MS-ESI (m/z)		^{1}H NMR (δ ppm)	^{13}C NMR (δ ppm)
**Compound**	**Nomenclature**	**Molecular Formula**	**Melting Point (ºC)**	**IR (cm^{-1})**	**$[M+H]^+$ Calculated**	**$[M+H]^+$ Found**	**Chemical Shifts**	**Chemical Shifts**
**3a**	*N-phenylmorpholine-4-carbothioamide* [1]	$C_{11}H_{14}N_2OS$	130-132	3160, 3090, 3020, 2910, 2850, 1590, 1530, 1460, 1400, 1320, 1200, 935	223.0890	223.0900	(500 MHz, DMSO-d_6) 9.36 (br s, 1H), 7.30 (d, *J* = 4.22 Hz, 4H), 7.14-7.07 (m, 1H), 3.88 (t, *J* = 4.70 Hz, 4H), 3.65 (t, *J* = 4.70 Hz, 4H)	(125 MHz, DMSO-d_6) 182.25, 141.39, 128.49, 125.73, 124.85, 66.25, 48.90
**3b**	*N-benzylmorpholine-4-carbothioamide* [1]	$C_{12}H_{16}N_2OS$	97-99	3246, 3043, 2962, 2852, 1603, 1537, 1448, 1336, 1196, 962, 694	237.1044	237.1057	(400 MHz, $CDCl_3$) 7.45-7.15 (m, 5H), 5.75 (br s, 1H), 4.86 (d, *J* = 4.72 Hz, 2H), 3.86- 3.61 (m, 8H)	(100 MHz, $CDCl_3$) 182.76, 137.72, 128.86, 128.14, 127.87, 66.14, 50.38, 47.58
**3f**	*N-phenethyl-N′-(pyridin-2-yl)thiourea* [2]	$C_{14}H_{15}N_3S$	154-156	3480, 3223, 2998, 1604, 1146, 1090, 1040, 868, 778	258.1059	258.1060	(400 MHz, $CDCl_3$) 11.78 (br s, 1H), 9.29 (br s, 1H), 7.95 (d, J = 4.07 Hz, 1H), 7.62-7.55 (m, 1H), 7.35-7.20 (m, 5H), 6.92-6.84 (m, 2H), 4.04 (q, *J* = 6.81 Hz, 2H), 3.02 (t, *J* = 6.92 Hz, 2H)	(100 MHz, $CDCl_3$) 179.44, 153.42, 145.51, 138.98, 138.58, 128.99, 128.56, 126.55, 117.84, 112.09, 47.00, 35.00
**3i**	*1-benzyl-3-(3,4-dimethoxyphenyl)thiourea* [3]	$C_{16}H_{18}N_2O_2S$	149-151	3332, 3149, 2981, 1595, 1544, 1516, 1437, 1324, 1233, 1134, 1069, 1019, 960, 855, 789, 745, 700	303.1154	303.1162	(200 MHz, $CDCl_3$) 8.11 (br s, 1H), 7.34-7.23 (m, 5H), 6.87-6.69 (m, 3H), 6.23 (br s, 1H), 4.86 (d, *J* = 5.33 Hz, 2H), 3.84 (s, 3H), 3.78 (s, 3H)	(50 MHz, $CDCl_3$) 180.57, 150.11, 148.67, 137.59, 128.89, 128.74, 127.85, 127.79, 118.53, 111.98, 109.88, 56.20, 56.13, 49.66

**3j**	*1-(3,4-dimethoxyphenyl)-3-phenethylthiourea* [3]	$C_{17}H_{20}N_2O_2S$	184-186	3353, 3178, 3010, 2931, 2832, 1597, 1547, 1525, 1444, 1355, 1289, 1239, 1134, 1029, 842, 751, 702	317.1308	317.1318	(200 MHz, $CDCl_3$) 7.52 (br s, 1H), 7.27-7.06 (m, 5H), 6.81-6.73 (m, 1H), 6.65-6.54 (m, 2H), 5.85 (br s, 1H), 3.94-3.80 (m, 5H), 3.74 (s, 3H), 2.90 (t, *J* = 6.74 Hz, 2H)	(50 MHz, $CDCl_3$) 181.26, 150.09, 148.75, 138.59, 128.80, 128.38, 126.72, 118.75, 111.80, 109.91, 56.22, 56.14, 46.32, 34.92
**3k**	*1-benzyl-3-(3,5-dimethoxyphenyl)thiourea* [3]	$C_{16}H_{18}N_2O_2S$	124-126	3340, 3200, 3040, 3000, 2830, 1600, 1530, 1450, 1310, 1260, 1200, 1150, 1050, 924, 818, 698	303.1152	303.1162	(400 MHz, $CDCl_3$) 8.12 (br s, 1H), 7.37-7.24 (m, 5H), 6.51 (br s, 1H), 6.33 (s, 3H), 4.88 (d, J = 4.92 Hz, 2H), 3.76-3.65 (m, 6H)	(100 MHz, $CDCl_3$) 180.52, 161.89, 137.62, 137.28, 128.81, 127.79, 127.74, 102.87, 99.20, 55.50, 49.51
**3l**	*1-(3,5-dimethoxyphenyl)-3-phenethylthiourea* [3]	$C_{17}H_{20}N_2O_2S$	115-117	3350, 3159, 3005, 1686, 1610, 1587, 1525, 1443, 1315, 1223, 1190, 1153, 1005, 845, 661	317.1308	317.1319	(200 MHz, $CDCl_3$) 8.10 (br s, 1H), 7.32-7.09 (m, 5H), 6.35-6.29 (m, 1H), 6.28-6.15 (m, 3H), 3.88 (q, J = 6.40 Hz, 2H), 3.69 (s, 6H), 2.91 (t, J = 6.80 Hz, 2H)	(50 MHz, $CDCl_3$) 180,45, 161.96, 138.62, 137.69, 128.84 (2C), 126.72, 103.15, 99.31, 55.63, 46.54, 34.97
**3n**	*2-benzoyl-N-phenylhydrazinecarbothioamide* [4]	$C_{14}H_{13}N_3OS$	164-166	3302, 3221, 1641, 1606, 1556, 1497, 1462, 1361, 1231, 761, 715, 695	272.0852	272.0852	(500 MHz, DMSO-d_6) 10.65 (br s, 1H), 9.93 (br s, 1H), 9.82 (br s, 1H), 8.06 (d, J = 7.52 Hz, 2H), 7.68 (t, J = 7.36 Hz, 1H), 7.60 (t, J = 7.62 Hz, 2H), 7.54 (br s, 2H), 7.43 (t, J = 7.84 Hz, 2H), 7.26 (t, J = 7.36 Hz, 1H)	(125 MHz, DMSO-d_6) 181.62, 166.46, 139.75, 133.02, 132.31, 128.71, 128.37 (2C), 126.52, 125.49

**3q**	*N-benzyl-N′-(4-bromophenyl)thiourea* [5]	$C_{14}H_{13}BrN_2S$	139-141	3397, 3153, 2981, 1536, 1515, 1301, 1236, 1069, 825, 739	321.0056	321.0056	(400 MHz, $CDCl_3$) 8.36 (br s, 1H), 7.51 (d, *J* = 8.06 Hz, 2H), 7.41-7.24 (m, 5H), 7.11 (d, *J* = 8.06 Hz, 2H), 6.29 (br s, 1H), 4.86 (d, *J* = 4.55 Hz, 2H)	(100 MHz, $CDCl_3$) 180.72, 136.97, 135.20, 133.29, 128.90, 127.94, 127.74, 126.77, 120.71, 49.46
**3r**	*1-(5-methyl-1,3,4-thiadiazol-2-yl)-3-phenylthiourea*	$C_{10}H_{10}N_4S_2$	219-221	3325, 3252, 3194, 2671, 1653, 1598, 1552, 1498, 1361, 1323, 1237, 1059, 752, 652	251.0420	251.2516	(200 MHz, DMSO-d_6) 10.46 (br s, 1H), 7.66 (d, *J* = 8.20 Hz, 2H), 7.33 (t, *J* = 7.72 Hz, 2H), 7.11 (t, *J* = 7.25 Hz, 1H), 2.67 (s, 3H)	(50 MHz, DMSO-d_6) 183.57, 156.23, 140.00, 128.98, 124.81, 123.23, 119.45, 15.72
**3s**	*N-benzyl-N′-(5-ethyl-1,3,4-thiadiazol-2-yl)thiourea*	$C_{12}H_{14}N_4S_2$	163-165	3341, 2973, 1632, 1533, 1454, 1379, 1348, 1276, 782, 699, 656	279.0733	279.0726	(500 MHz, DMSO-d_6) 7.54-749 (m, 2H), 7.48-7.36 (m, 3H), 4.99 (d, *J* = 5.15 Hz, 2H), 2.93 (q, *J* = 7.44 Hz, 2H), 1.36 (t, *J* = 7.44 Hz, 3H)	(125 MHz, DMSO-d_6) 178.86, 164.50, 164.15, 136.79, 128.65, 128.19, 127.74, 49.37, 23.22, 13.55
**3t**	*N-butyl-N′-phenylthiourea* [6]	$C_{11}H_{16}N_2S$	63-65	3295, 3171, 3003, 2950, 2925, 2857, 1596, 1551, 1534, 1321, 1242, 1066, 932, 693, 605	209.1097	209.1107	(200 MHz, $CDCl_3$) 8.21 (br s, 1H), 7.51-7.36 (m, 2H), 7.35-7.15 (m, 3H), 6.05 (br s, 1H), 3.62 (q, *J* = 6.47 Hz, 2H), 1.55 (quint, *J* = 7.17 Hz, 2H), 1.44-1.20 (m, 2H), 0.91 (t, *J* = 7.14 Hz, 3H)	(50 MHz, $CDCl_3$) 180.57, 136.35, 130.27, 127.27, 125.28, 45.36, 31.12, 20.15, 13.83
**3u**	*N-benzyl-N′-(o-tolyl)thiourea* [7]	$C_{15}H_{16}N_2S$	136-138	3500, 3169, 2968, 1618, 1539, 1249, 970, 743, 642	257.1107	257.1121	(500 MHz, $CDCl_3$) 7.84 (br s, 1H), 7.33-7.18 (m, 9H), 5.94 (br s, 1H), 4.85 (d, *J* = 5.46 Hz, 2H), 2.28 (s, 3H)	(125 MHz, $CDCl_3$) 181.34, 137.45, 135.85, 134.20, 131.78, 130.99, 128.72, 128.61, 127.66, 127.61, 127.55, 49.30, 17.81

**3x**	*N-(3,4,5-trimethoxyphenyl)morpholine-4-carbothioamide* [1]	$C_{14}H_{20}N_2O_4S$	162-164	3165, 3109, 2972, 2904, 2852, 1603, 1531, 1464, 1423, 1317, 1230, 1128, 1030, 893, 725	313.1133	313.1216	(400 MHz, $CDCl_3$) 7.28 (br s, 1H), 6.42 (s, 2H), 3.89-3.68 (m, 17H)	(100 MHz, $CDCl_3$) 183.54, 153.48, 135.75, 135.63, 101.30, 66.17, 60.97, 56.20, 49.46
**3y**	*N-(pyridin-2-yl)-N′-(3,4,5-trimethoxyphenyl)thiourea* [2]	$C_{15}H_{17}N_3O_3S$	63-65	3560, 3406, 3238, 2837, 2133, 1595, 1236, 1348, 1114, 995, 821, 607	342.0888	342.0883	(200 MHz, $CDCl_3$) 8.22 (d, *J* = 4.52 Hz, 1H), 7.71 (t, *J* = 7.30 Hz, 1H), 7.18-6.89 (m, 4H), 3.89 (s, 6H), 3.87 (s, 3H)	(50 MHz, $CDCl_3$) 178.43, 153.20 (2C), 139.51, 136.47, 134.25, 118.44, 113.15, 102.87 (2C), 60.99, 56.35
**3z**	*N-(3,4-dimethoxyphenyl)-N′-(3,4,5-trimethoxyphenyl)thiourea* [3]	$C_{18}H_{22}N_2O_5S$	181-183	3348, 3293, 2990, 2934, 2837, 1606, 1532, 1506, 1232, 1122, 1027, 815, 721	379.1322	379.1323	(400 MHz, $CDCl_3$) 8.05 (br s, 2H), 7.02-6.95 (m, 1H), 6.92-6.82 (m, 2H), 6.71-6.60 (m, 2H), 4.02-3.77 (m, 15H)	(100 MHz, $CDCl_3$) 179.75, 153.58, 149.49, 148.25, 136.76, 133.02, 129.89, 118.09, 111.34, 109.88, 102.94, 60.92, 56.28, 56.10
**3a'**	*N-(5-(trifluoromethyl)-1,3,4-thiadiazol-2-yl)-N′-(3,4,5-trimethoxyphenyl)thiourea*	$C_{13}H_{13}F_3N_4O_3S_2$	203-205	3440, 3335, 2935, 2839, 1598, 1552, 1509, 1378, 1233, 1132, 1039, 678	417.0273	417.0270	(400 MHz, DMSO-d_6) 10.62 (br s, 1H), 6.98 (s, 2H), 3.77 (s, 6H), 3.66 (s, 3H)	(100 MHz, DMSO-d_6) 153.03, 135.42, 135.00, 121.65, 118.94, 101.71, 60.54, 56.39
**3b′**	*1-butyl-3-(3,4,5-trimethoxyphenyl)thiourea*	$C_{14}H_{22}N_2O_3S$	93-95	3317, 3180, 3014, 2962, 1603, 1547, 1504, 1450, 1417, 1313, 1228, 1082, 987, 837, 669	299.1416	299.1425	(200 MHz, $CDCl_3$) 8.21 (br s, 1H), 6.46 (s, 2H), 6.09 (br s, 1H), 3.86-3.80 (m, 9H), 3.62 (q, *J* = 7.05 Hz, 2H), 1.66-1.47 (m, 2H), 1.45-1.23 (m, 2H), 0.92 (t, *J* = 7.19 Hz, 3H)	(50 MHz, $CDCl_3$) 180.63, 154.23, 137.17, 131.87, 103.10, 61.02, 56.35, 45.29, 31.17, 20.23, 13.85

**5c**	*N-phenethyl-4-(tetrahydrofuran-2-carbonyl)piperazine-1-carbothioamide*	$C_{18}H_{25}N_3O_2S$	111-113	3305, 2922, 2858, 1637, 1631, 1533, 1442, 1298, 1230, 1188, 1056, 1007, 885, 742, 700, 489	370.1552	370.156	(400 MHz, $CDCl_3$) 7.36-7.28 (m, 2H), 7.27- 7.19 (m, 3H), 5.63 (br s, 1H), 4.59-4.53 (m, 1H), 4.04-3.70 (m, 9H), 3.64-3.48 (m, 3H), 2.96 (t, *J* = 6.92 Hz, 2H), 2.33-2.23 (m, 1H), 2.09-1.85 (m, 3H)	(100 MHz, $CDCl_3$) 182.21, 170.33, 138.76, 128.79, 126.73, 75.96, 69.15, 47.18, 46.96, 46.10, 44.38, 41.09, 35.06, 28.20, 25.76
**5f**	*4-(benzo[d][1,3]dioxol-5-ylmethyl)-N-phenethylpiperazine-1-carbothioamide*	$C_{21}H_{25}N_3O_2S$	152-154	3324, 2917, 2802, 1542, 1486, 1448, 1338, 1236, 1180, 1031, 997, 925, 790, 701, 495	384.1733	384.174	(500 MHz, $CDCl_3$) 7.33-7.27 (m, 2H), 7.25-7.17 (m, 3H), 6.83 (br s, 1H), 6.76-6.68 (m, 2H), 5.93 (s, 2H), 5.49 (br s, 1H), 3.92 (q, *J* = 6.26 Hz, 2H), 3.70 (t, *J* = 4.74 Hz, 4H), 3.40 (s, 2H), 2.94 (t, *J* = 6.79 Hz, 2H), 2.41 (t, *J* = 4.93 Hz, 4H)	(125 MHz, $CDCl_3$) 181.87, 147.74, 146.81, 138.91, 131.40, 128.83, 128.74, 126.63, 122.21, 109.36, 107.94, 100.96, 62.38, 52.22, 47.27, 46.85, 35.16
**5g**	*4-benzhydryl-N-phenylpiperazine-1-carbothioamide* [8]	$C_{24}H_{25}N_3S$	216-218	3440, 3193, 2798, 1594, 1513, 1450, 1324, 1226, 1035, 995, 929, 757, 692, 609	388.1835	388.1842	(500 MHz, $CDCl_3$) 7.39 (d, *J* = 7.57 Hz, 4H), 7.31-7.23 (m, 7H), 7.17 (t, *J* = 7.18 Hz, 2H), 7.10 (t, *J* = 7.35 Hz, 1H), 7.06 (d, *J* = 7.79 Hz, 2H), 4.23 (s, 1H), 3.78 (br s, 4H), 2.42 (t, *J* = 4.54 Hz, 4H)	(125 MHz, $CDCl_3$) 183.12, 142.00, 140.12, 129.17, 128.66, 127.84, 127.25, 125.03, 122.70, 75.78, 51.28, 49.78
**5h**	*4-benzhydryl-N-benzylpiperazine-1-carbothioamide*	$C_{25}H_{27}N_3S$	156-158	3315, 3021, 2800, 1598, 1535, 1450, 1330, 1220, 1143, 997, 879, 732, 694, 609, 453	402.1992	402.1998	(500 MHz, $CDCl_3$) 7.38 (d, *J* = 7.45 Hz, 4H), 7.33-7.23 (m, 9H), 7.17 (t, *J* = 7.26 Hz, 2H), 5.64 (br s, 1H), 4.82 (d, *J* = 4.77 Hz, 2H), 4.22 (s, 1H), 3.77 (t, *J* = 4.86	(125 MHz, $CDCl_3$) 182.11, 142.00, 137.98, 128.82, 128.67, 128.09, 127.86, 127.76, 127.27, 75.76, 51.29, 50.32, 47.62

							Hz, 4H), 2.41 (t, *J* = 4.86 Hz, 4H)	
**5i**	*4-benzhydryl-N-phenethylpiperazine-1-carbothioamide*	$C_{26}H_{29}N_3S$	138-140	3345, 2927, 2805, 1540, 1407, 1338, 1238, 1143, 997, 881, 734, 705, 593, 499	416.2148	416.2154	(500 MHz, $CDCl_3$) 7.39 (d, *J* = 7.47 Hz, 4H), 7.30-7.23 (m, 6H), 7.22-7.15 (m, 5H), 5.43 (br s, 1H), 4.21 (s, 1H), 3.90 (q, *J* = 6.30 Hz, 2H), 3.68 (t, *J* = 4.91 Hz, 4H), 2.91 (t, *J* = 6.89 Hz, 2H), 2.38 (t, *J* = 5.02 Hz, 4H)	(125 MHz, $CDCl_3$) 181.91, 142.04, 138.89, 128.81, 128.76, 128.66, 127.85, 127.25, 126.65, 75.79, 51.27, 47.33, 46.82, 35.16
**5k**	*N-benzyl-4-(2-chlorophenyl)piperazine-1-carbothioamide*	$C_{18}H_{20}ClN_3S$	110-112	3295, 2898, 2819, 1589, 1548, 1481, 1322, 1222, 1145, 1022, 968, 863, 738, 686, 549	368.0889	368.0958	(500 MHz, $CDCl_3$) 7.38-7.33 (m, 5H), 7.32-7.27 (m, 1H), 7.26-7.20 (m, 1H), 7.03-6.98 (m, 2H), 5.80 (br s, 1H), 4.89 (d, *J* = 4.77 Hz, 2H), 3.99 (t, *J* = 5.00 Hz, 4H), 3.08 (t, *J* = 5.00 Hz, 4H)	(125 MHz, $CDCl_3$) 182.46, 148.34, 137.85, 130.76, 128.87, 128.18, 127.85, 127.77, 124.41, 120.50, 50.73, 50.47, 47.79

**5l**	*4-(2-chlorophenyl)-N-phenethylpiperazine-1-carbothioamide*	$C_{19}H_{22}ClN_3S$	111-113	3301, 2939, 2809, 1587, 1527, 1479, 1346, 1228, 1124, 1008, 931, 862, 742, 701, 493	360.1226	360.1295	(500 MHz, $CDCl_3$) 7.36 (dd, *J* = 1.45 and 7.80 Hz, 1H), 7.32 (t, *J* = 7.46 Hz, 2H), 7.25-7.20 (m, 4H), 7.02-6.97 (m, 2H), 5.58 (t, *J* = 4.57 Hz, 1H), 3.96 (q, *J* = 6.80 Hz, 2H), 3.89 (t, *J* = 5.02 Hz, 4H), 3.04 (t, *J* = 5.07 Hz, 4H), 2.97 (t, *J* = 6.90 Hz, 2H)	(125 MHz, $CDCl_3$) 182.30, 148.36, 138.88, 130.75, 128.88, 128.85, 128.78, 127.76, 126.69, 124.40, 120.49, 50.69, 47.53, 46.95, 35.16
**5o**	*N-phenethyl-4-(2,3,4-trimethoxybenzyl)piperazine-1-carbothioamide*	$C_{23}H_{31}N_3O_3S$	145-147	3365, 2927, 2796, 1596, 1544, 1492, 1409, 1307, 1238, 1186, 1093, 998, 871, 754, 700, 499	430.2152	430.2158	(500 MHz, $CDCl_3$) 7.32-7.26 (m, 2H), 7.25-7.18 (m, 3H), 6.95 (d, *J* = 8.52 Hz, 1H), 6.63 (d, *J* = 8.52 Hz, 1H), 5.49 (br t, *J* = 4.91 Hz, 1H), 3.92 (q, *J* = 6.58 Hz, 2H), 3.87 (s, 6H), 3.85 (s, 3H), 3.70 (t, *J* = 5.05 Hz, 4H), 3.48 (s, 2H), 2.94 (t, *J* = 6.81 Hz, 2H), 2.46 (t, *J* = 5.05 Hz, 4H)	(125 MHz, $CDCl_3$) 181.86, 153.14, 152.63, 142.33, 138.92, 128.83, 128.72, 126.61, 125.14, 123.24, 107.05, 61.21, 60.80, 56.23, 56.00, 52.20, 47.32, 46.85, 35.17
**5p**	*4-(1H-benzo[d]imidazol-2-yl)-N-phenylpiperazine-1-carbothioamide*	$C_{18}H_{19}N_5S$	229-231	3401, 3171, 2927, 1626, 1563, 1531, 1427, 1225, 998, 742, 707	338.1434	338.143	(200 MHz, DMSO-d_6) 9.46 (br s, 1H), 7.39-7.18 (m, 6H), 7.18-7.06 (m, 1H), 7.02-6.89 (m, 2H), 4.16-3.99 (m, 4H), 3.72-3.54 (m, 4H)	(50 MHz, DMSO-d_6) 182.29; 156.26; 141.49; 130.46; 128.58; 126.48; 125.91; 124.99; 120.29; 47.89; 46.15

**5r**	*4-(tetrahydrofuran-2-carbonyl)-N-(3,4,5-trimethoxyphenyl)piperazine-1-carbothioamide*	$C_{19}H_{27}N_3O_5S$	157-159	3421, 3255, 2929, 1631, 1600, 1508, 1319, 1230, 1122, 1014, 831, 734	432.1557	432.1563	(400 MHz, $CDCl_3$) 7.57 (br s, 1H), 6.46 (s, 2H), 4.57 (t, *J* = 6.54 Hz, 1H), 4.11-4.01 (m, 1H), 3.97-3.78 (m, 15H), 3.77-3.67 (m, 1H), 3.66-3.50 (m, 2H), 2.35-2.24 (m, 1H), 2.10-1.85 (m, 3H)	(100 MHz, $CDCl_3$) 183.27, 170.24, 153.35, 135.77, 135.66, 101.84, 76.01, 69.16, 60.95, 56.21, 48.95, 48.24, 44.50, 41.35, 28.18, 25.75
**5s**	*4-(benzo[d][1,3]dioxol-5-ylmethyl)-N-(3,4,5-trimethoxyphenyl)piperazine-1-carbothioamide*	$C_{22}H_{27}N_3O_5S$	156-158	3353, 2937, 2804, 1596, 1500, 1438, 1321, 1228, 1122, 1035, 997, 921, 817, 721, 524	446.1738	446.1744	(400 MHz, $CDCl_3$) 7.28 (br s, 1H), 6.83 (s, 1H), 6.77-6.69 (m, 2H), 6.38 (s, 2H), 5.94 (s, 2H), 3.87-3.77 (m, 13H), 3.43 (s, 2H), 2.48 (t, *J* = 4.87 Hz, 4H)	(100 MHz, $CDCl_3$) 182.93, 153.40, 147.76, 146.85, 135.94, 135.48, 131.22, 122.26, 109.37, 107.95, 101.02, 100.98, 62.38, 60.95, 56.16, 52.23, 49.42
**5t**	*4-benzhydryl-N-(3,4,5-trimethoxyphenyl)piperazine-1-carbothioamide*	$C_{27}H_{31}N_3O_3S$	151-153	3415, 2917, 2805, 1596, 1504, 1317, 1228, 1126, 995, 892, 823, 705, 609	478.2153	478.2159	(400 MHz, $CDCl_3$) 7.43-7.36 (m, 4H), 7.31-7.15 (m, 7H), 6.36 (s, 2H), 4.25 (s, 1H), 3.85-3.76 (m, 13H), 2.44 (t, *J* = 4.80 Hz, 4H)	(100 MHz, $CDCl_3$) 183.00, 153.43, 141.82, 135.97, 135.49, 128.66, 127.87, 127.28, 100.88, 75.76, 60.95, 56.17, 51.29, 49.62
**5u**	*4-(2-chlorophenyl)-N-(3,4,5-trimethoxyphenyl)piperazine-1-carbothioamide*	$C_{20}H_{24}ClN_3O_3S$	180-182	3160, 2813, 2811, 1602, 1479, 1317, 1216, 1124, 1027, 931, 813, 730, 615, 439	444.1125	444.1119	(500 MHz, $CDCl_3$) 7.43 (br s, 1H), 7.38 (d, J = 7.75 Hz, 1H), 7.24 (t, J = 7.75 Hz, 1H), 7.02 (t, J = 7.75 Hz, 2H), 6.46 (s, 2H), 4.03 (t, J = 4.70 Hz, 4H), 3.83 (s, 9H), 3.11 (t, J = 4.70 Hz, 4H)	(125 MHz, $CDCl_3$) 183.25, 153.43, 148.24, 135.89, 135.54, 130.78, 128.88, 127.79, 124.48, 120.48, 101.16, 60.98, 56.21, 50.75, 49.61

**5v**	*4-(2,3,4-trimethoxybenzyl)-N-(3,4,5-trimethoxyphenyl)piperazine-1-carbothioamide*	$C_{24}H_{33}N_3O_6S$	116-118	3330, 2937, 2828, 2124, 1601, 1507, 1464, 1318, 1231, 1126, 997, 731, 525	492.2157	492.2163	(400 MHz, $CDCl_3$) 7.27 (br s, 1H), 6.96 (d, *J* = 8.58 Hz, 1H), 6.64 (d, *J* = 8.58 Hz, 1H), 6.39 (s, 2H), 3.90-3.77 (m, 22H), 3.51 (s, 2H), 2.53 (t, *J* = 4.55 Hz, 4H)	(100 MHz, $CDCl_3$) 182.89, 153.38, 153.19, 152.63, 142.32, 135.96, 135.48, 125.20, 122.99, 107.04, 101.07, 61.19, 60.93, 60.80, 56.20, 56.14, 56.00, 52.19, 49.45

2 – Copies of ^{1}H-NMR, ^{13}C-NMR, FT-IR and HR-MS Spectra for Thiourea Derivatives

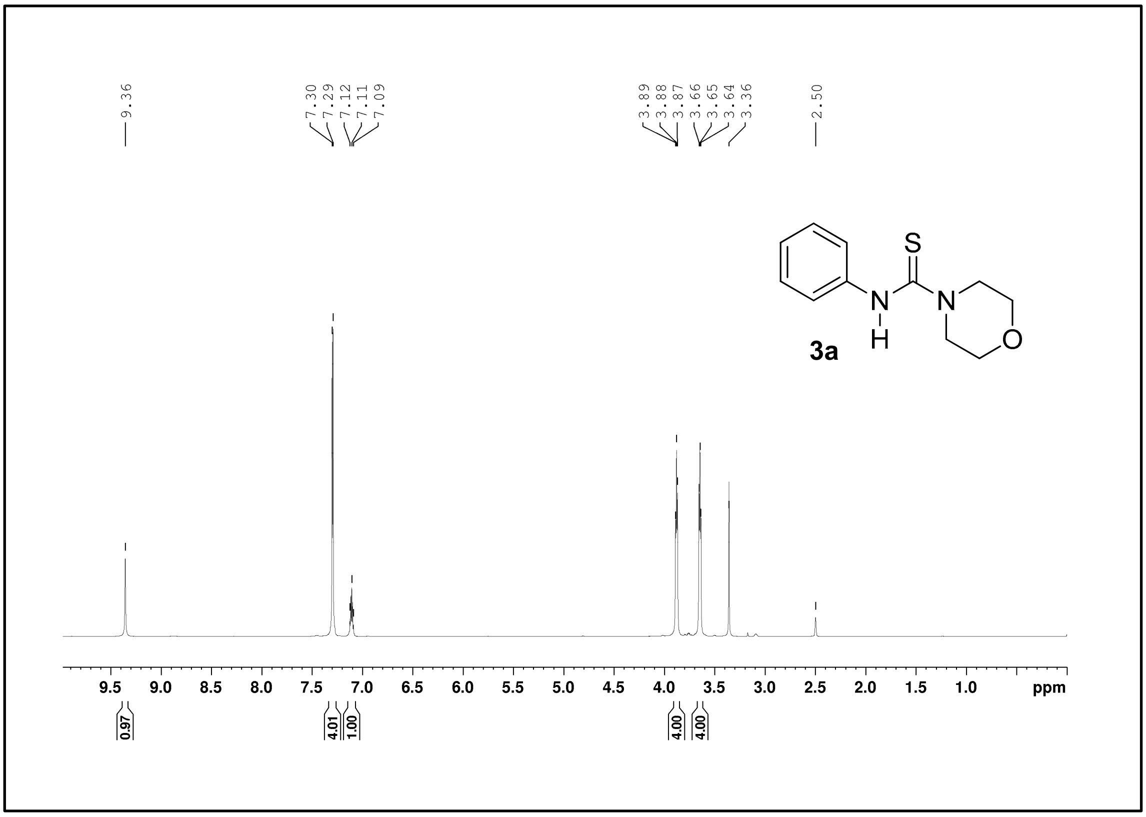

(A)

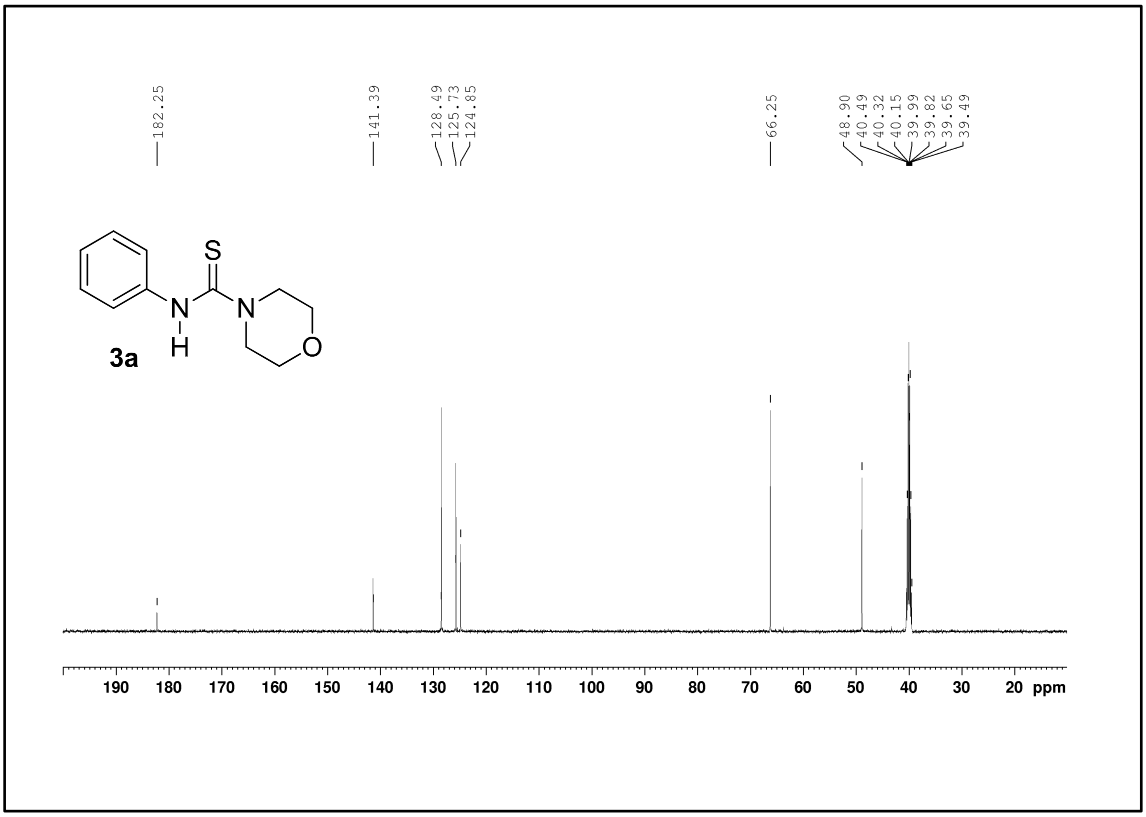

(B)

Figure S1. ^{1}H-NMR spectrum (A) and ^{13}C-NMR spectrum (B) of thiourea **3a**.

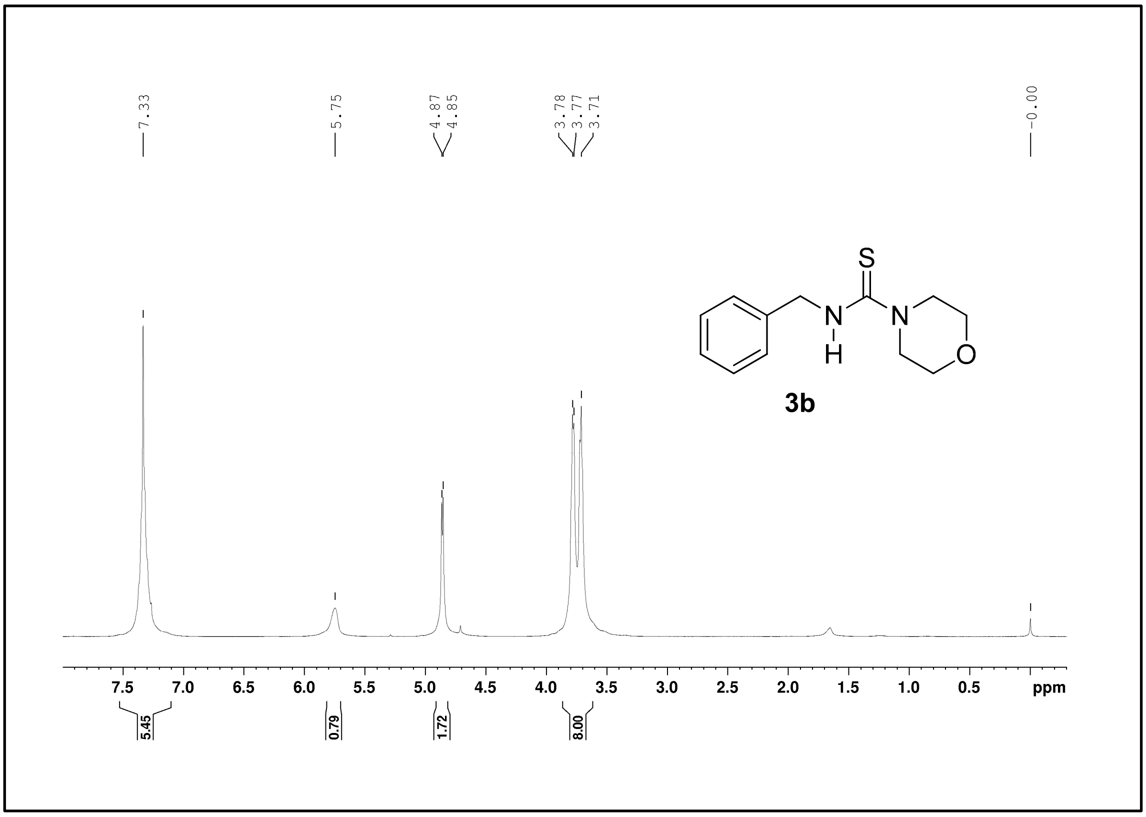

(A)

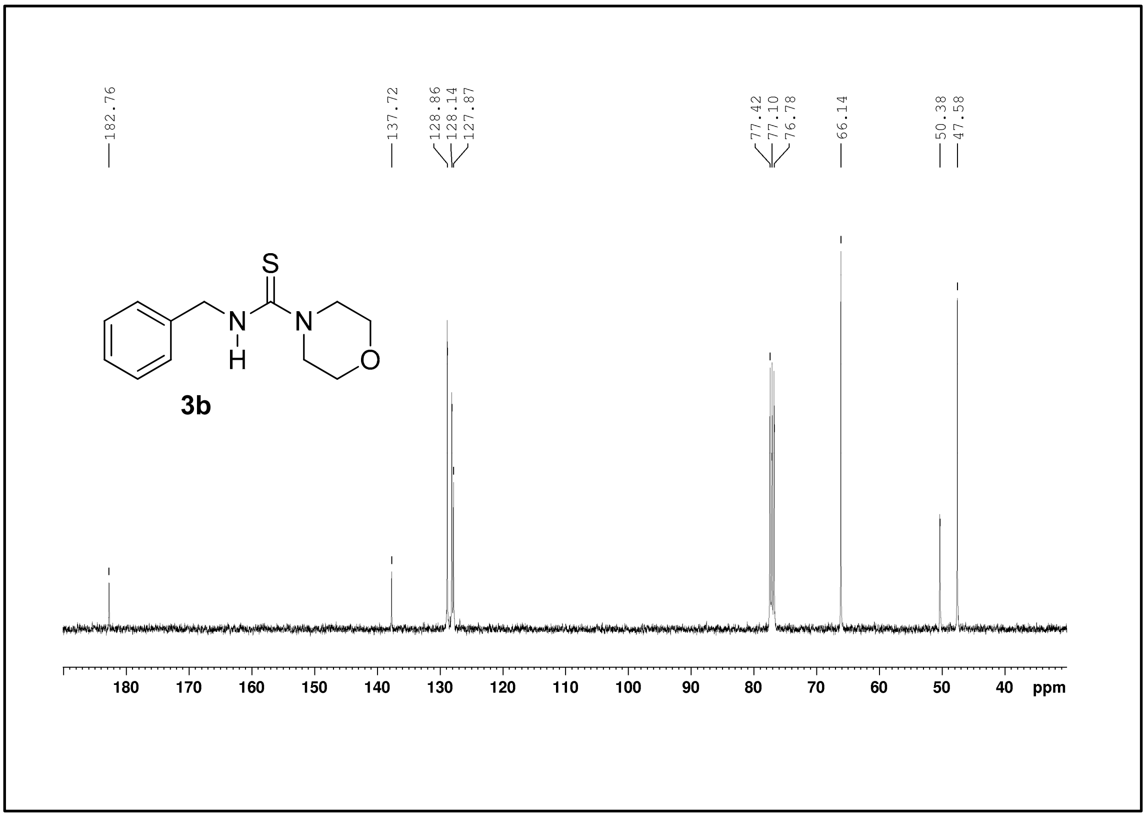

(B)

Figure S2. ^{1}H-NMR spectrum (A) and ^{13}C-NMR spectrum (B) of thiourea **3b**.

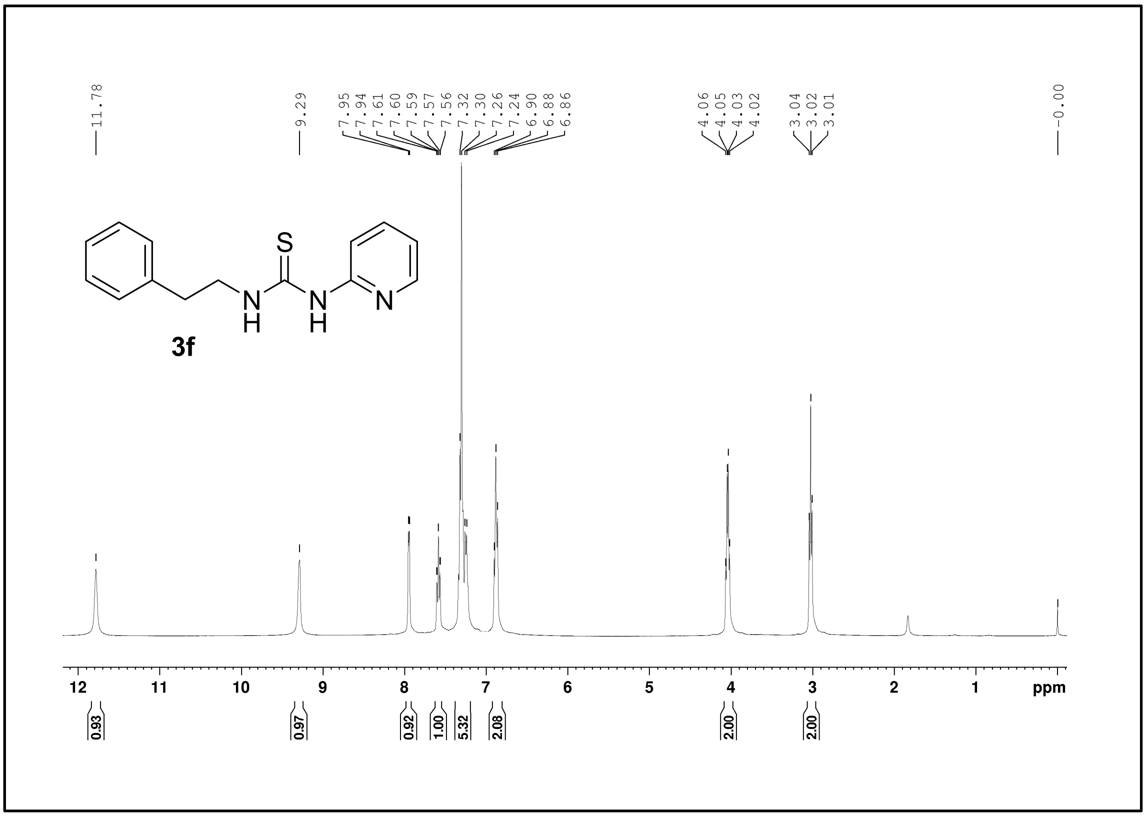

(A)

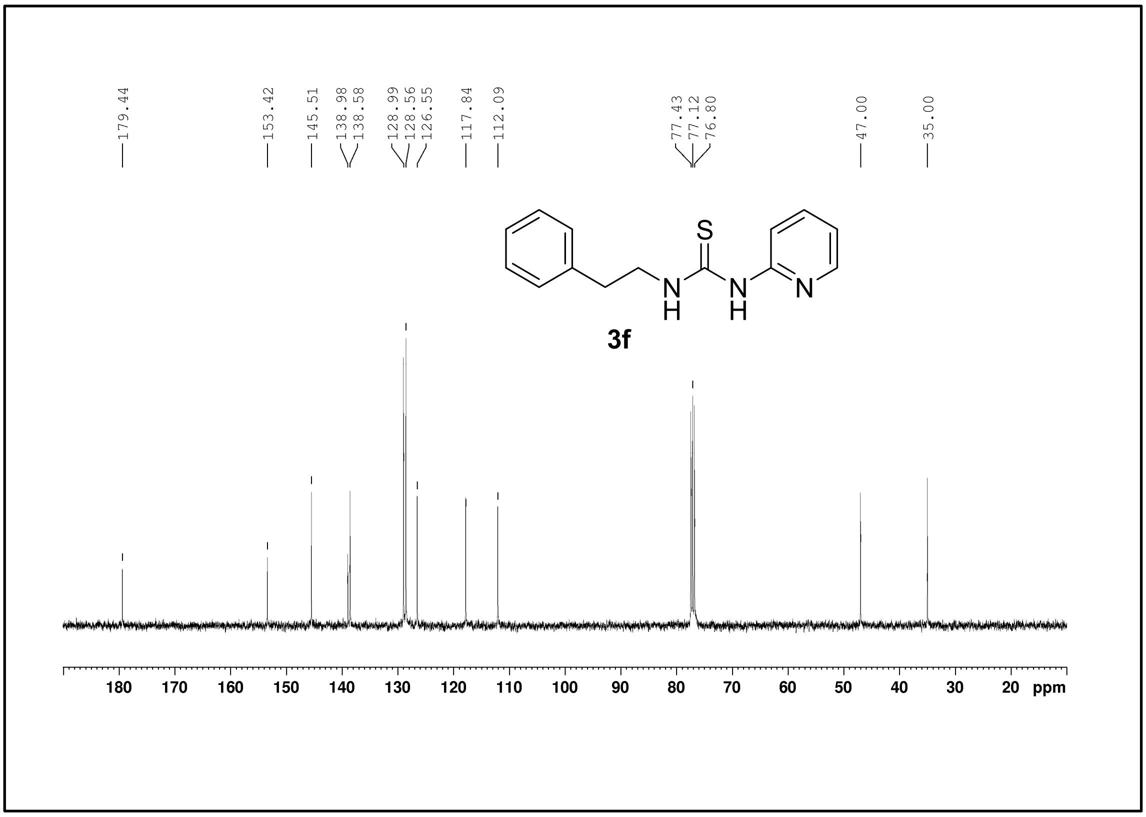

(B)

Figure S3. ^{1}H-NMR spectrum (A) and ^{13}C-NMR spectrum (B) of thiourea **3f**.

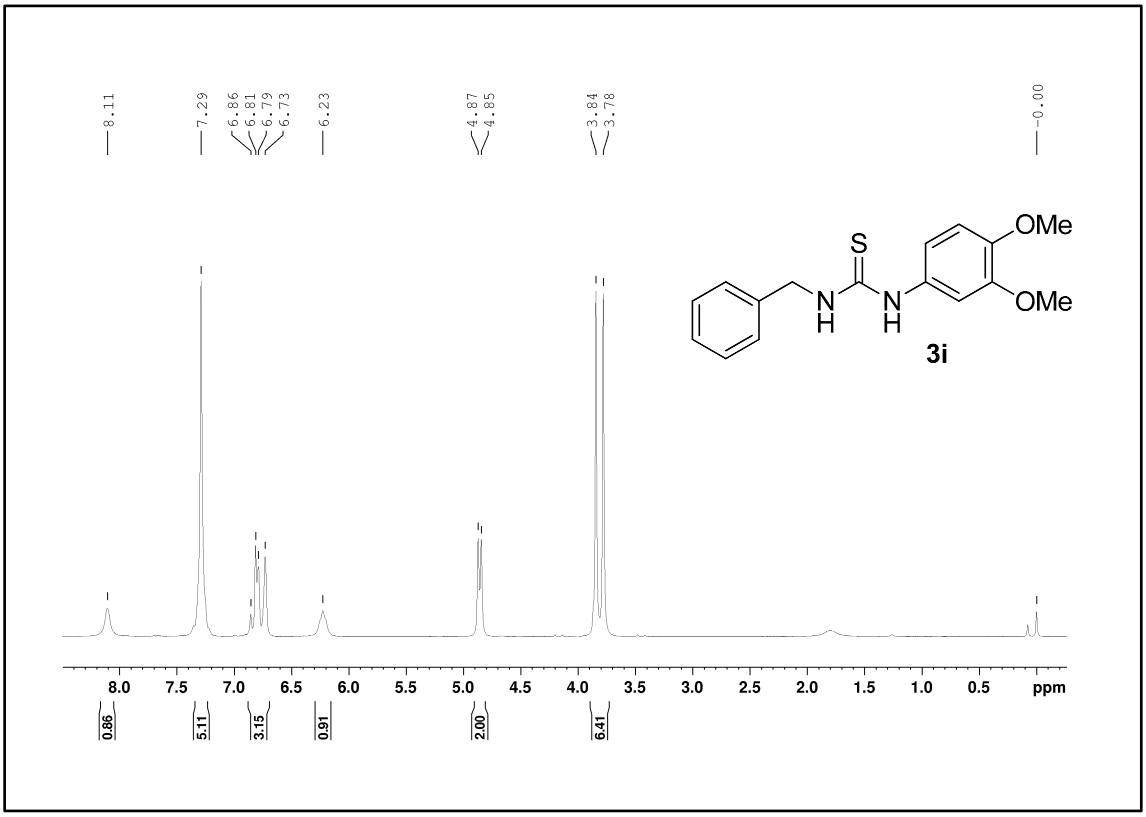

(A)

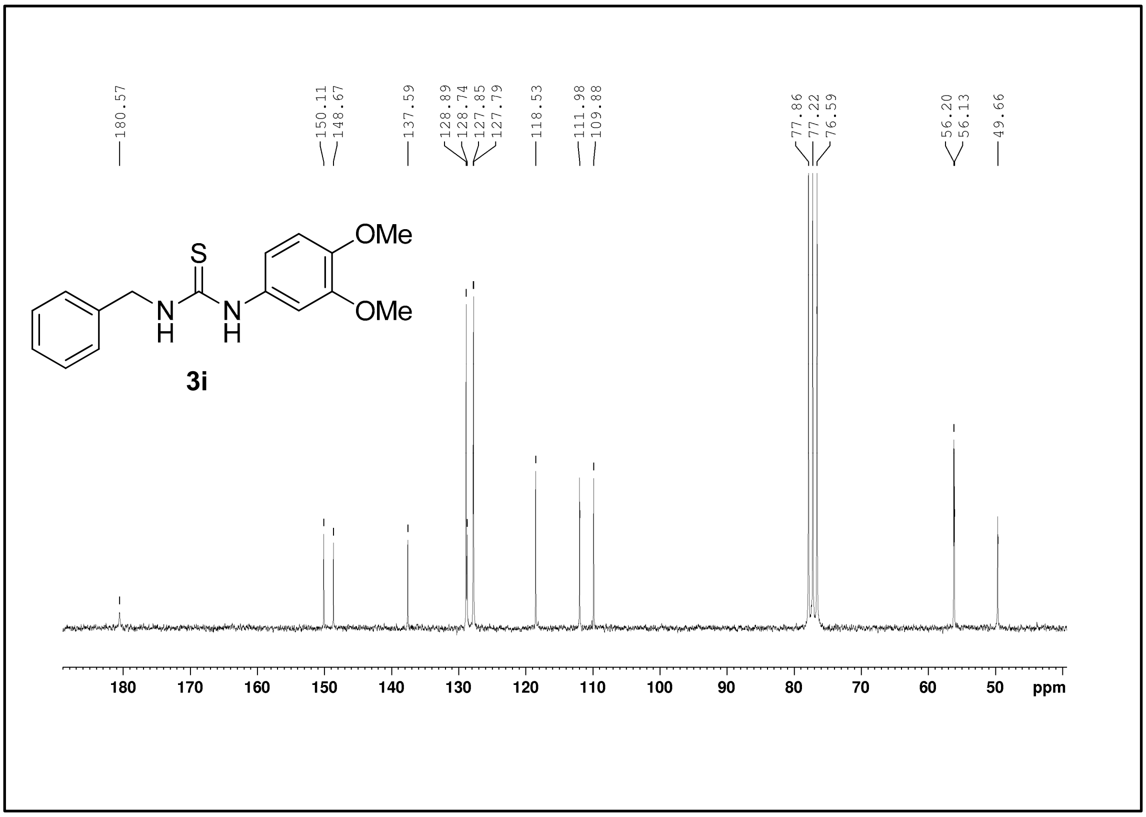

(B)

Figure S4. ^{1}H-NMR spectrum (A) and ^{13}C-NMR spectrum (B) of thiourea **3i**.

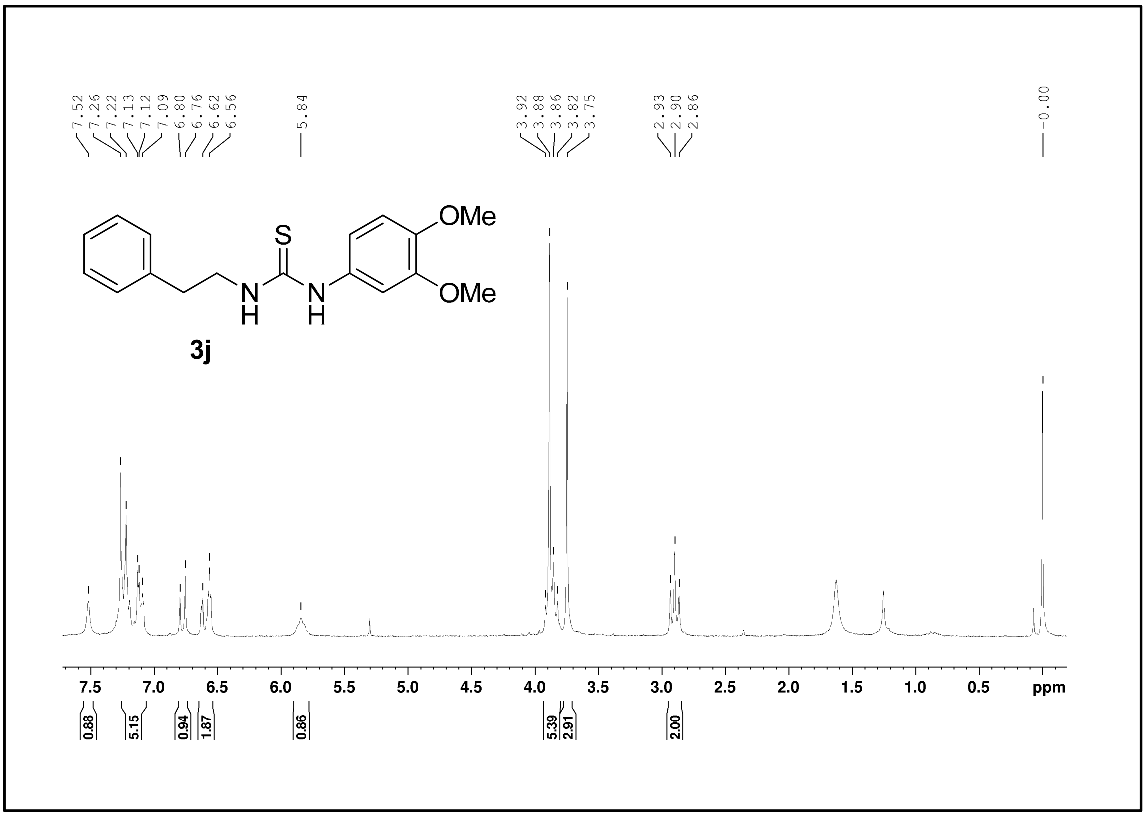

(A)

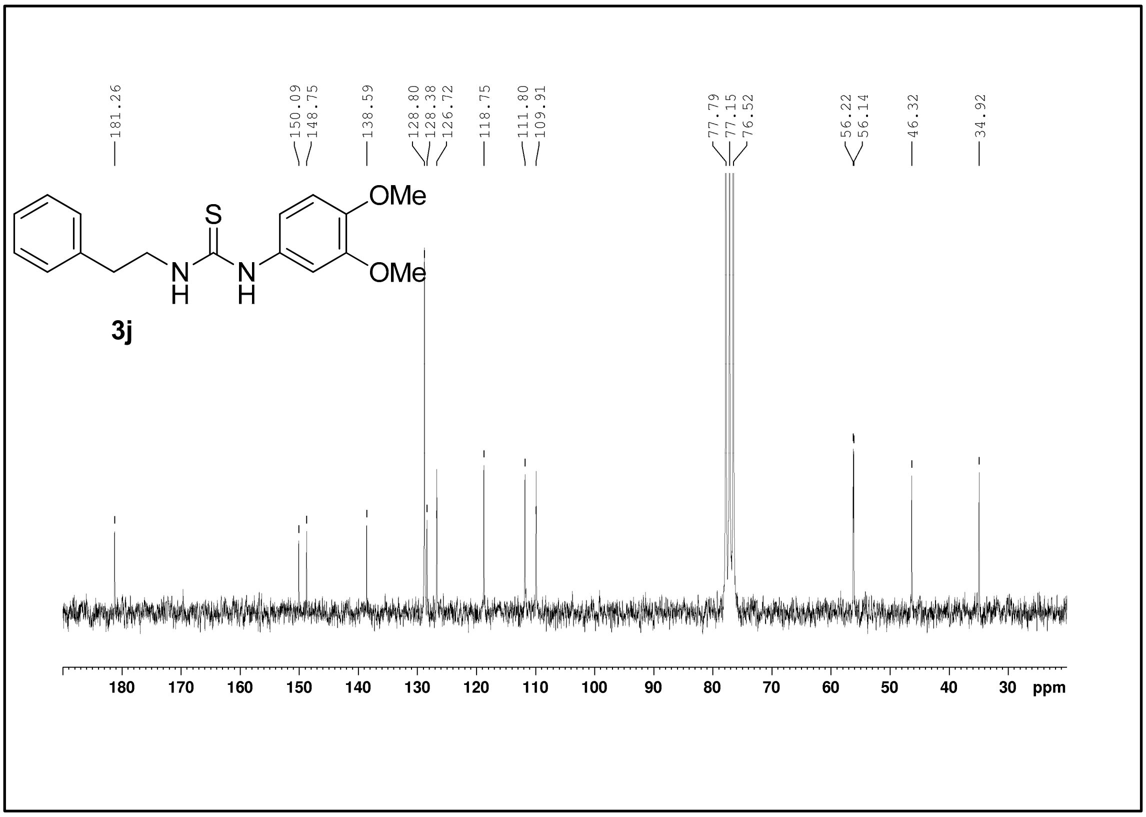

(B)

Figure S5. ^{1}H-NMR spectrum (A) and ^{13}C-NMR spectrum (B) of thiourea **3j**.

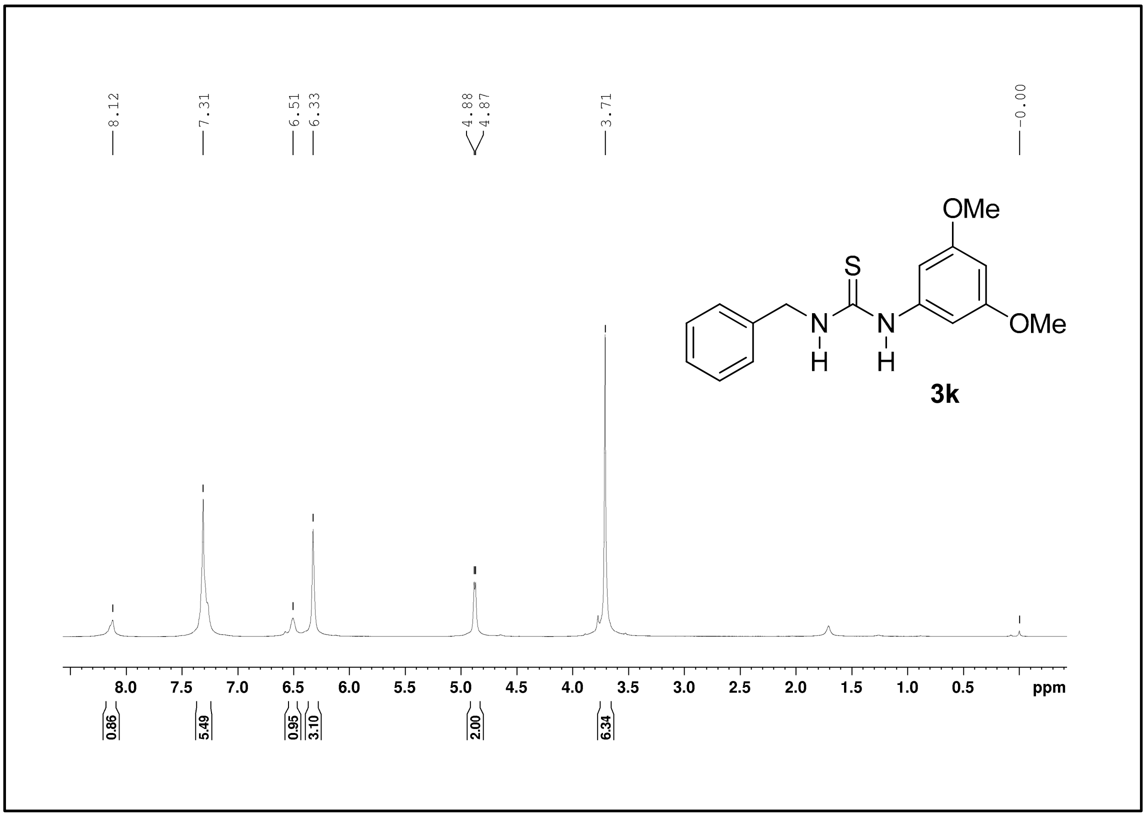

(A)

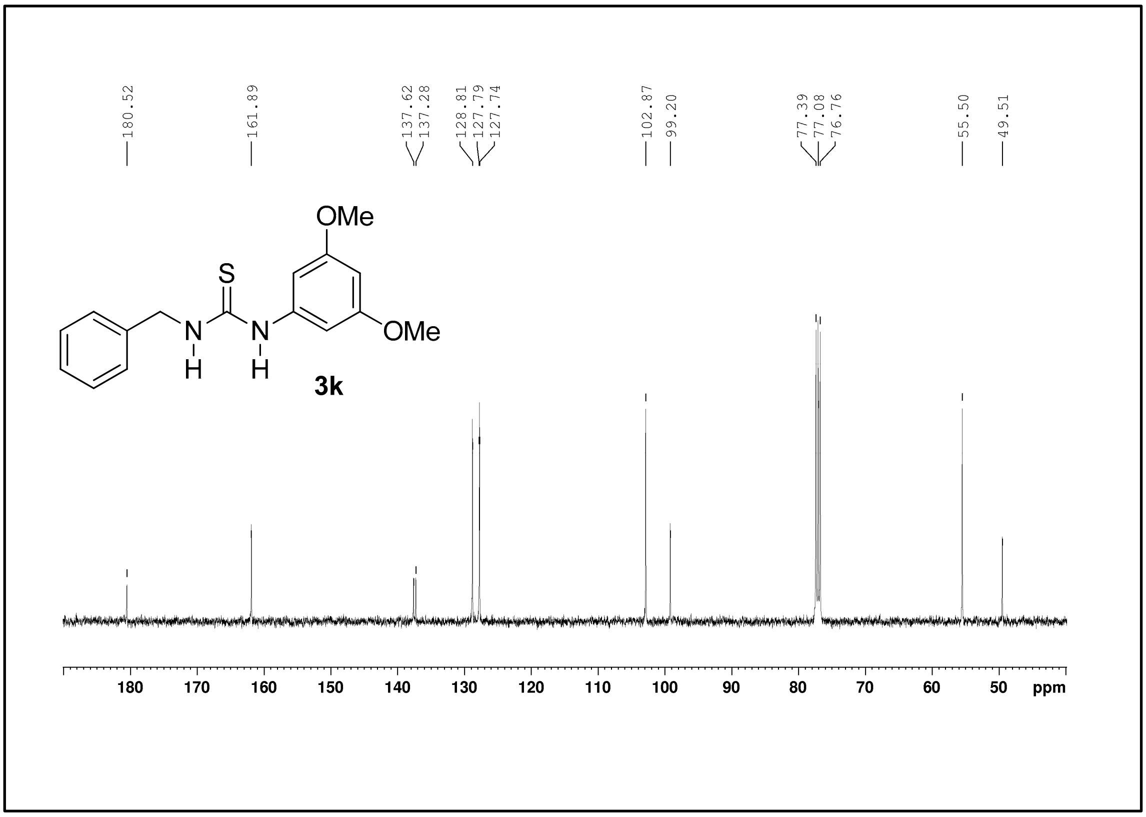

(B)

Figure S6. ^{1}H-NMR spectrum (A) and ^{13}C-NMR spectrum (B) of thiourea **3k**.

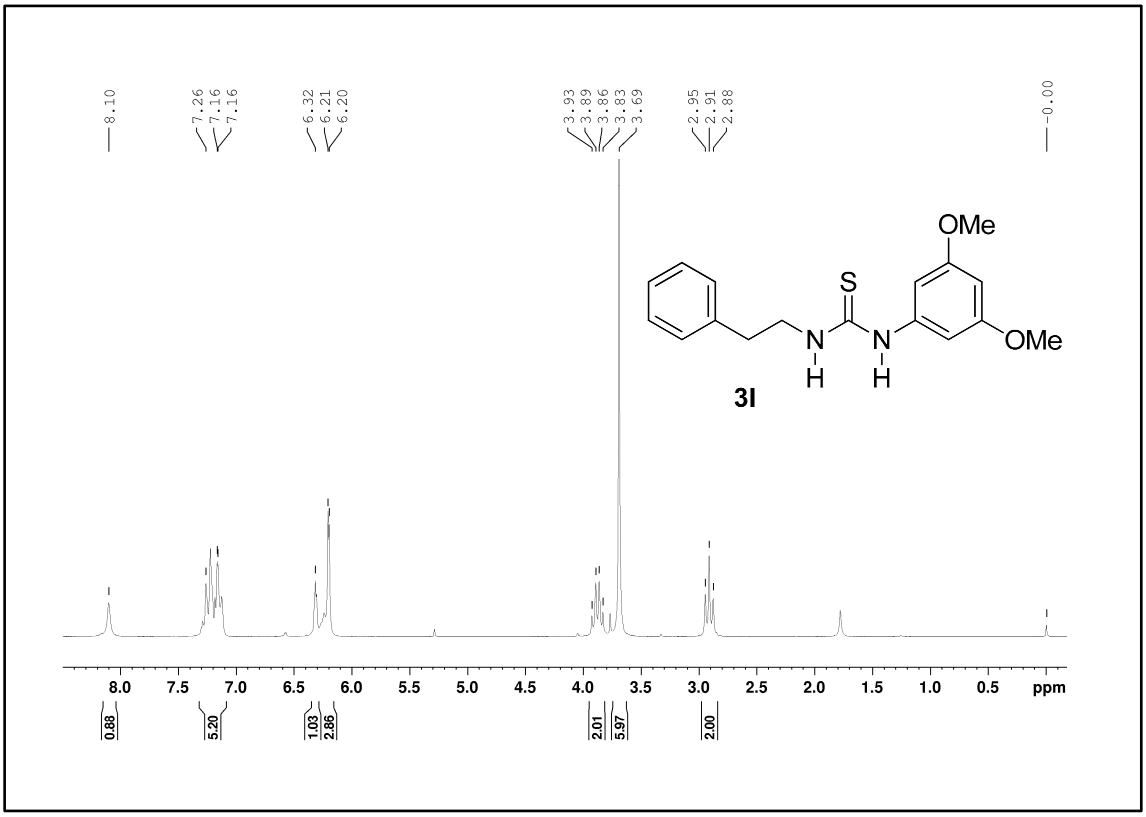

(A)

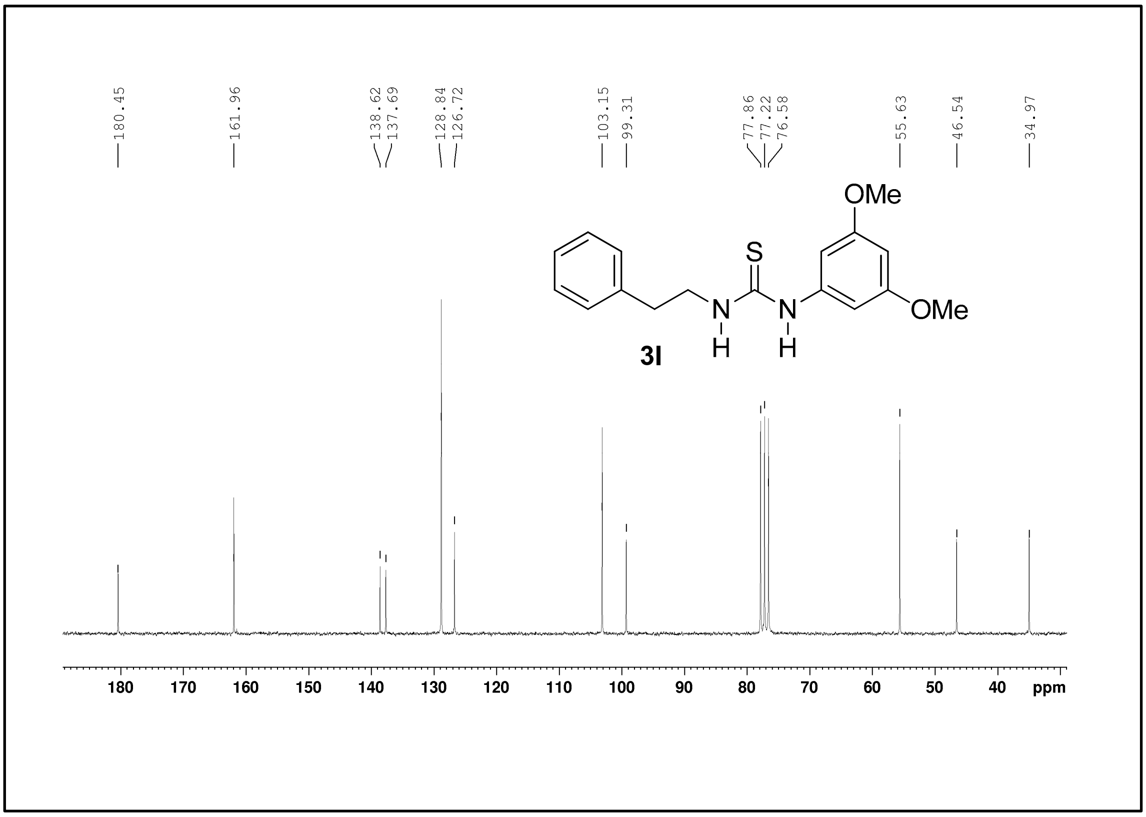

(B)

Figure S7. ^{1}H-NMR spectrum (A) and ^{13}C-NMR spectrum (B) of thiourea **3l**.

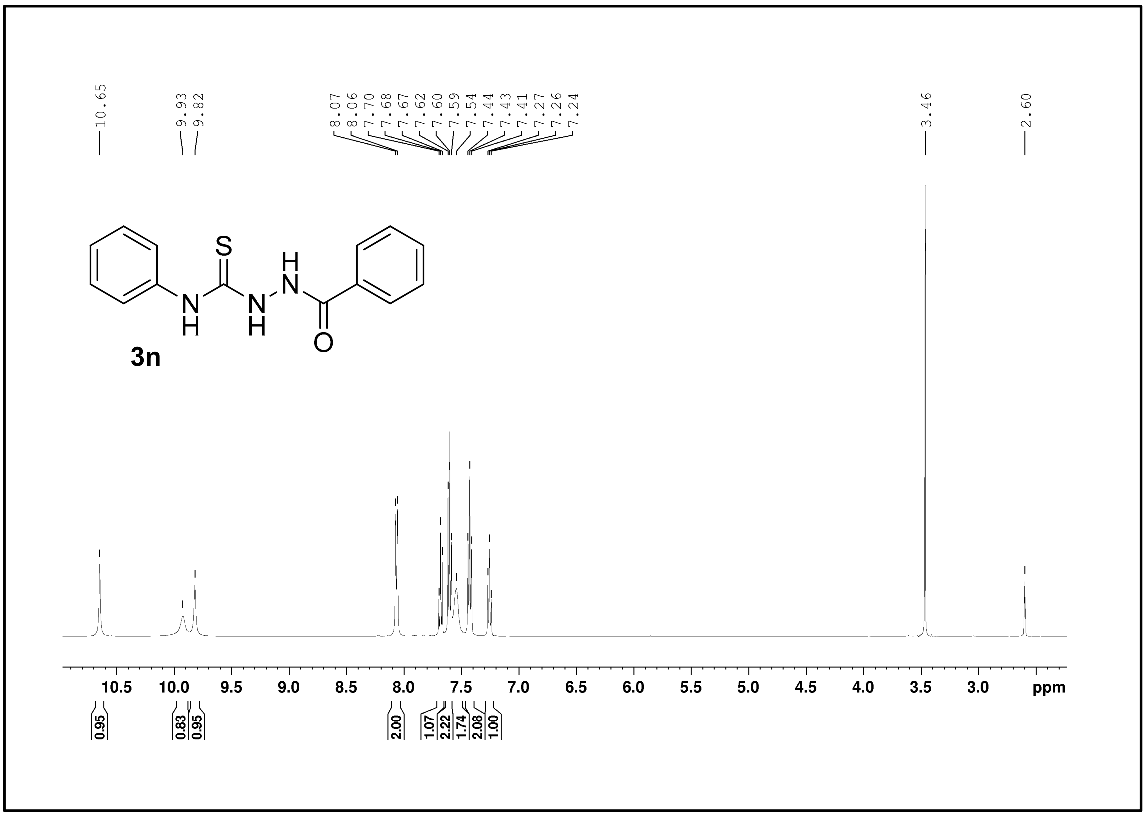

(A)

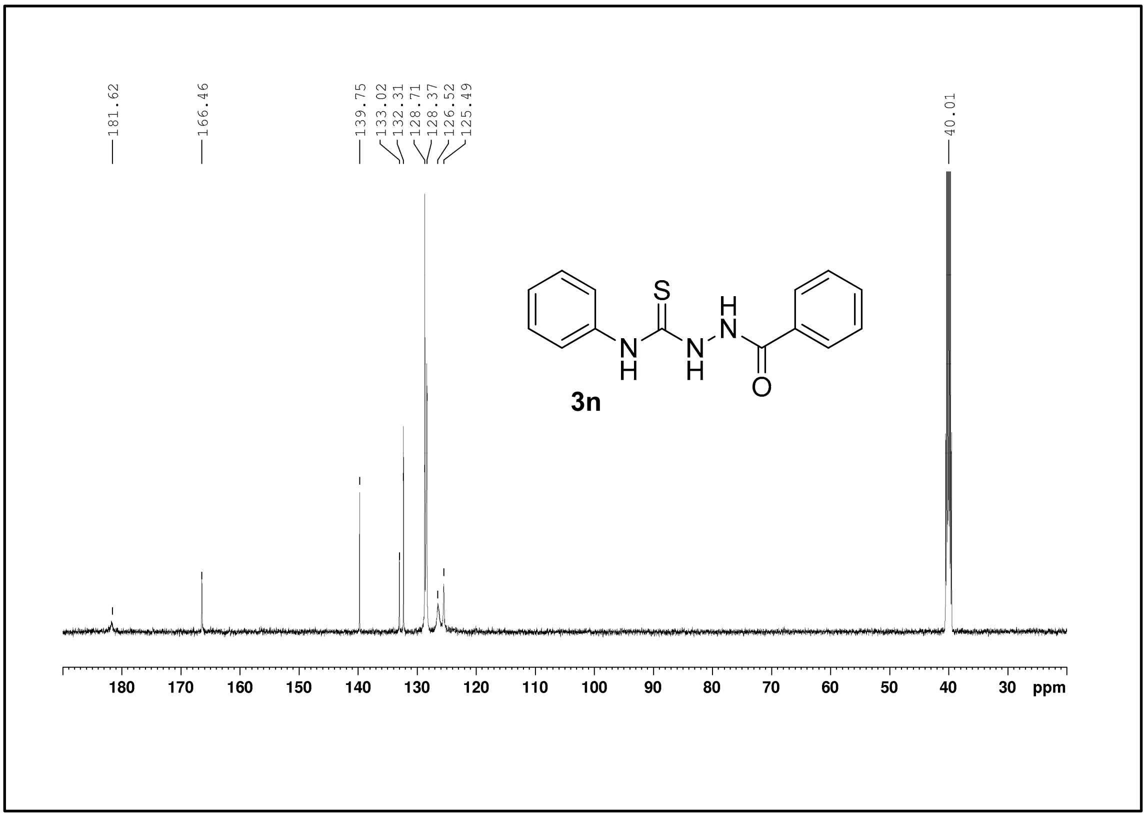

(B)

Figure S8. ^{1}H-NMR spectrum (A) and ^{13}C-NMR spectrum (B) of thiourea **3n**.

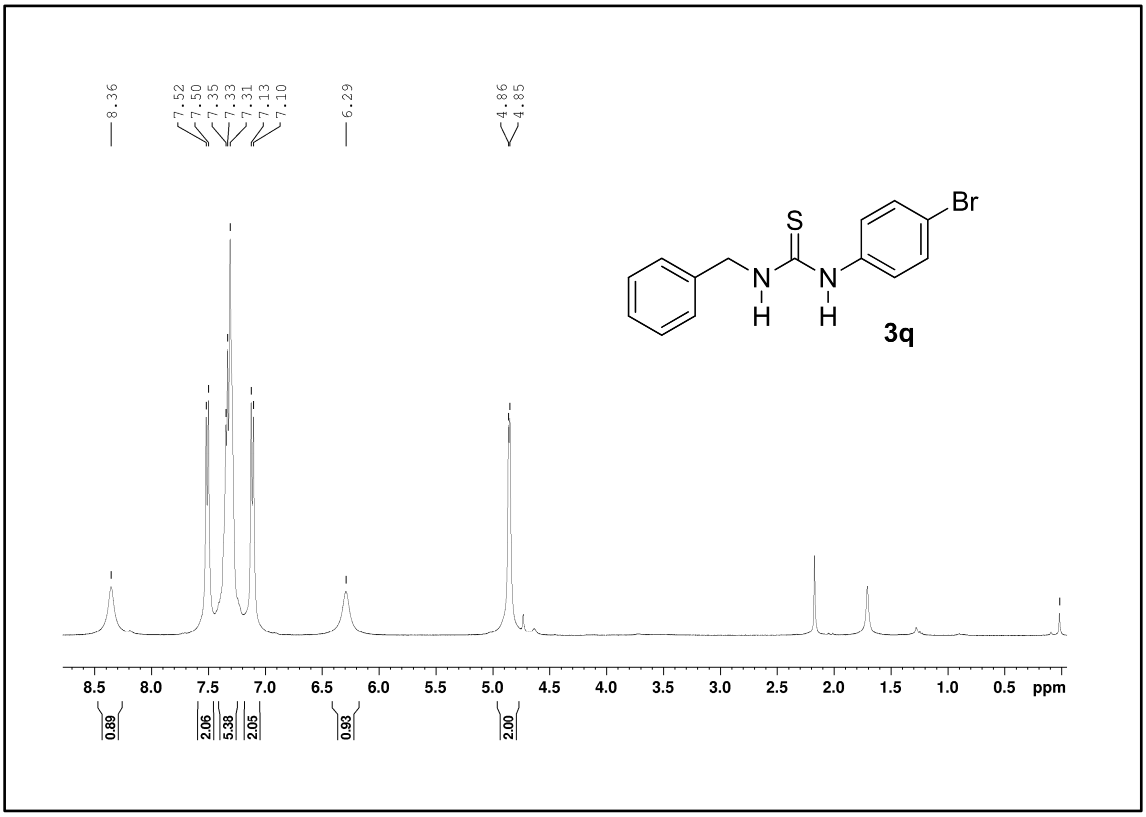

(A)

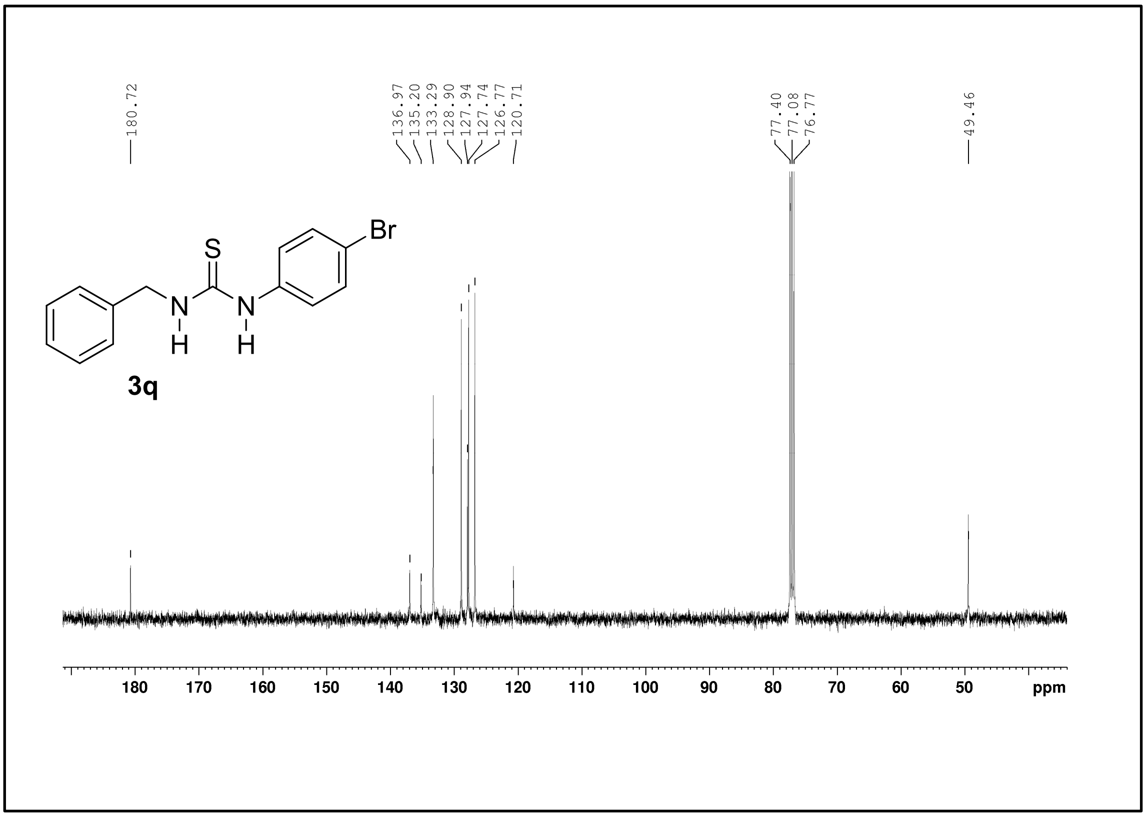

(B)

Figure S9. ^{1}H-NMR spectrum (A) and ^{13}C-NMR spectrum (B) of thiourea **3q**.

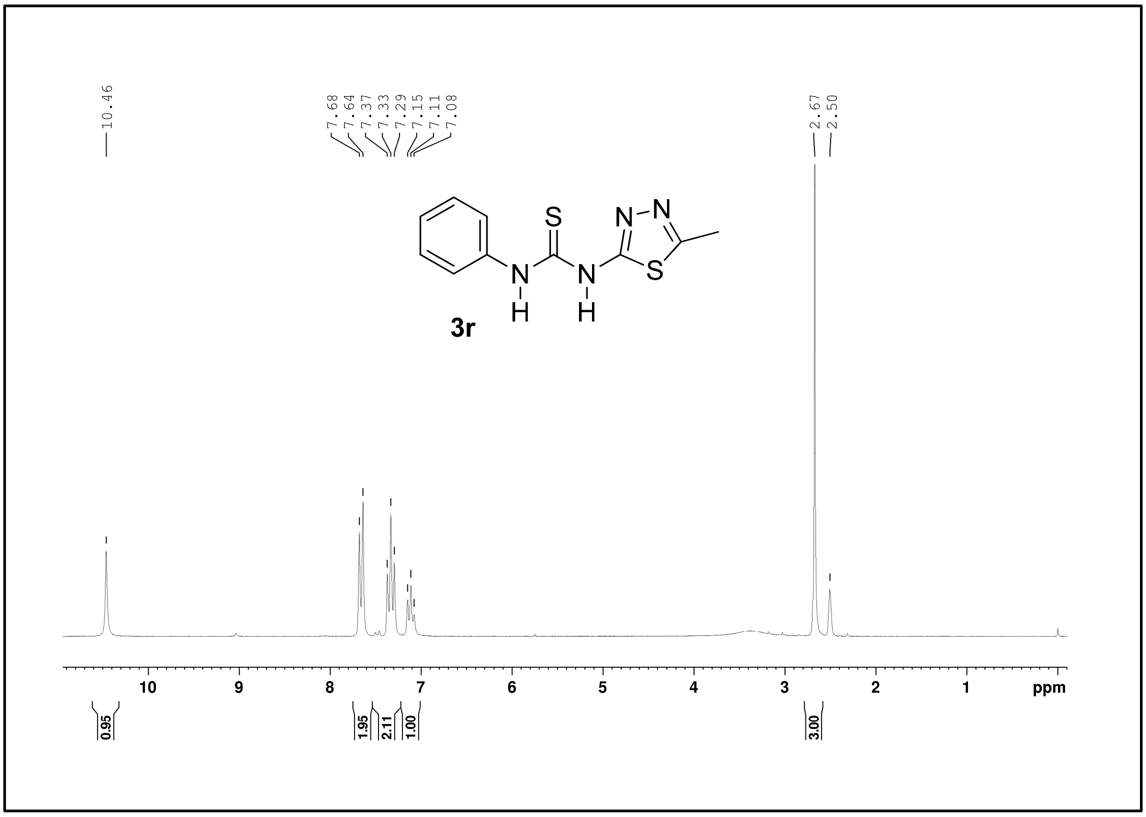

(A)

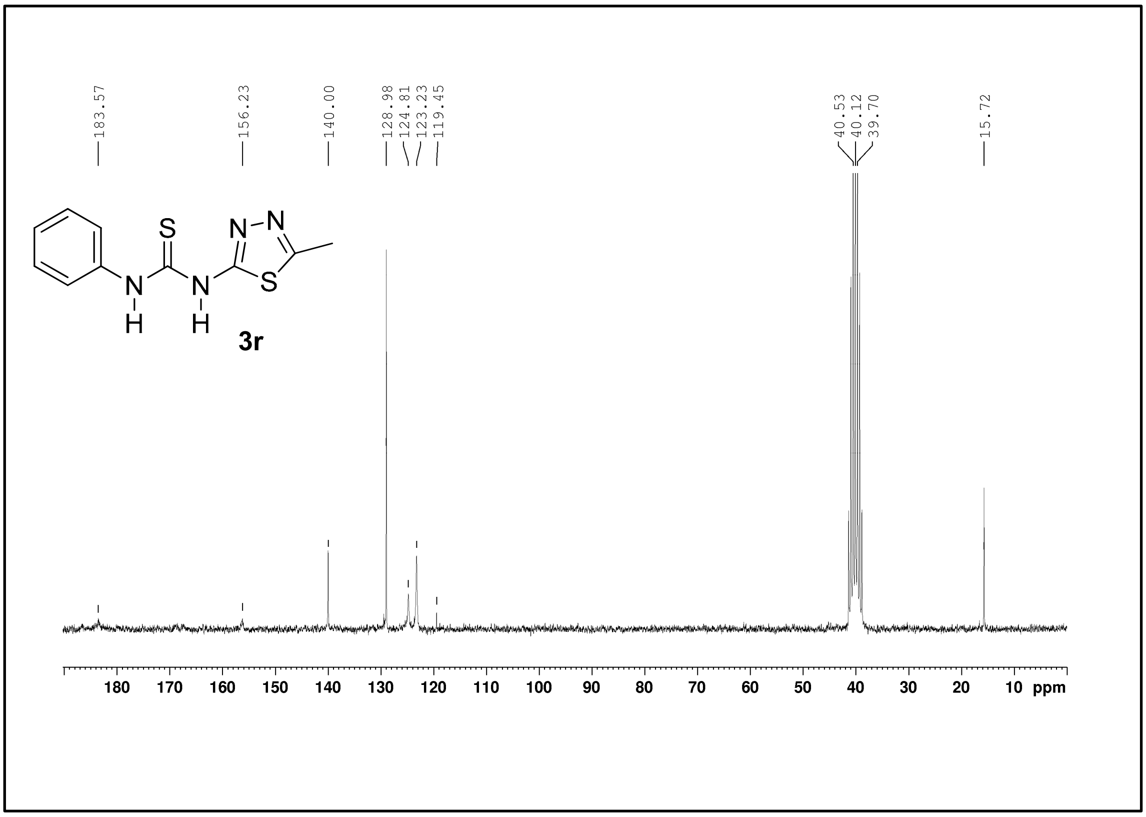

(B)

Figure S10. ^{1}H-NMR spectrum (A) and ^{13}C-NMR spectrum (B) of thiourea **3r**.

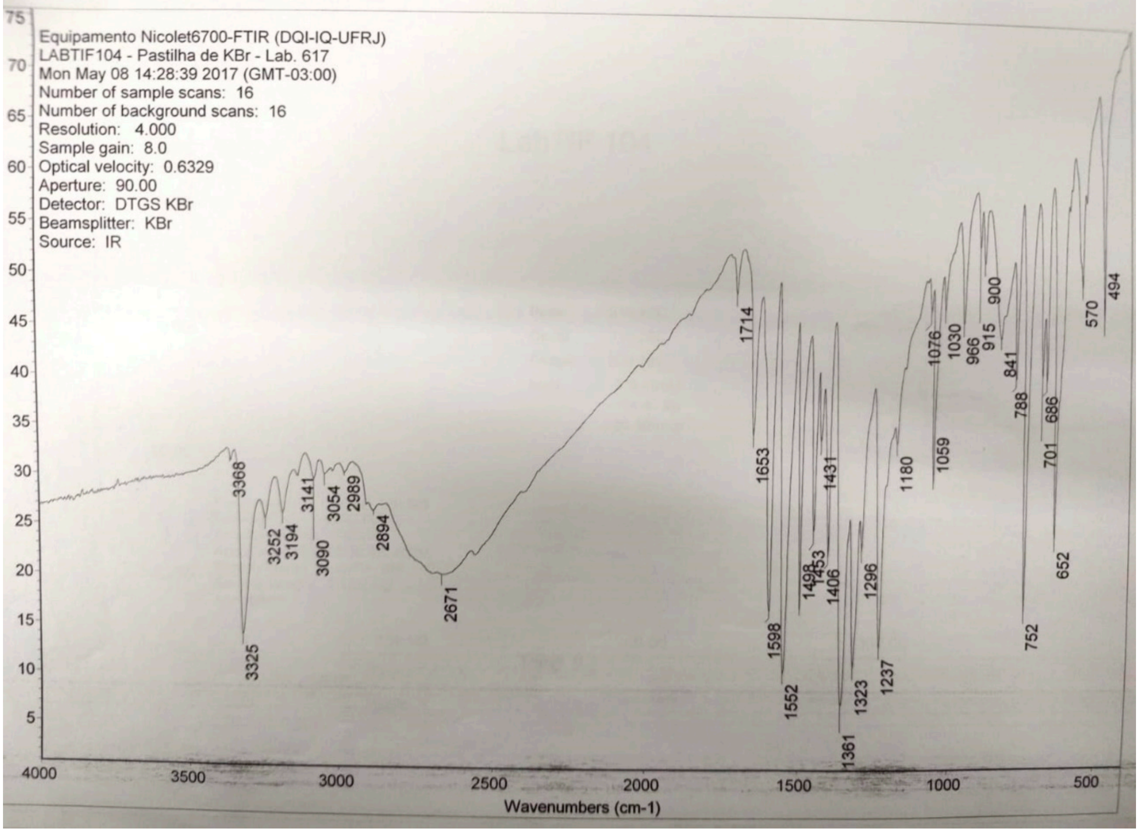

Figure S11. FT-IR spectrum of thiourea **3r**.

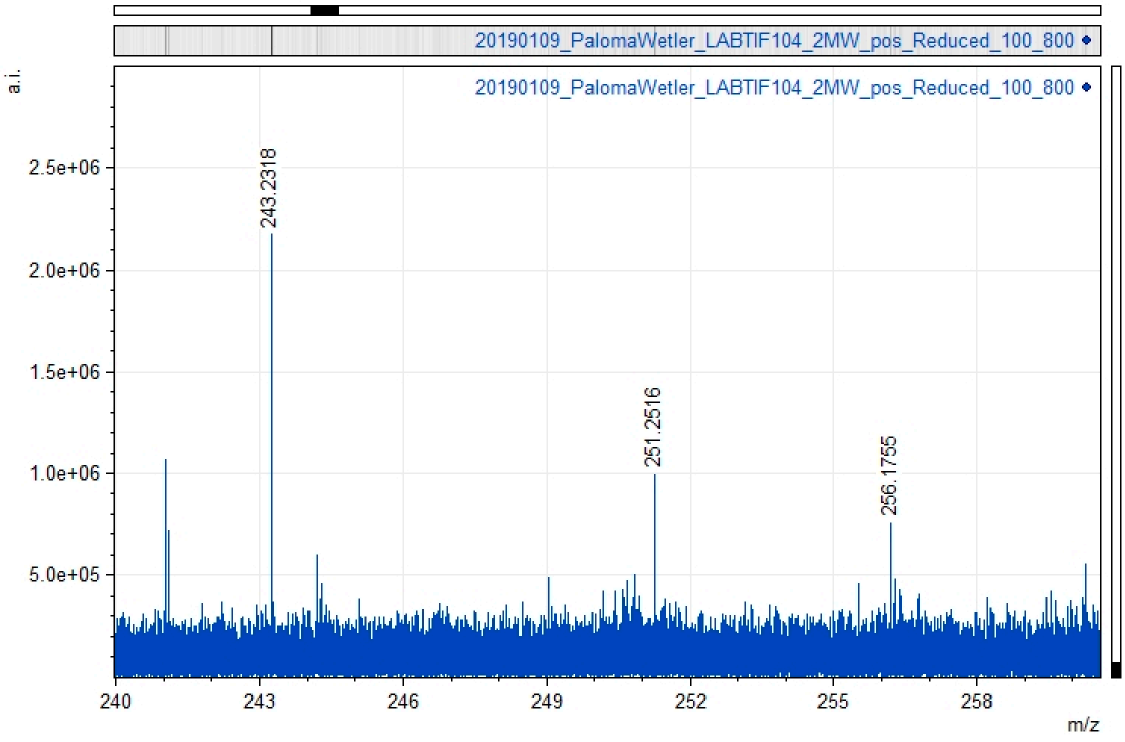

Figure S12. HR-MS spectrum of thiourea **3r**.

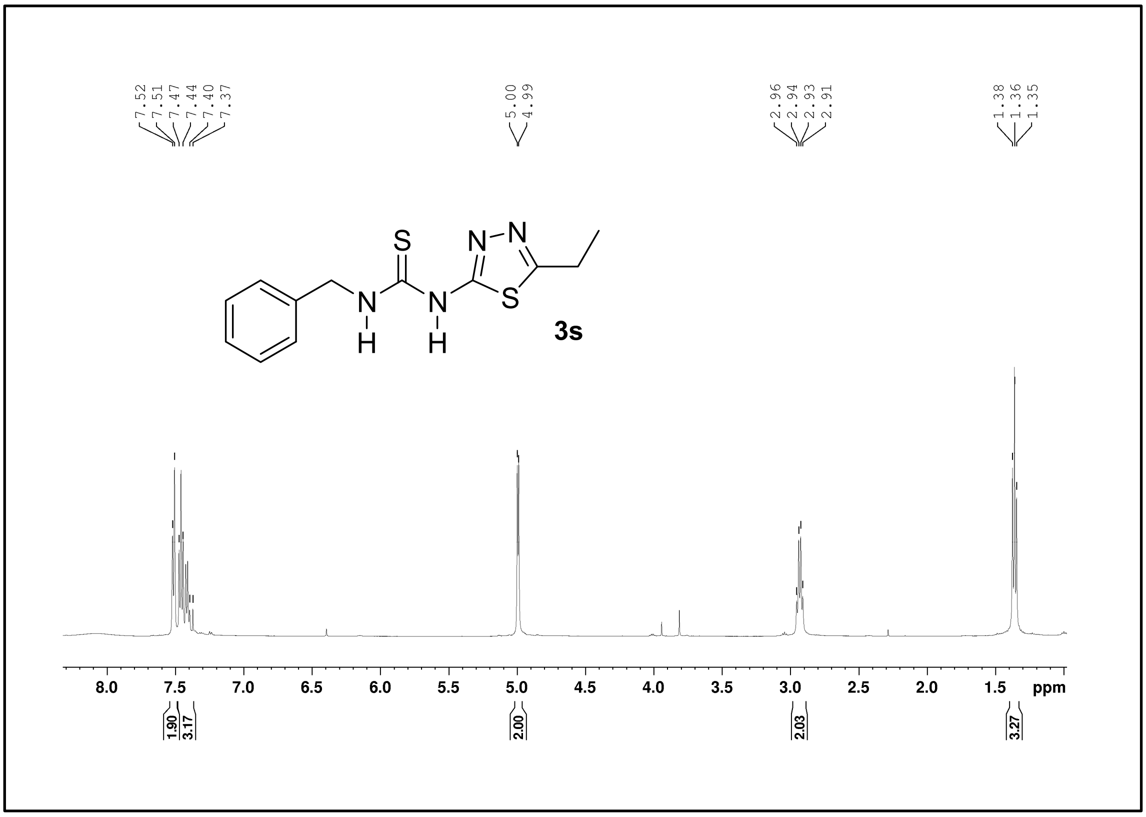

(A)

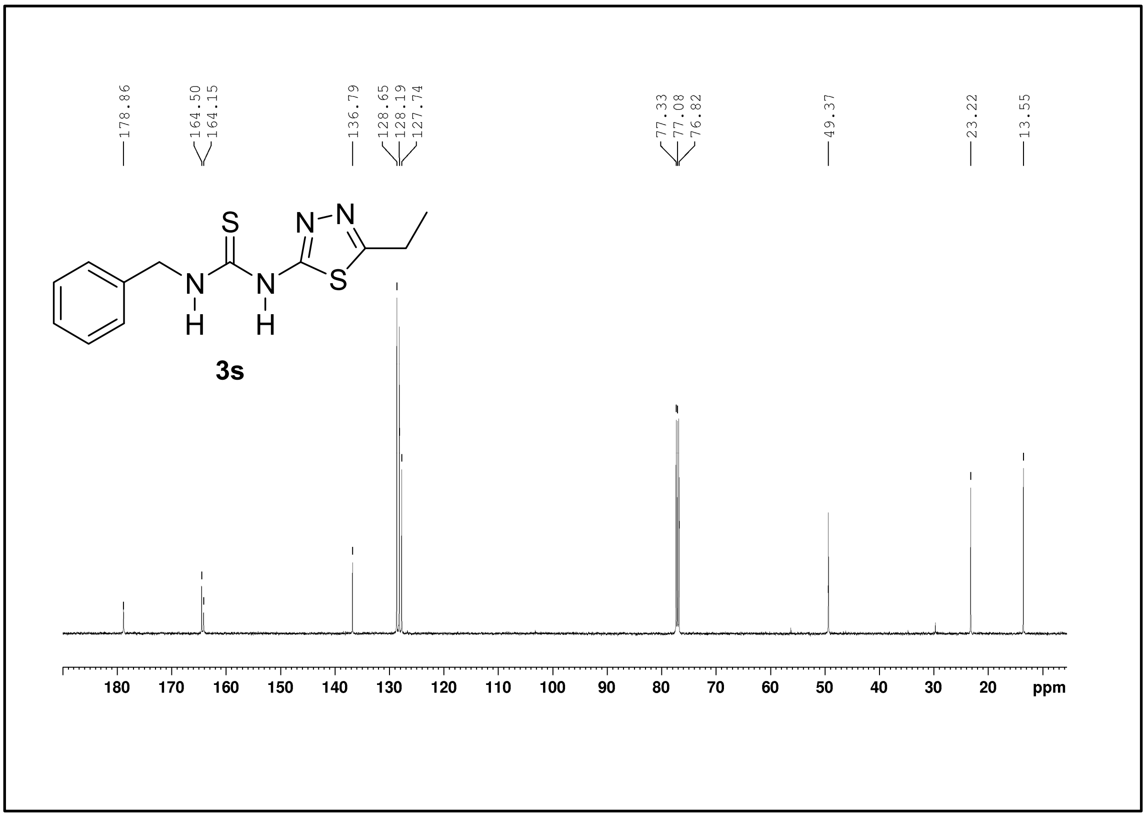

(B)

Figure S13. ^{1}H-NMR spectrum (A) and ^{13}C-NMR spectrum (B) of thiourea **3s**.

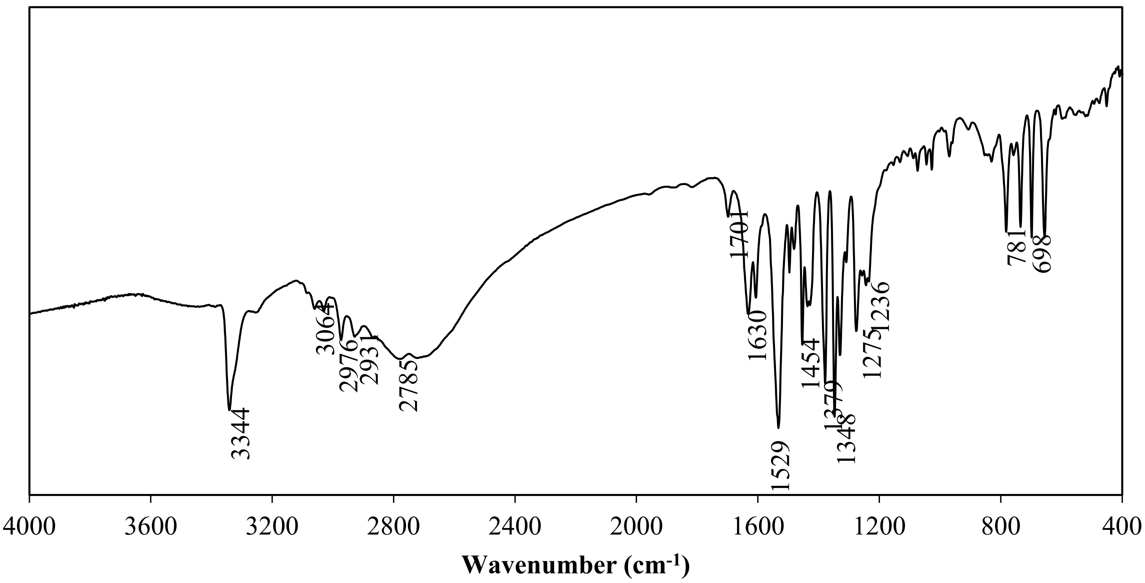

Figure S14. FT-IR spectrum of thiourea **3s**.

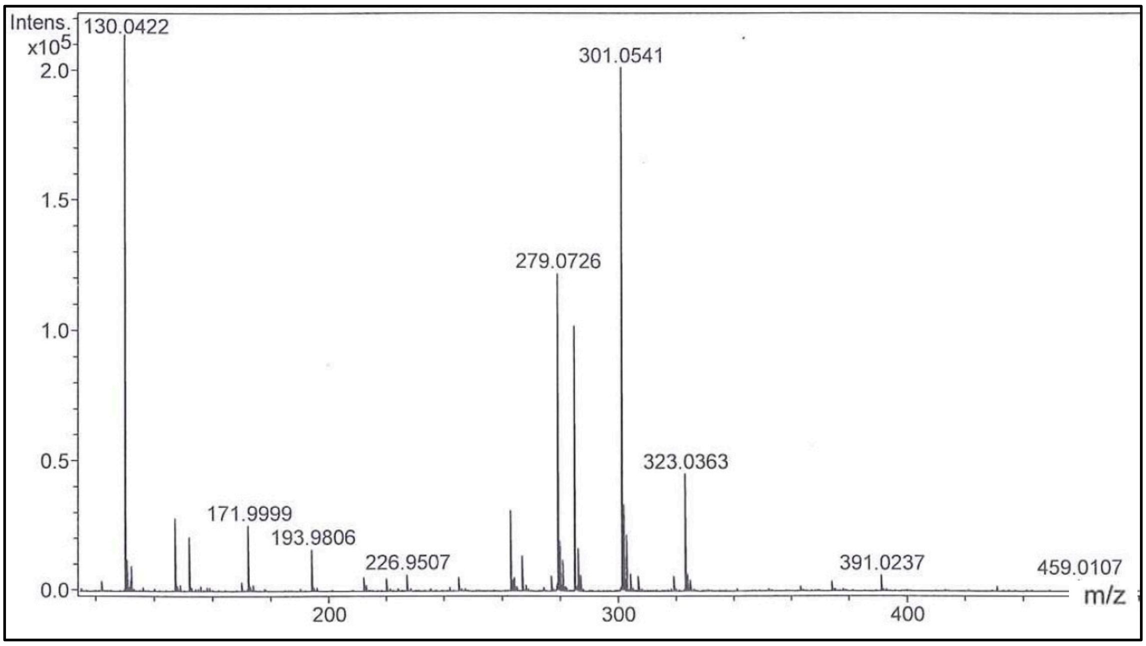

Figure S15. HR-MS spectrum of thiourea **3s**.

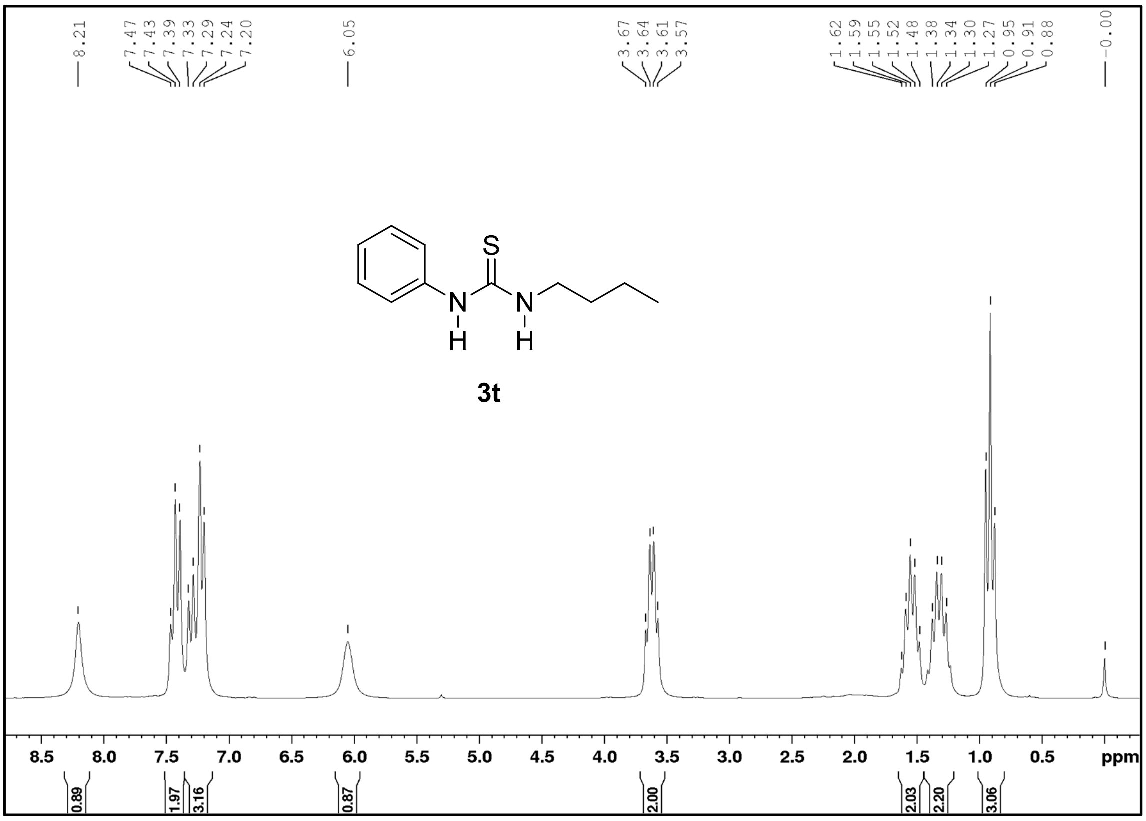
8.21
7.47
7.43
7.39
7.33
7.29
7.24
7.20
6.05
3.67
3.64
3.61
3.57
1.62
1.59
1.55
1.52
1.48
1.38
1.34
1.30
1.27
0.95
0.91
0.88
-0.00
S
N
H
N
H
3t
8.5 8.0 7.5 7.0 6.5 6.0 5.5 5.0 4.5 4.0 3.5 3.0 2.5 2.0 1.5 1.0 0.5 ppm
0.89
1.97
3.16
0.87
2.00
2.03
2.20
3.06

(A)

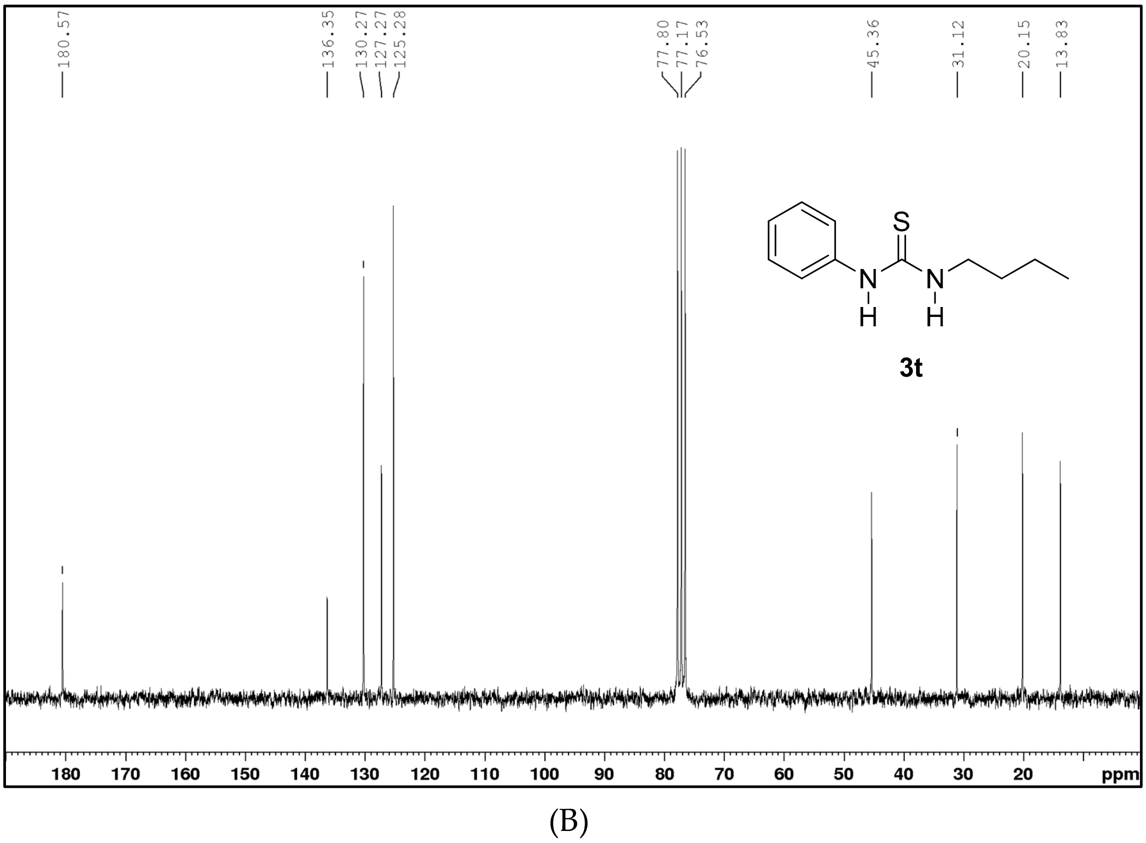
180.57
136.35
130.27
127.27
125.28
77.80
77.17
76.53
45.36
31.12
20.15
13.83
S
N
H
N
H
3t
180 170 160 150 140 130 120 110 100 90 80 70 60 50 40 30 20 ppm

(B)

Figure S16. ^{1}H-NMR spectrum (A) and ^{13}C-NMR spectrum (B) of thiourea **3t**.

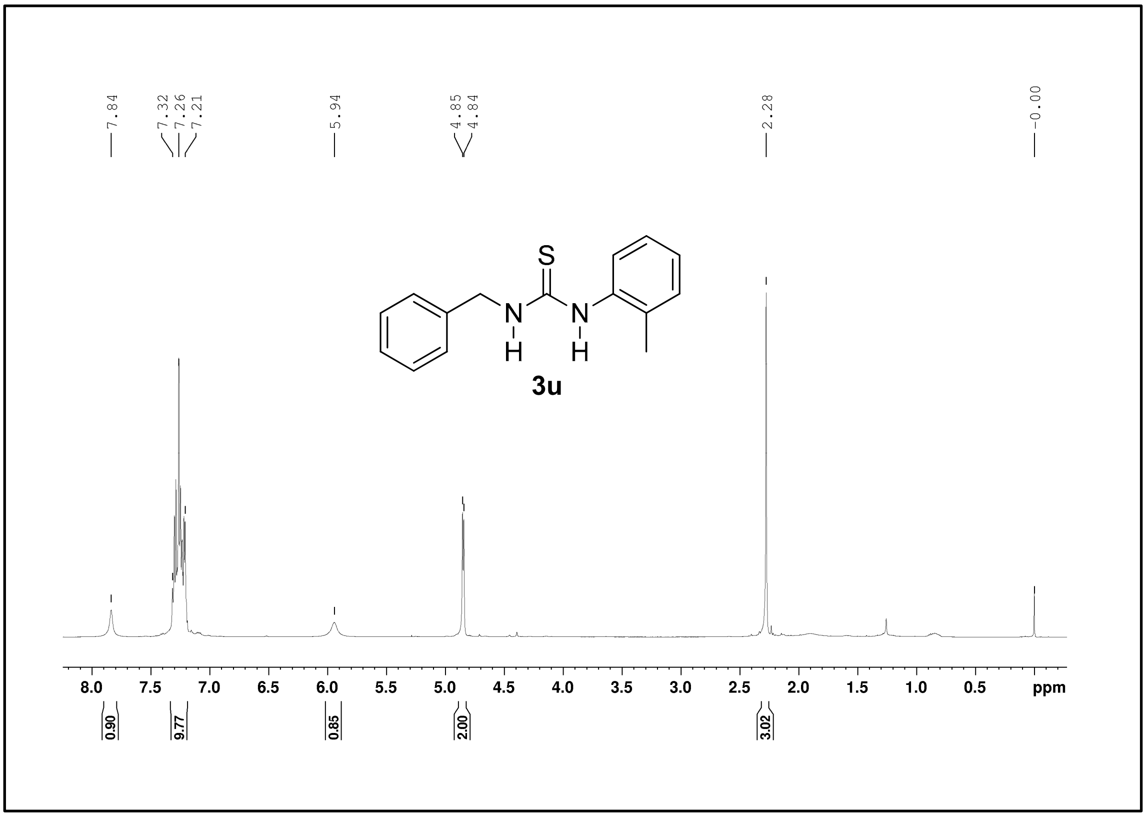

(A)

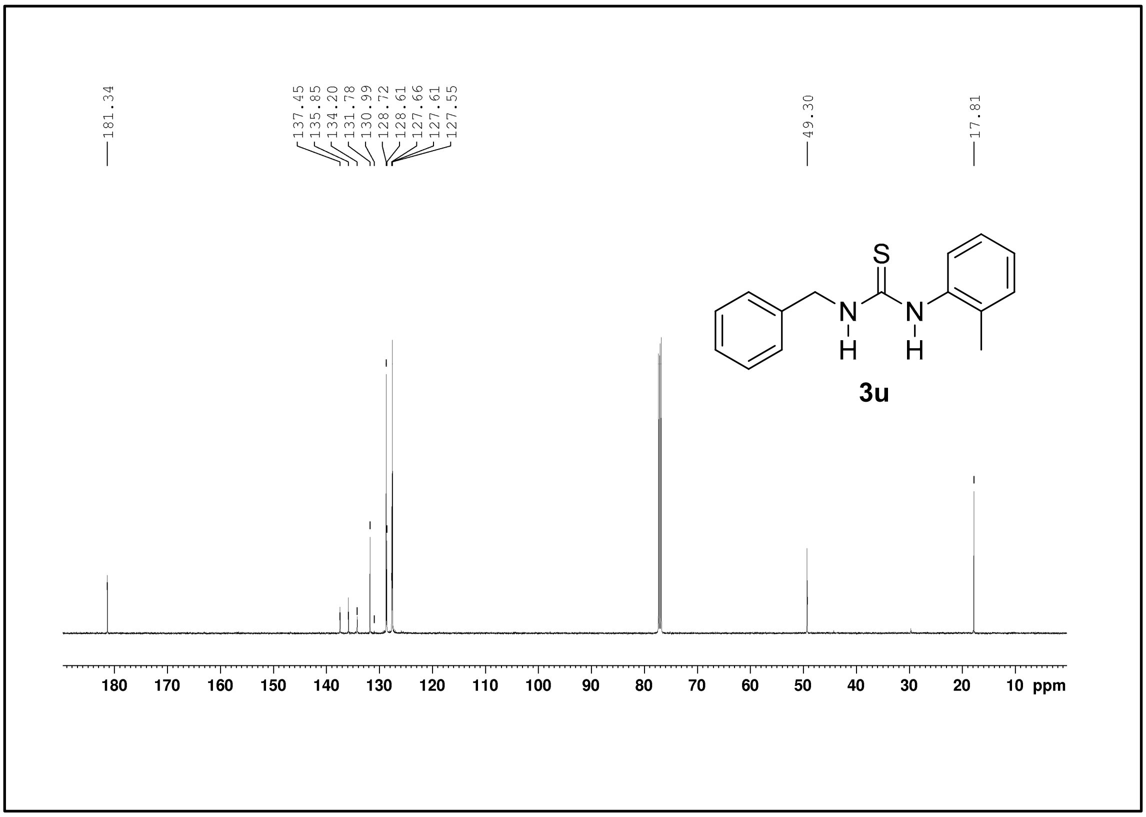

(B)

Figure S17. ^{1}H-NMR spectrum (A) and ^{13}C-NMR spectrum (B) of thiourea **3u**.

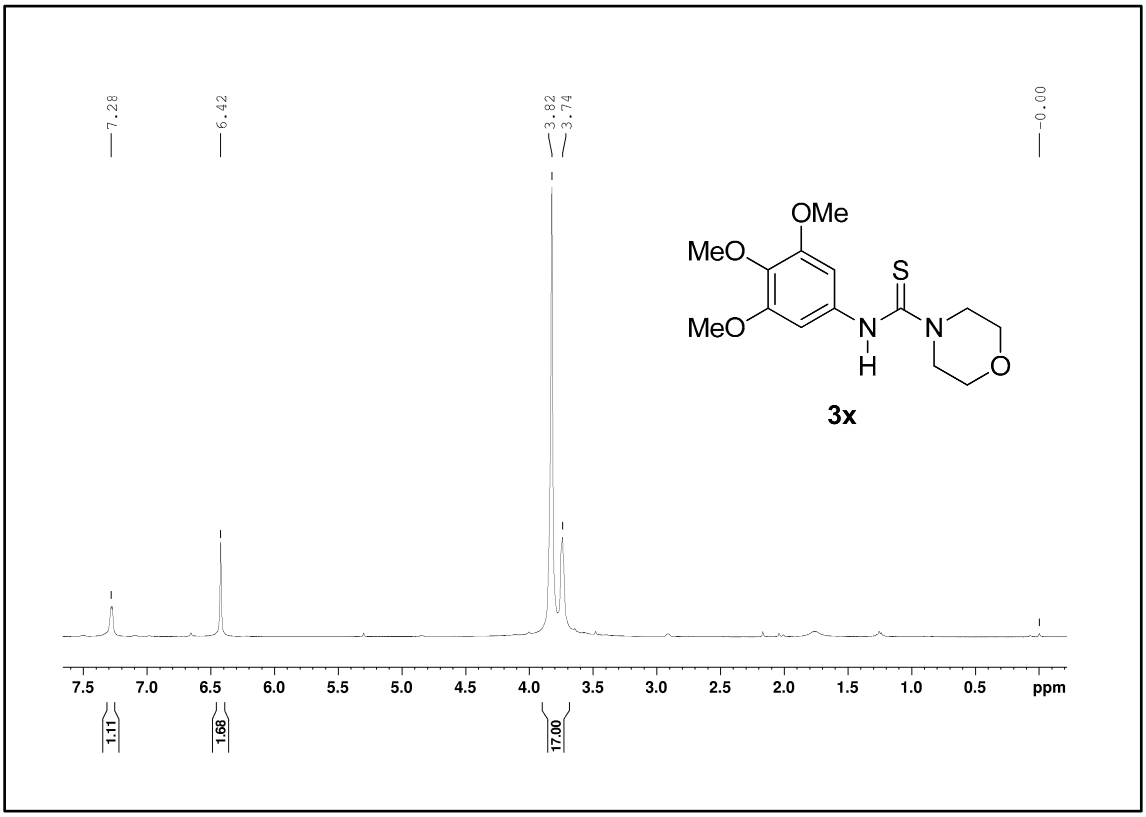

(A)

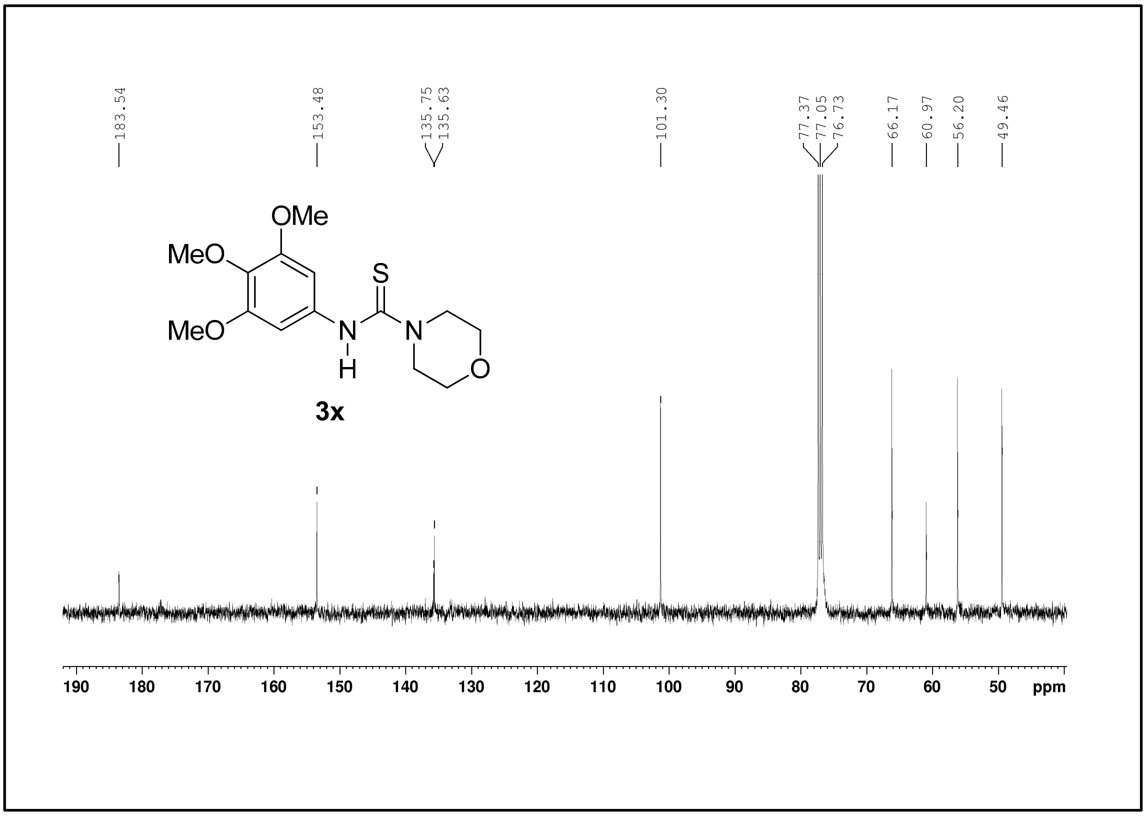

(B)

Figure S18. ^{1}H-NMR spectrum (A) and ^{13}C-NMR spectrum (B) of thiourea **3x**.

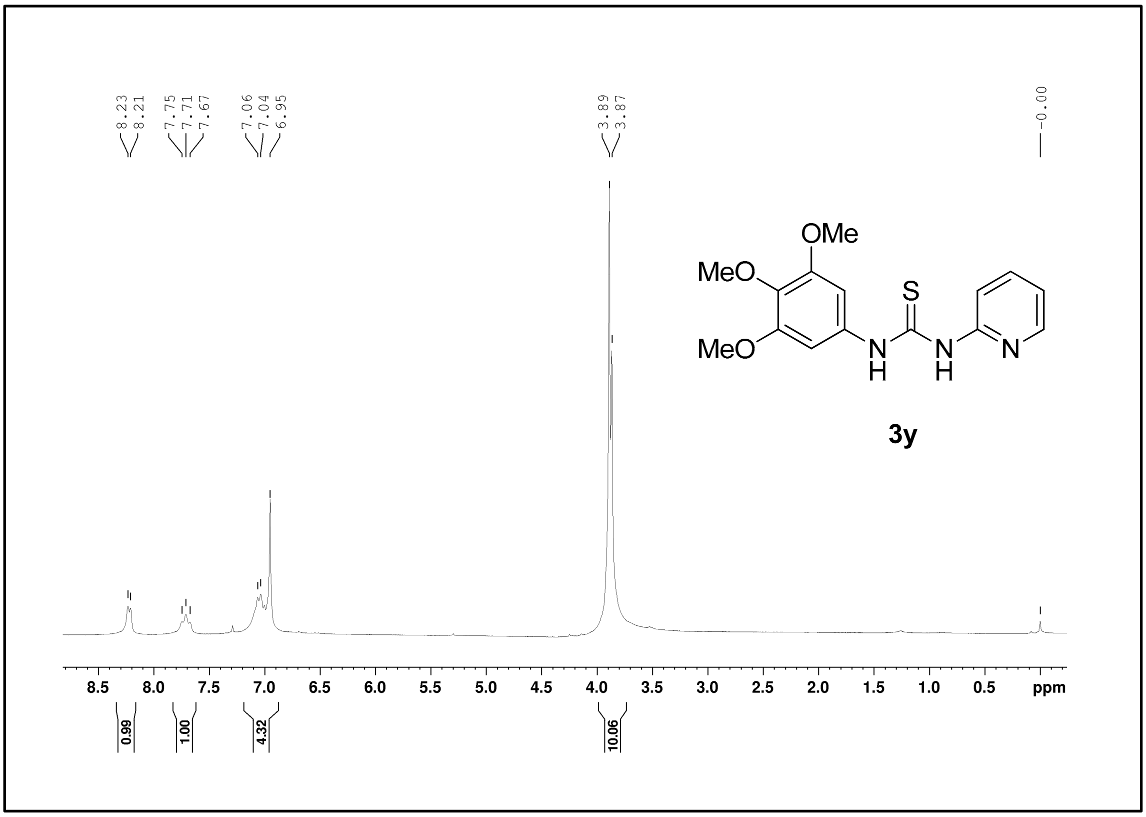

(A)

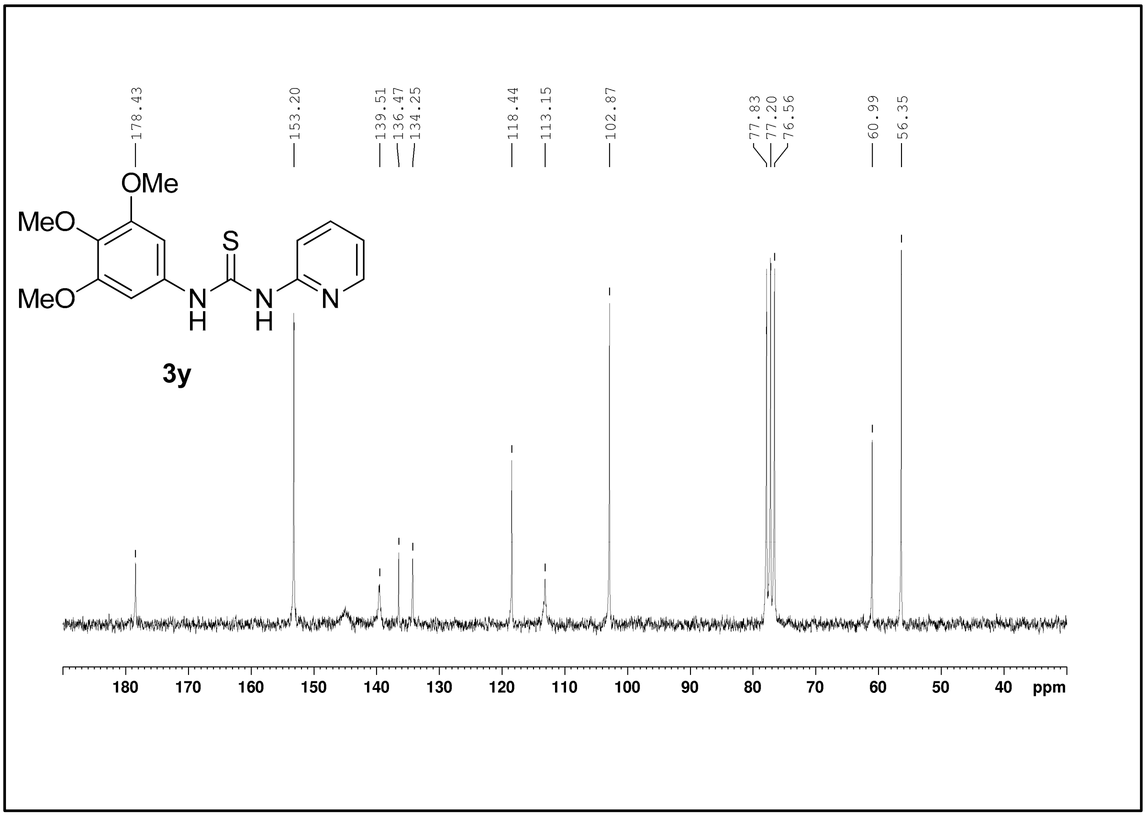

(B)

Figure S19. ^{1}H-NMR spectrum (A) and ^{13}C-NMR spectrum (B) of thiourea **3y**.

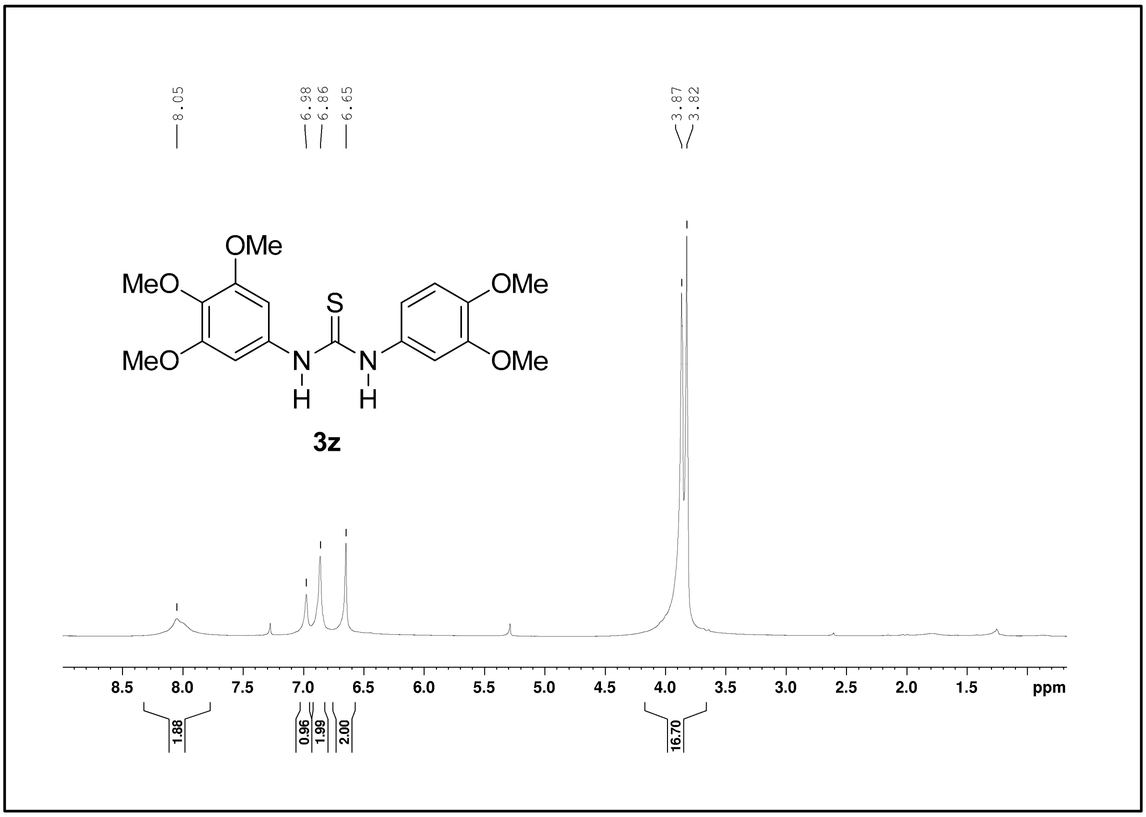

(A)

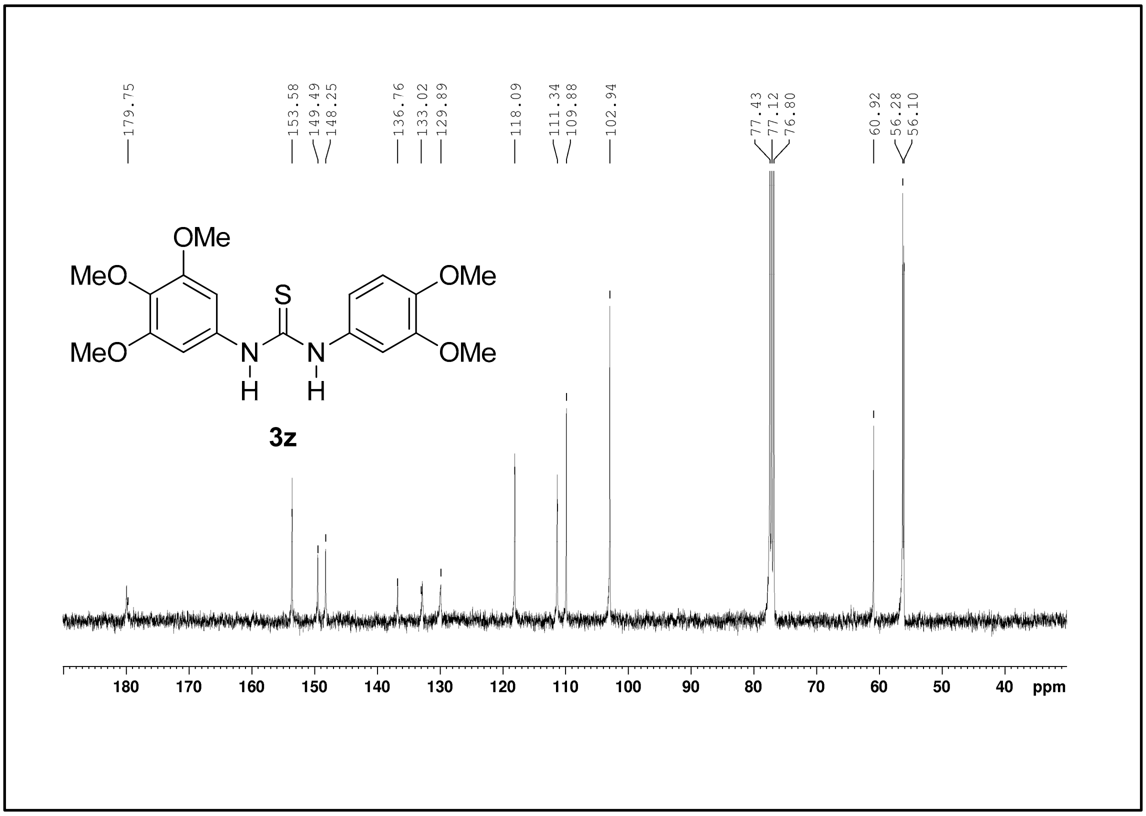

(B)

Figure S20. ¹H-NMR spectrum (A) and ¹³C-NMR spectrum (B) of thiourea **3z**.

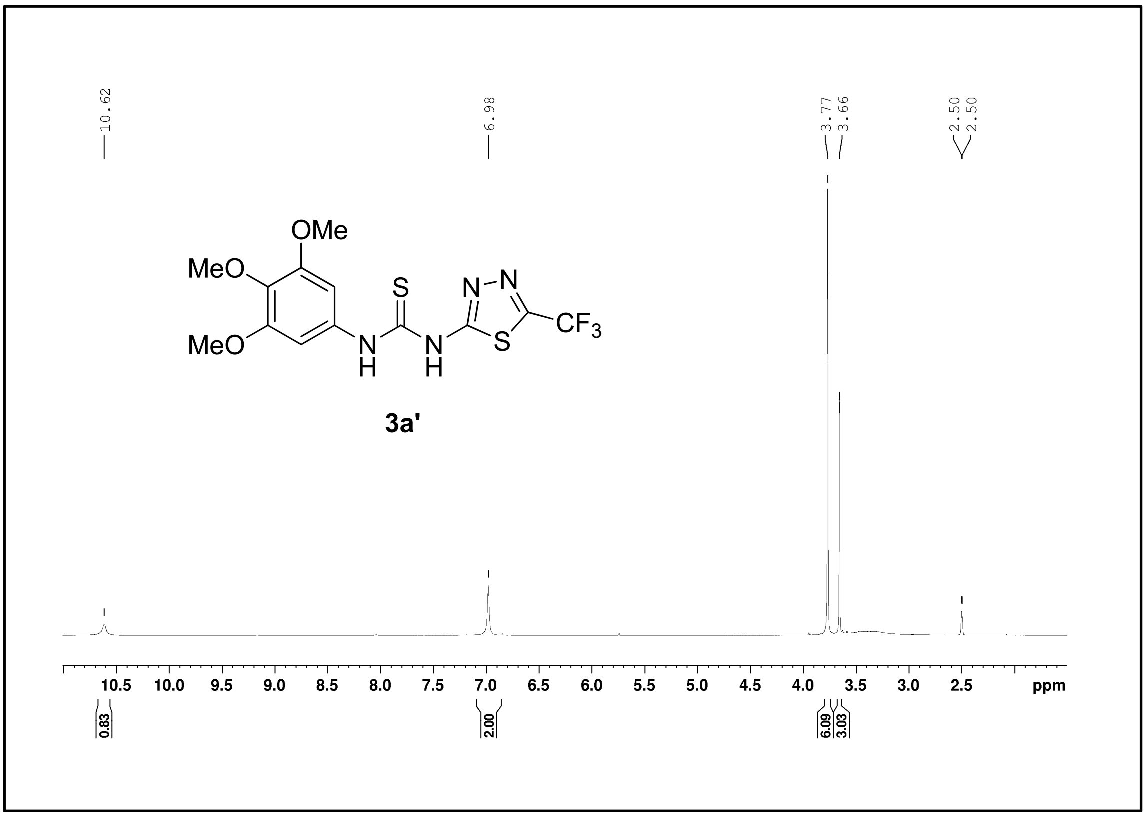

(A)

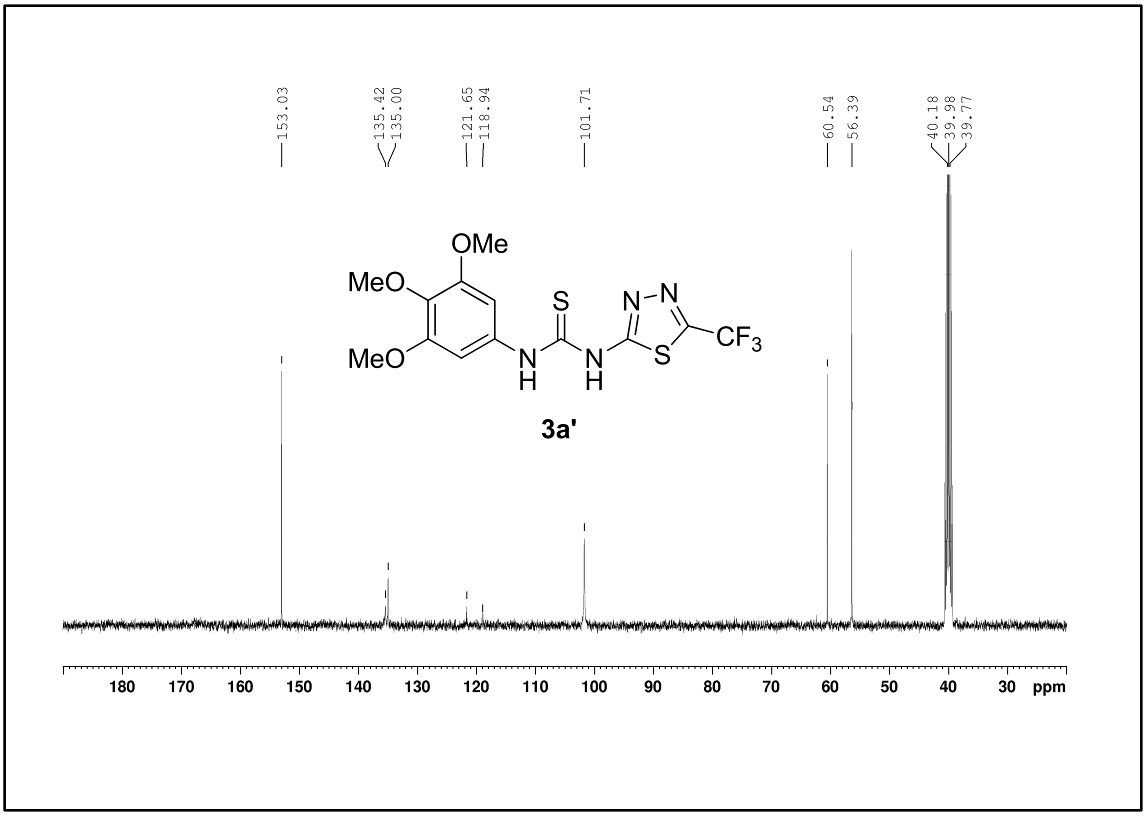

(B)

Figure S21. ^{1}H-NMR spectrum (A) and ^{13}C-NMR spectrum (B) of thiourea **3a′**.

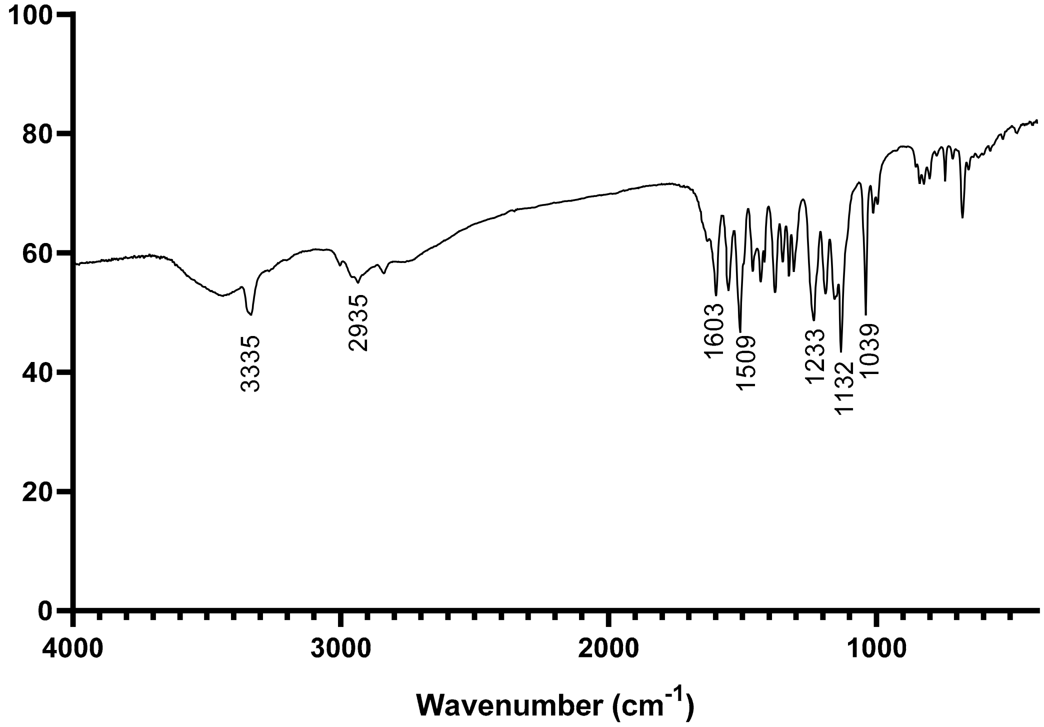

Figure S22. FT-IR spectrum of thiourea **3a′**.

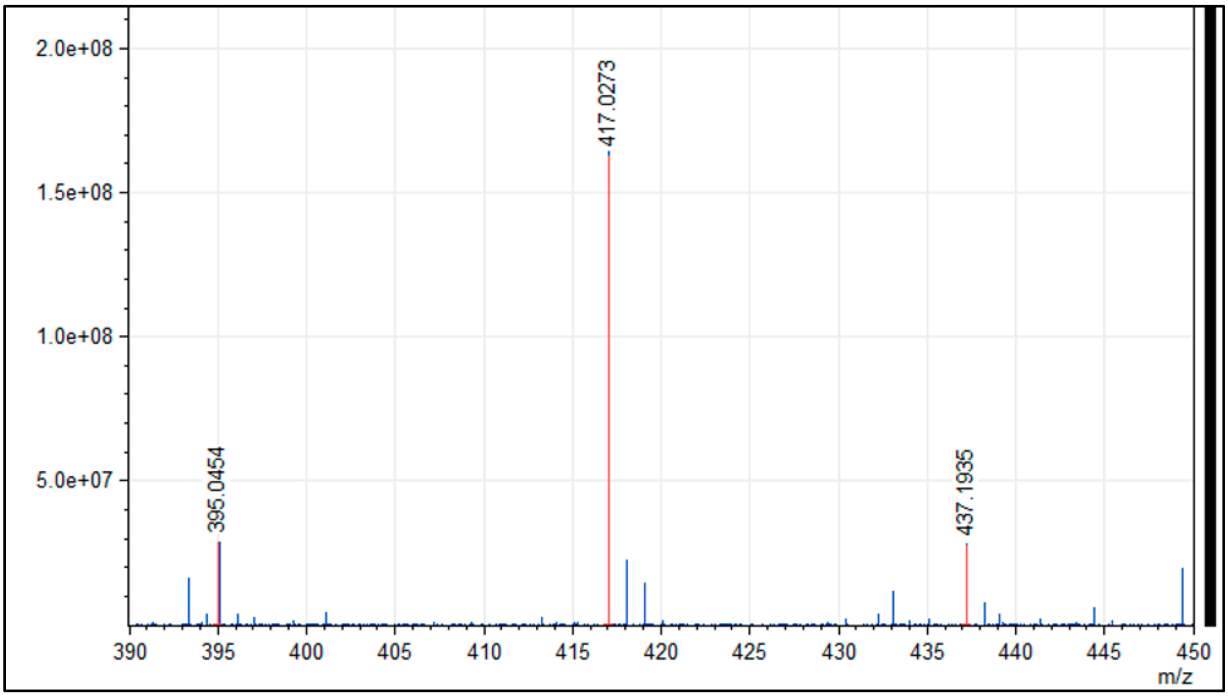

Figure S23. HR-MS spectrum of thiourea **3a′**.

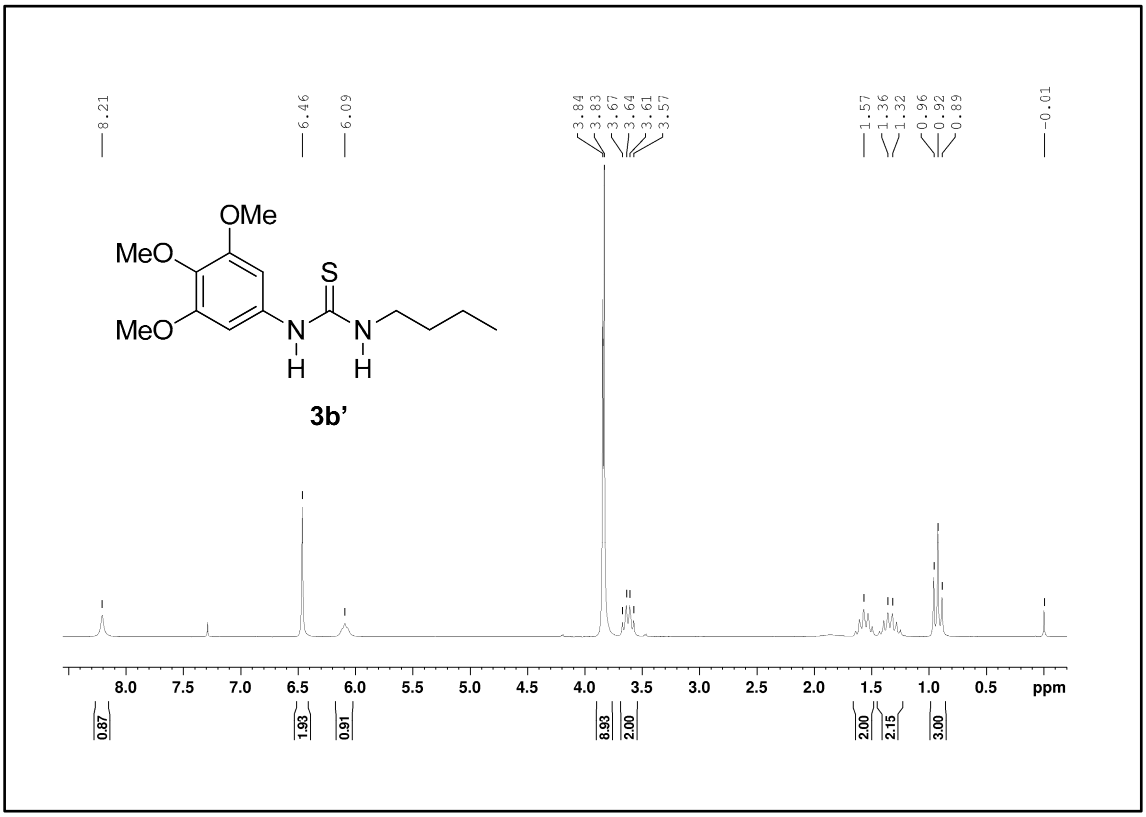

(A)

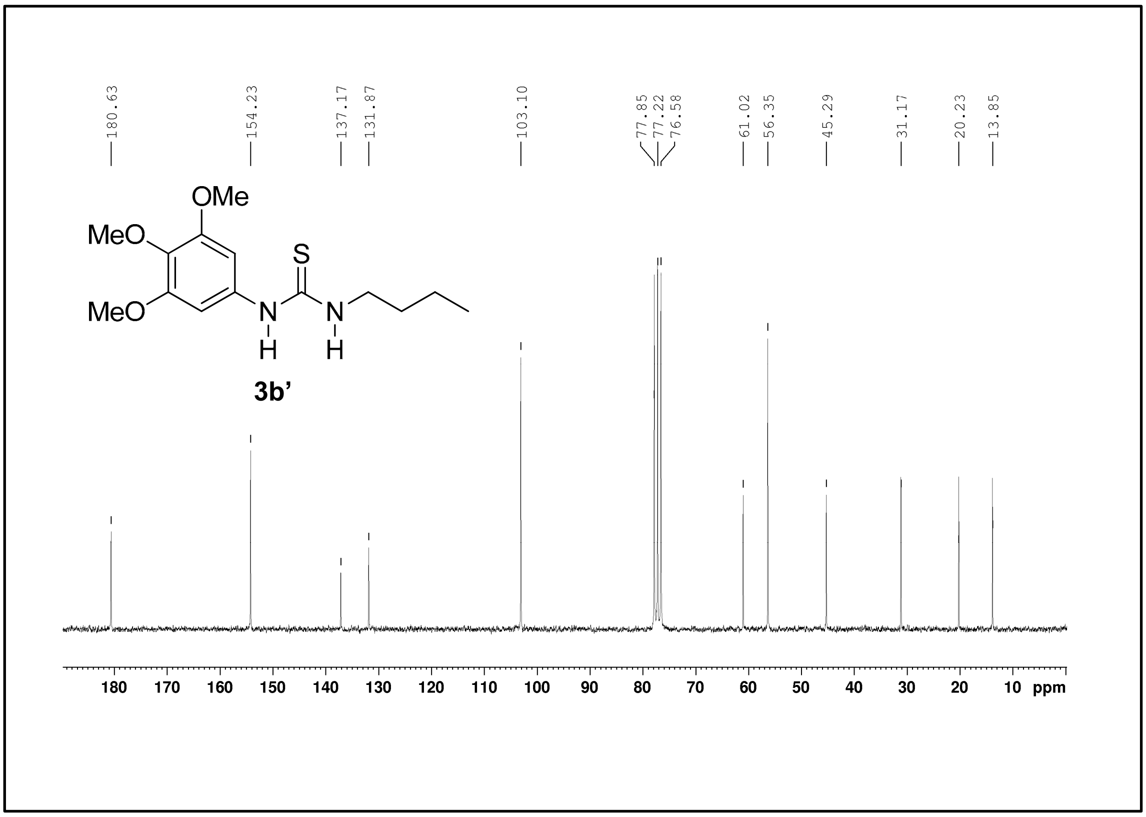

(B)

Figure S24. ^{1}H-NMR spectrum (A) and ^{13}C-NMR spectrum (B) of thiourea **3b′**.

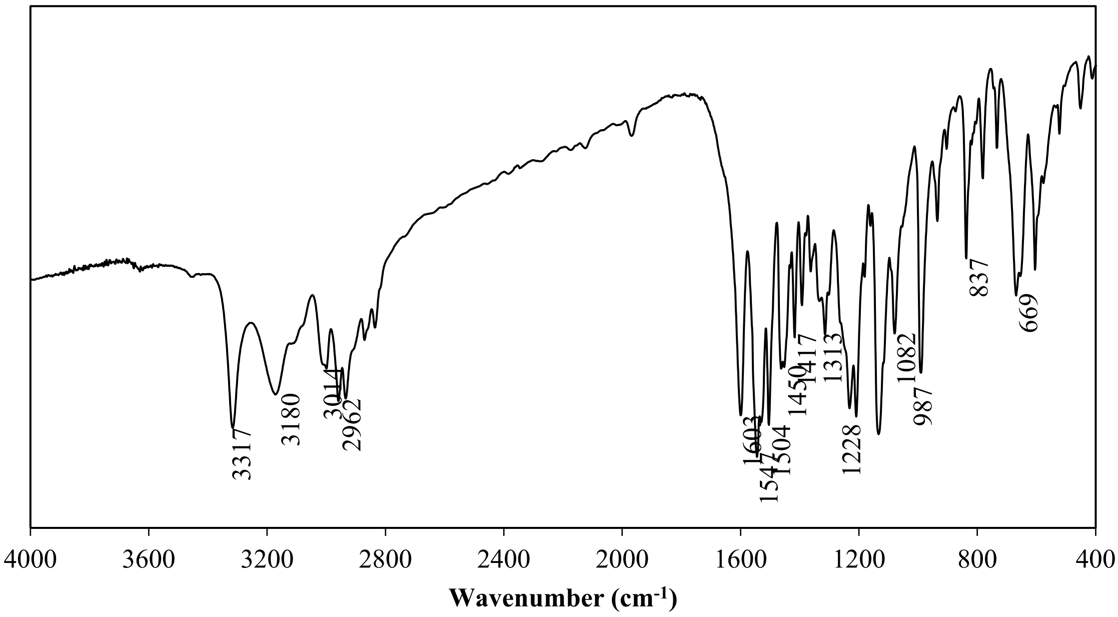

Figure S25. FT-IR spectrum of thiourea **3b′**.

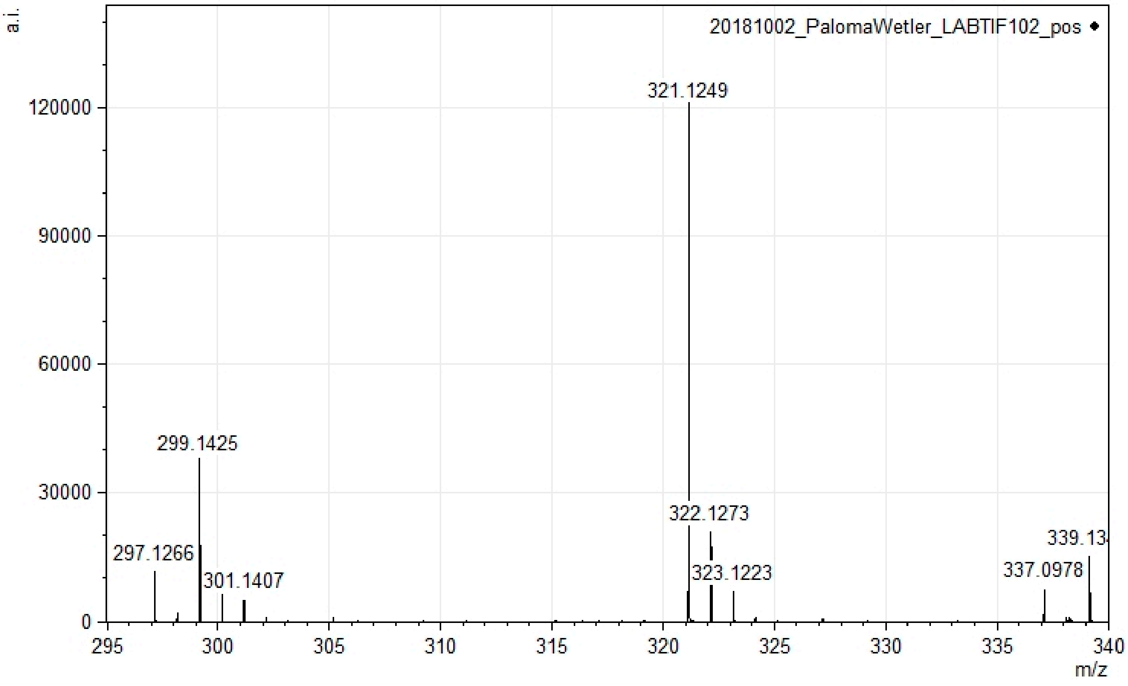

Figure S26. HR-MS spectrum of thiourea **3b′**.

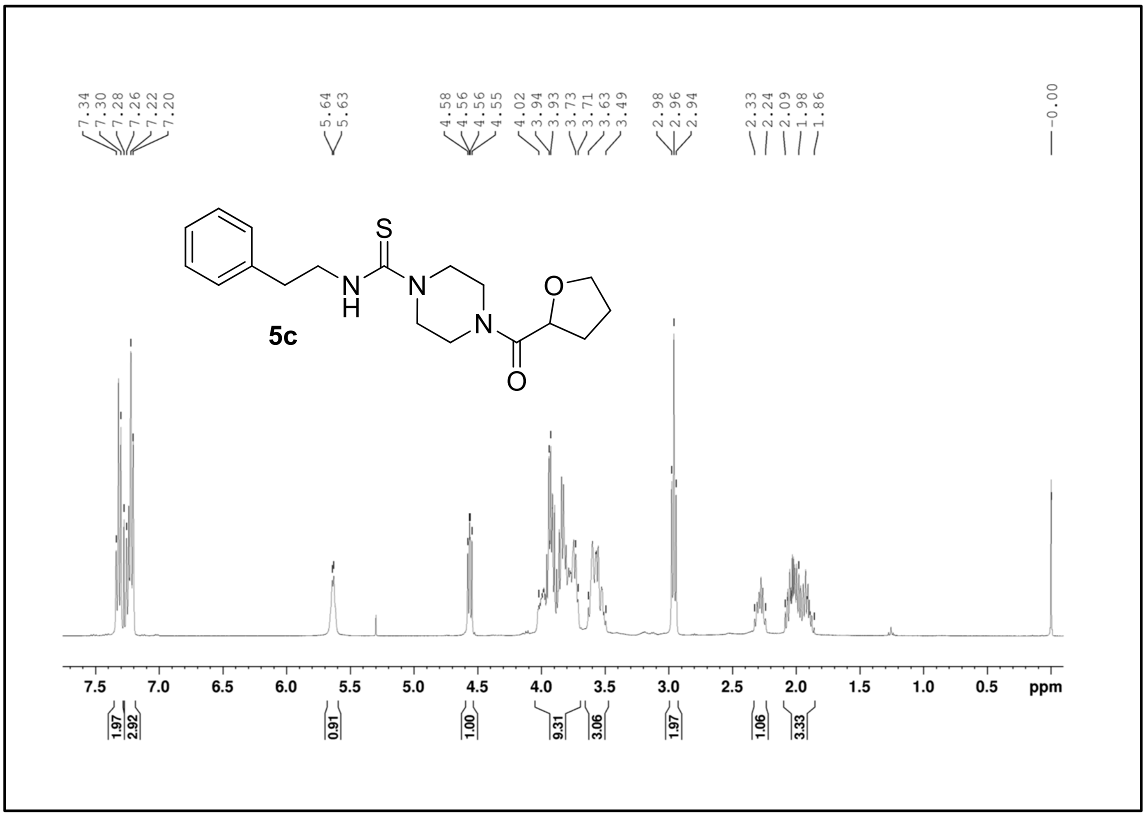

(A)

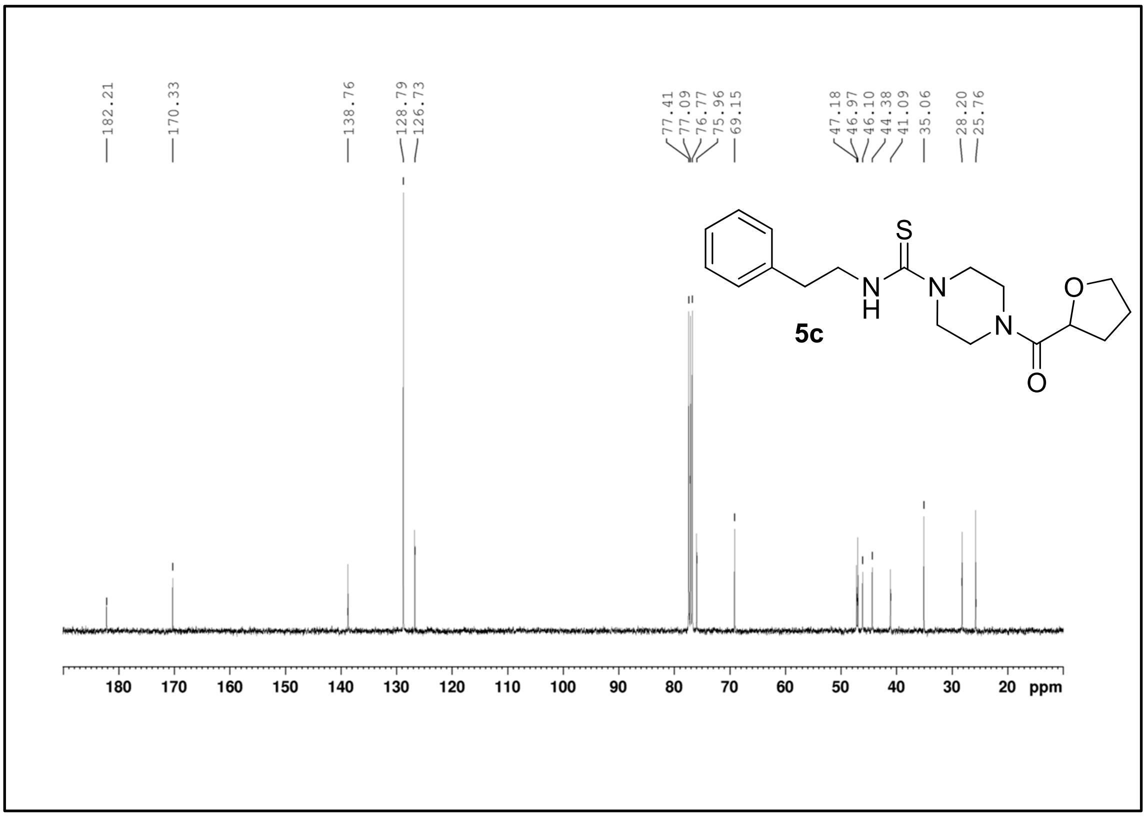

(B)

Figure S27. ^{1}H-NMR spectrum (A) and ^{13}C-NMR spectrum (B) of thiourea **5c**.

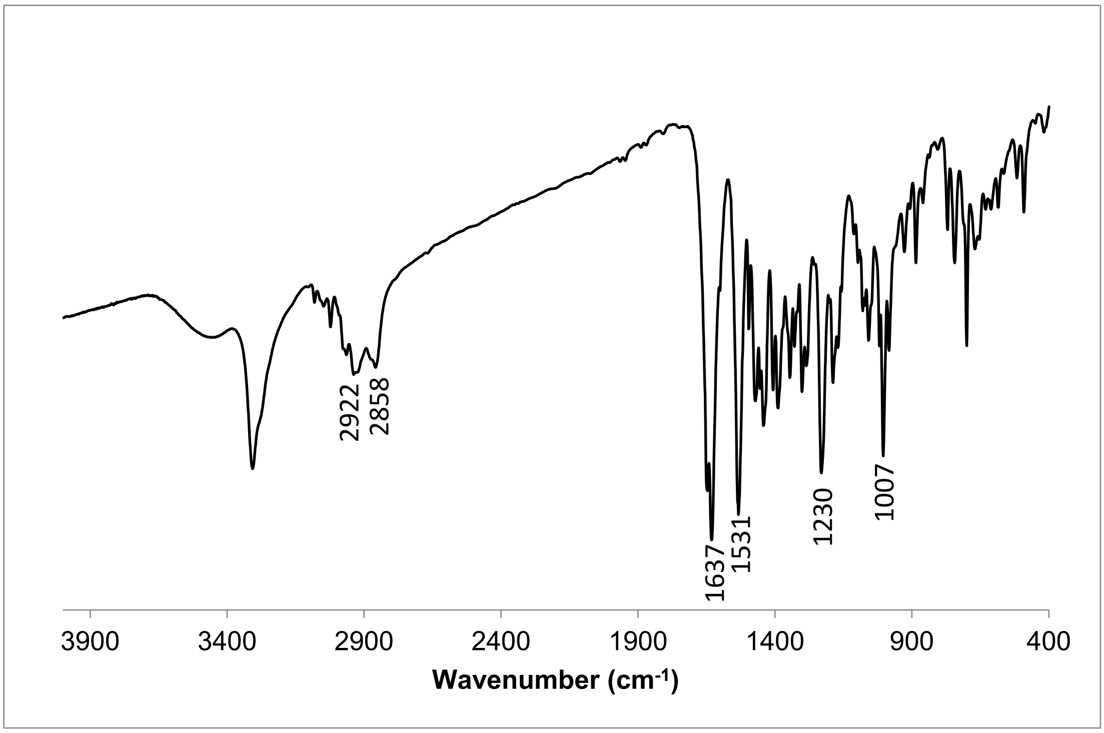

Figure S28. FT-IR spectrum of thiourea **5c**.

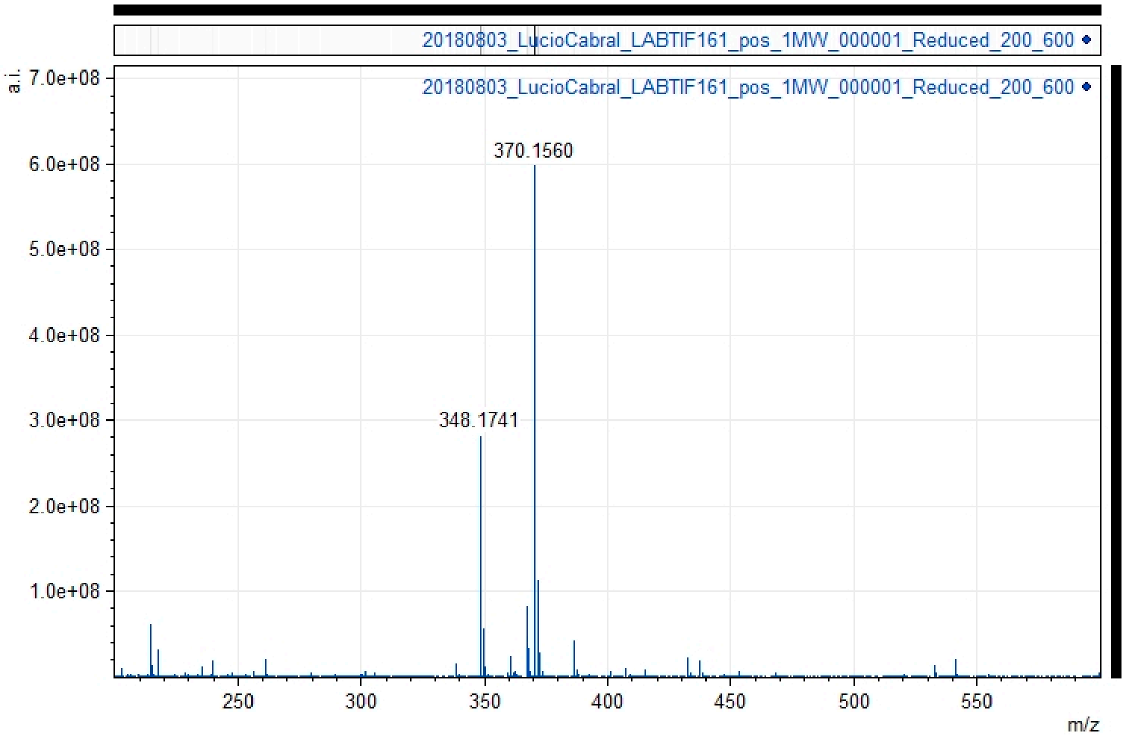

Figure S29. HR-MS spectrum of thiourea **5c**.

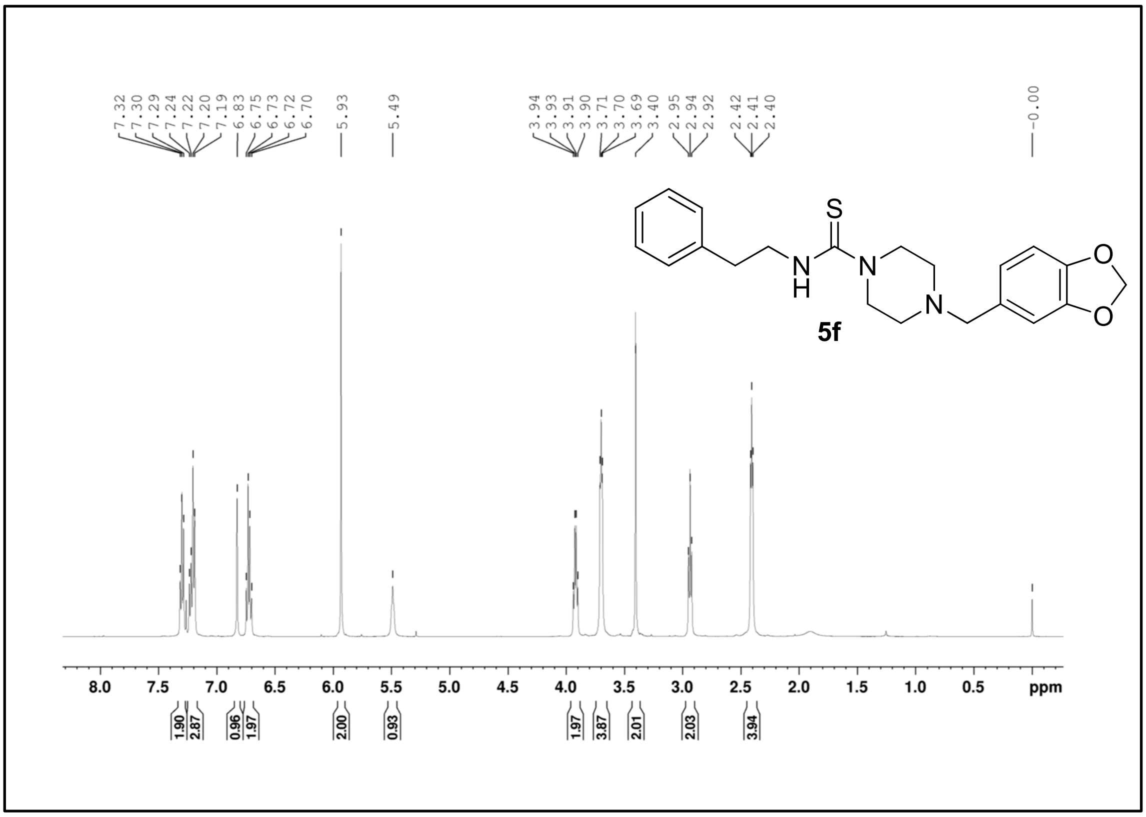

(A)

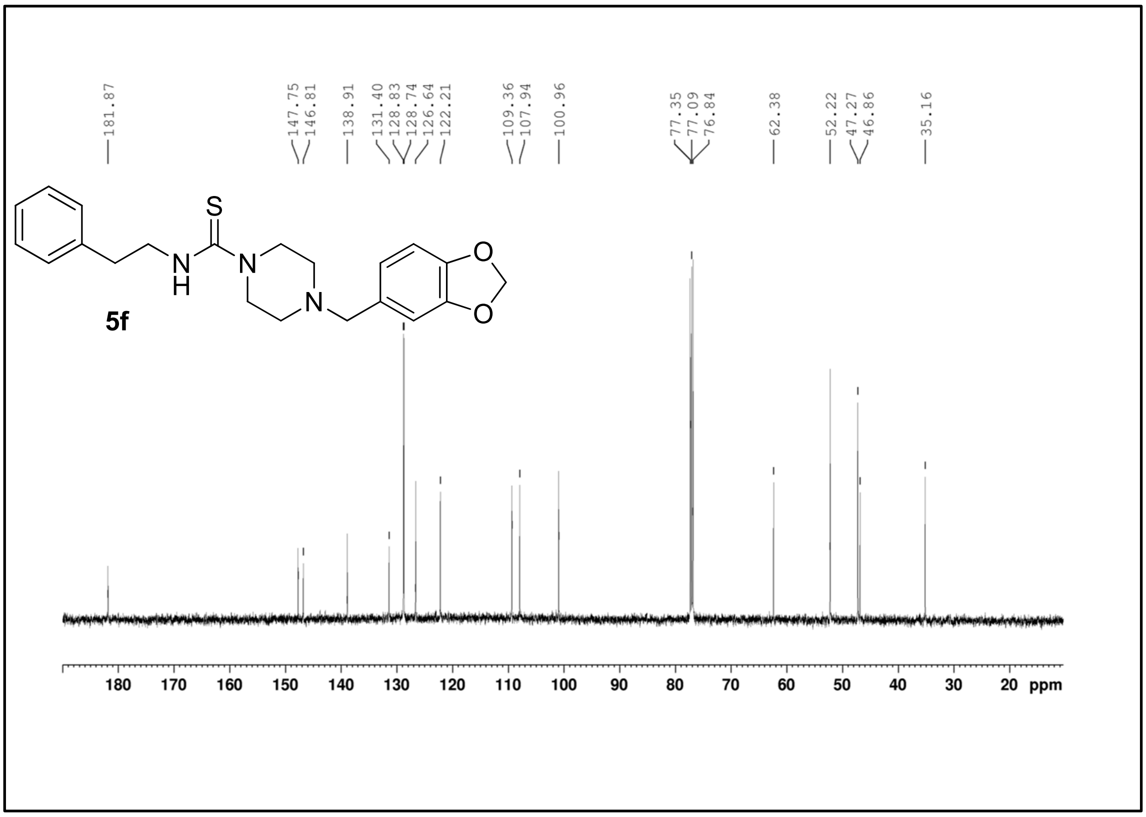

(B)

Figure S30. ¹H-NMR spectrum (A) and ¹³C-NMR spectrum (B) of thiourea **5f**.

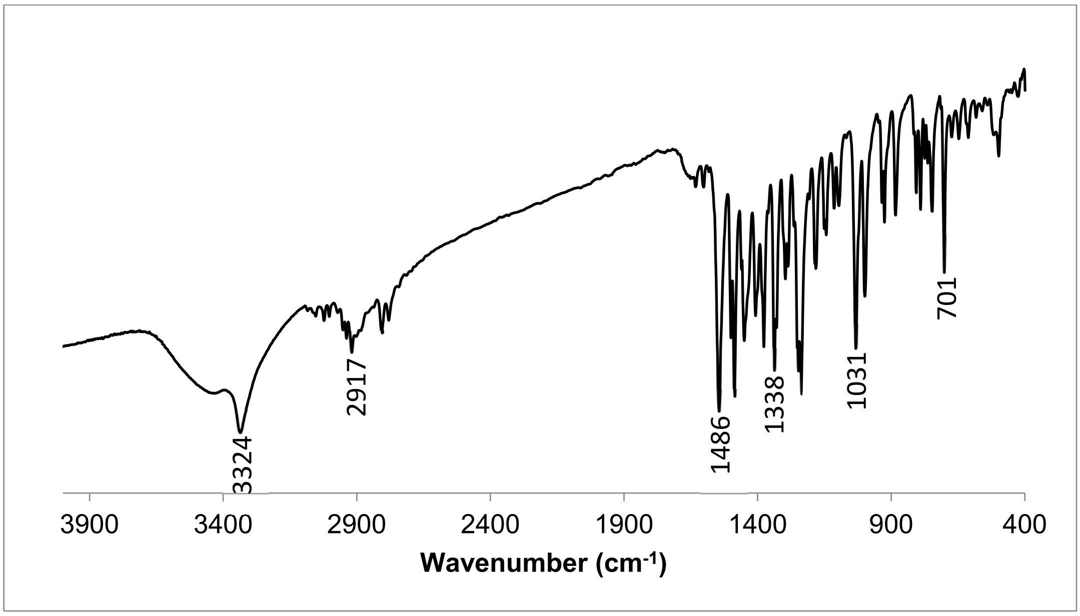

Figure S31. FT-IR spectrum of thiourea **5f**.

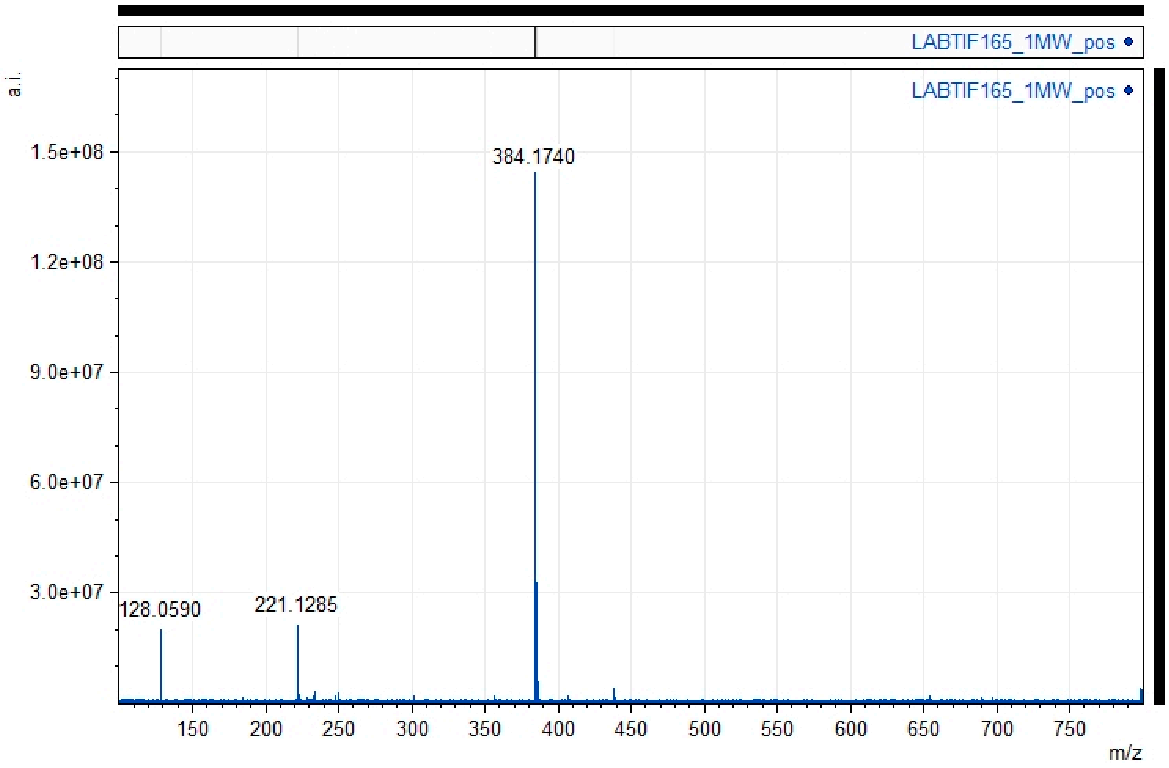

Figure S32. HR-MS spectrum of thiourea **5f**.

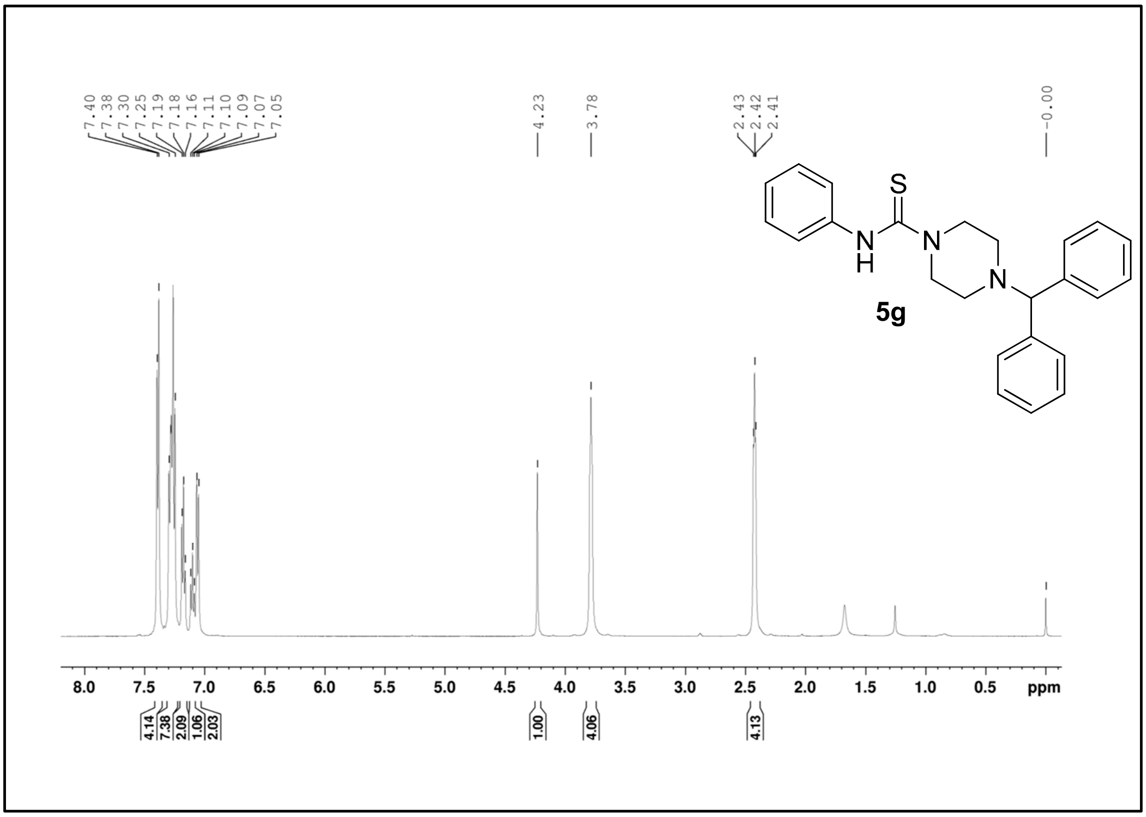

(A)

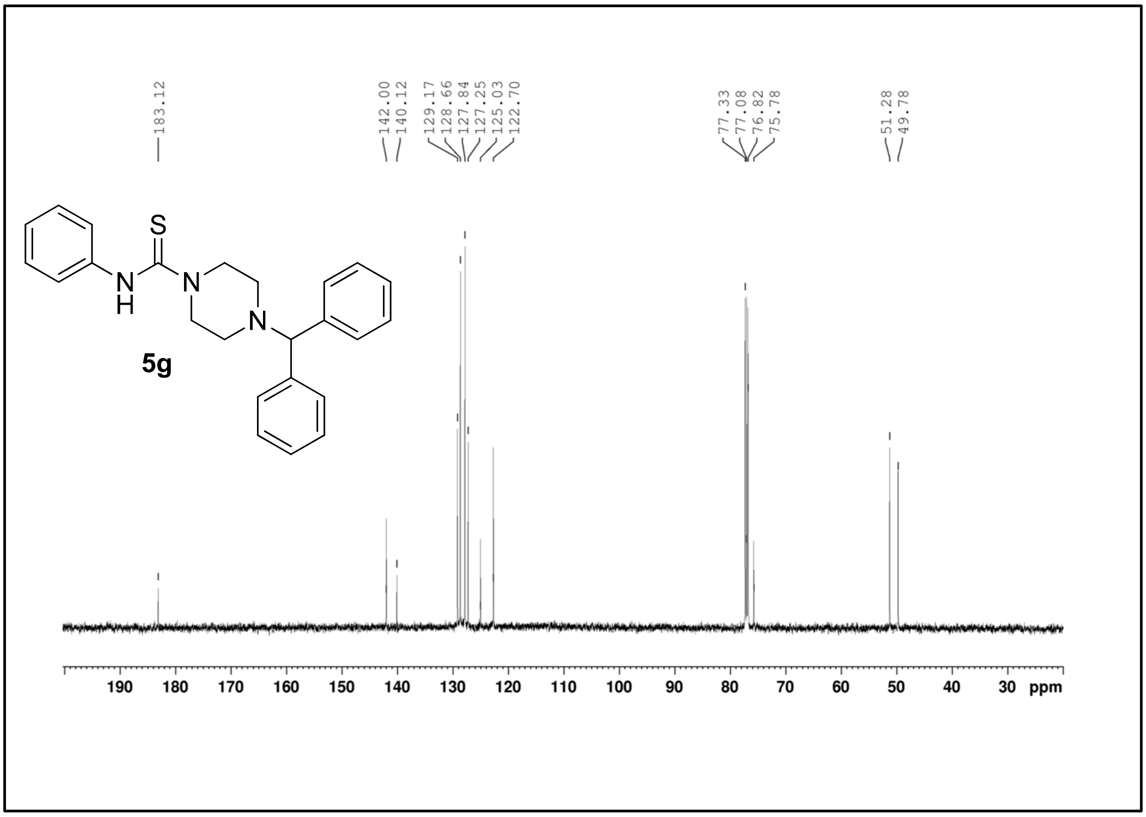

(B)

Figure S33. ^{1}H-NMR spectrum (A) and ^{13}C-NMR spectrum (B) of thiourea **5g**.

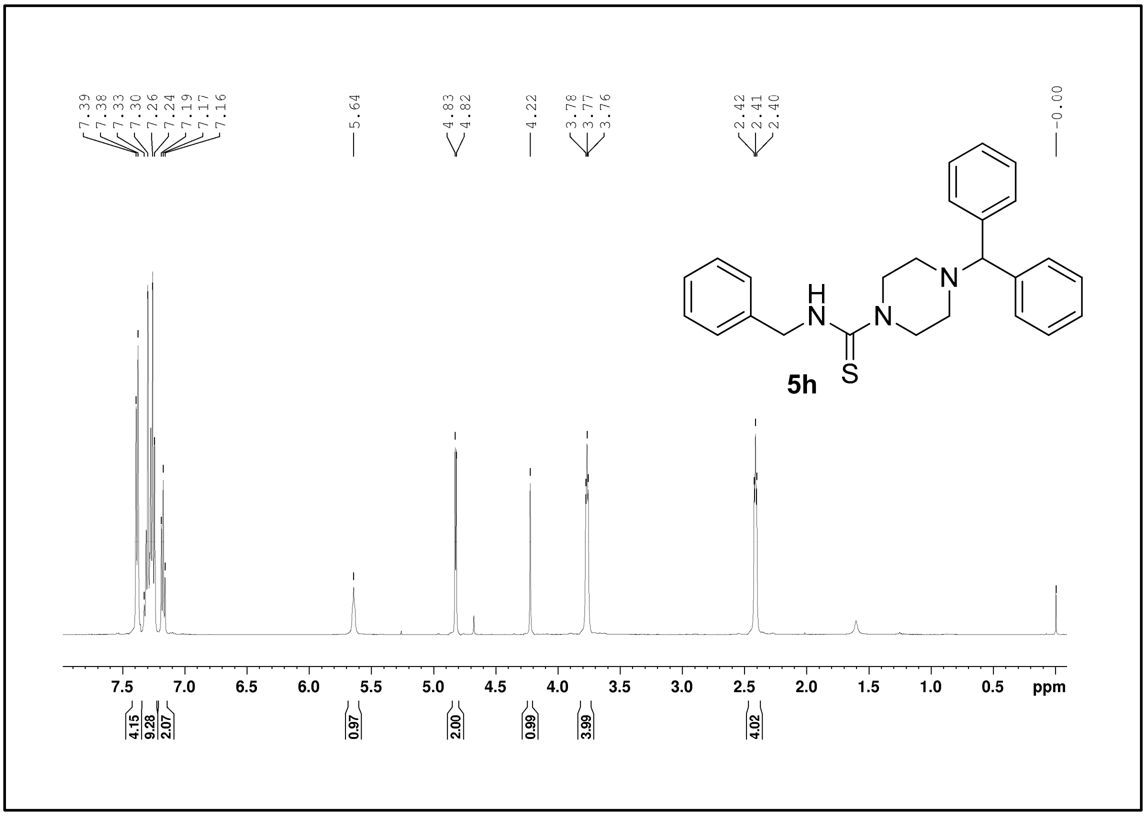

(A)

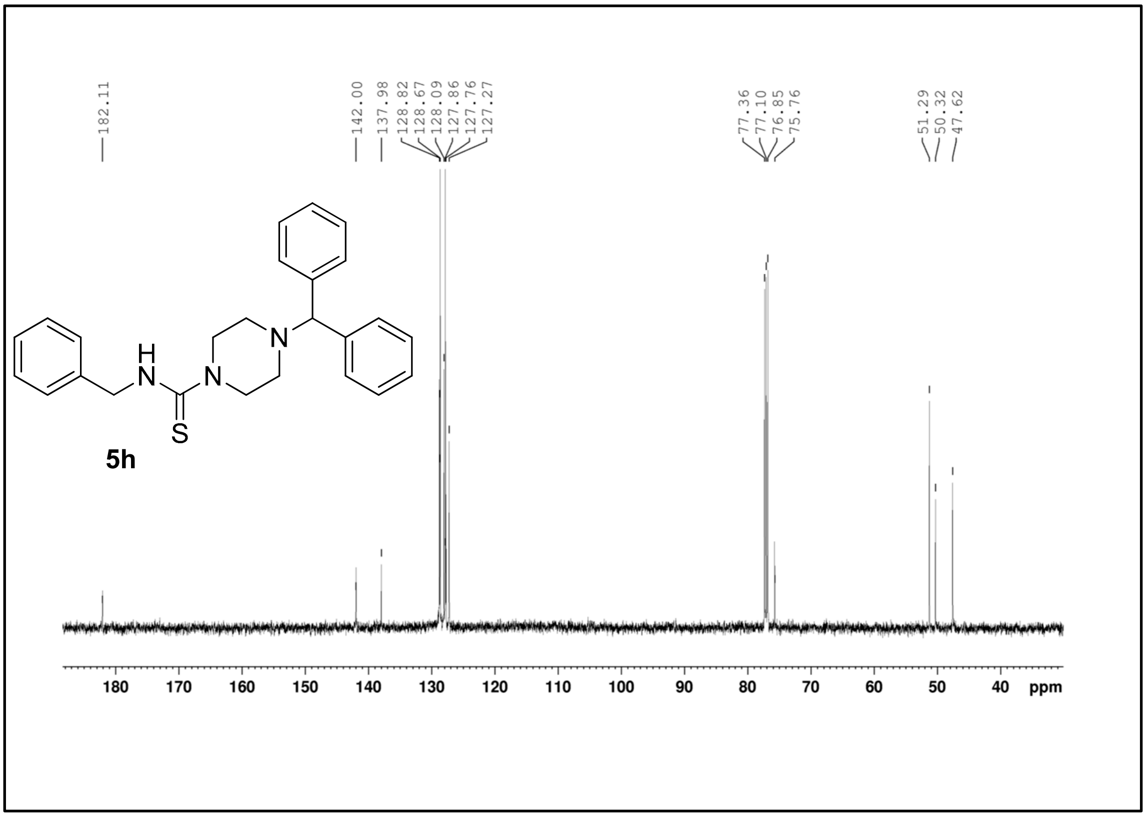

(B)

Figure S34. ^{1}H-NMR spectrum (A) and ^{13}C-NMR spectrum (B) of thiourea **5h**.

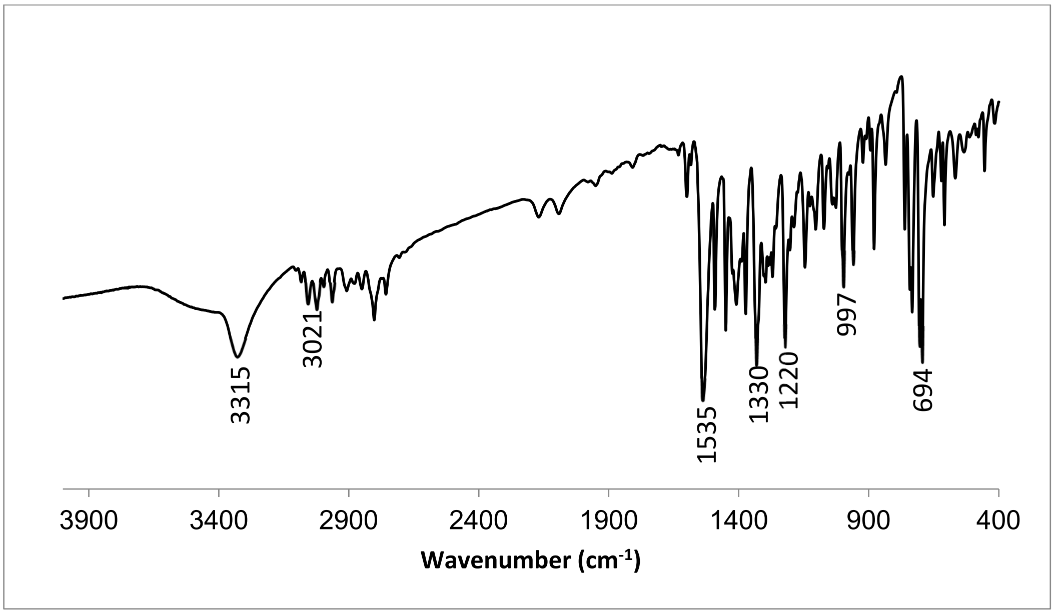

Figure S35. FT-IR spectrum of thiourea **5h**.

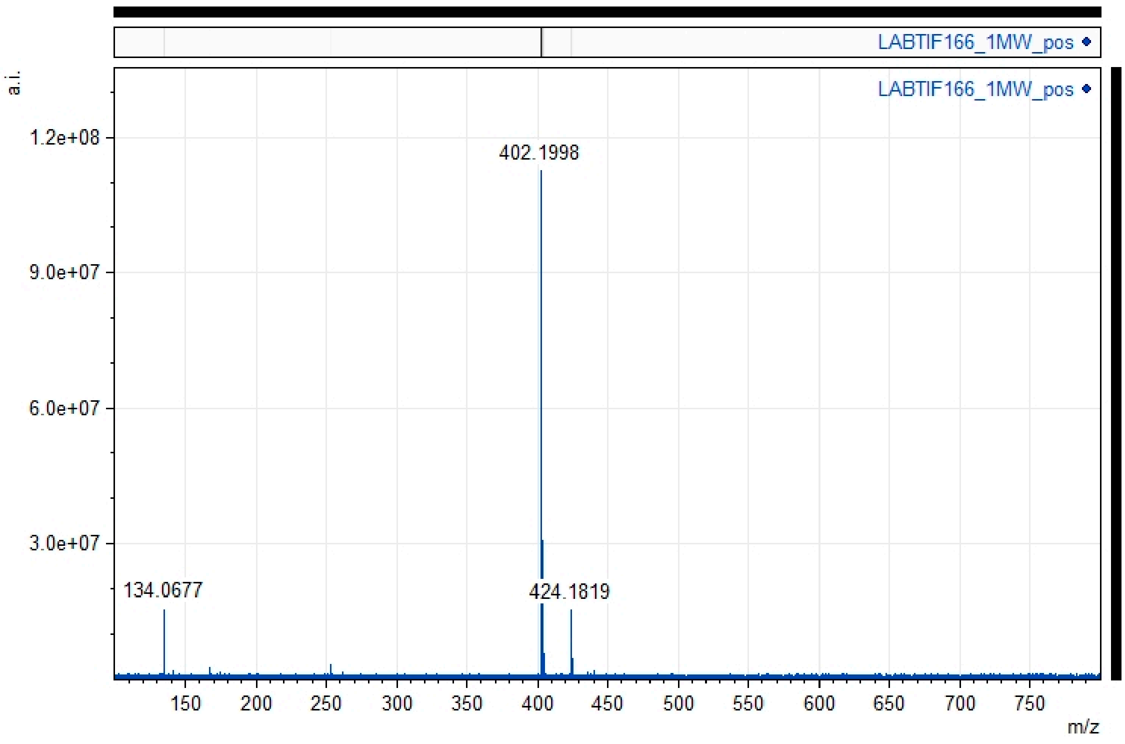

Figure S36. HR-MS spectrum of thiourea **5h**.

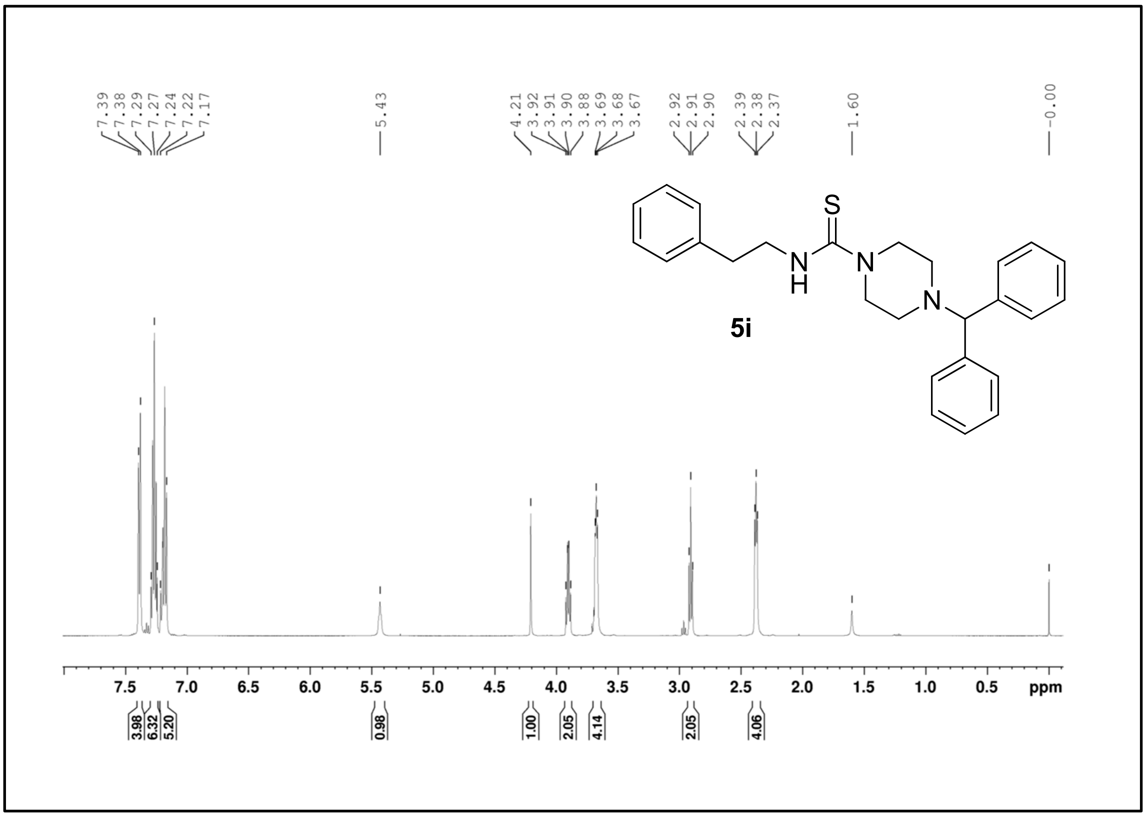

(A)

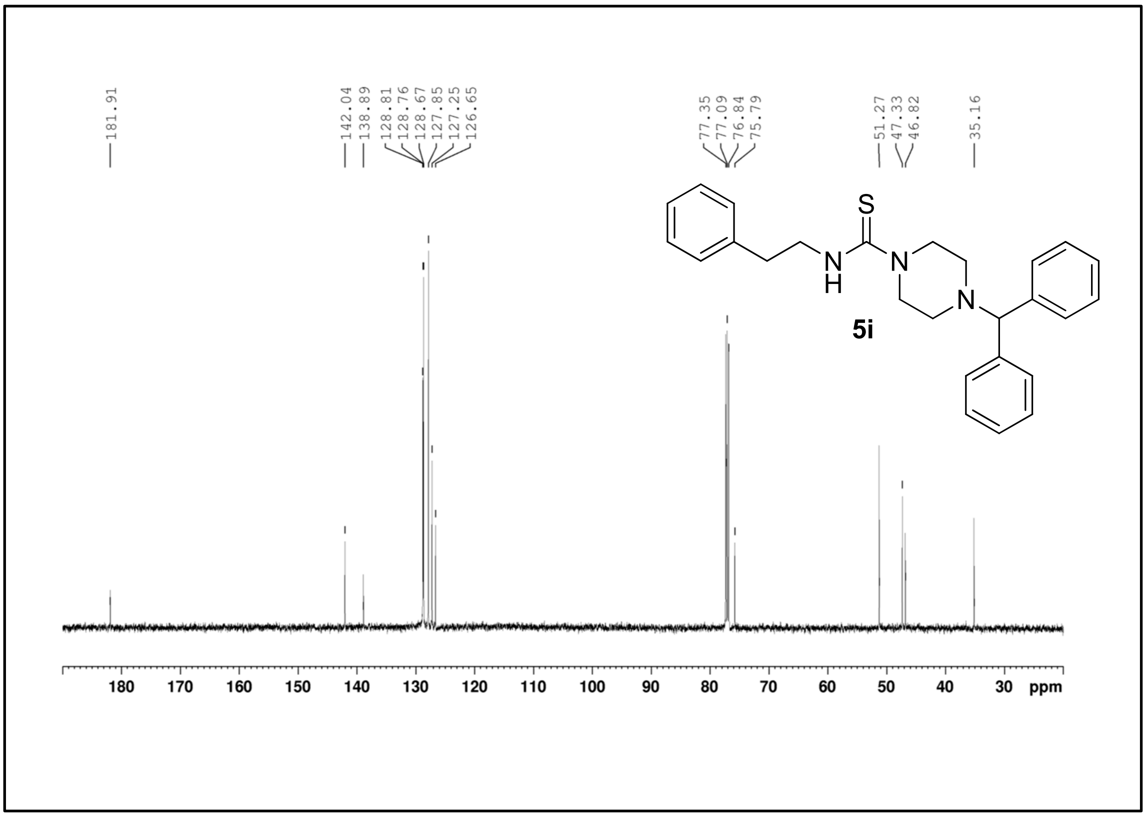

(B)

Figure S37. ^{1}H-NMR spectrum (A) and ^{13}C-NMR spectrum (B) of thiourea **5i**.

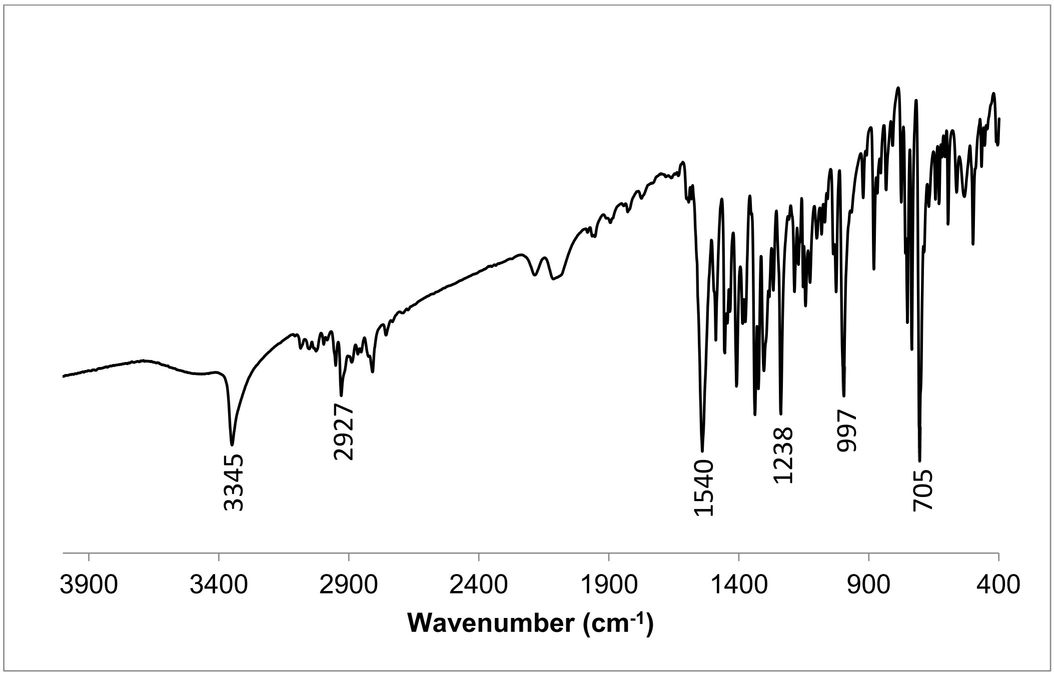

Figure S38. FT-IR spectrum of thiourea **5i**.

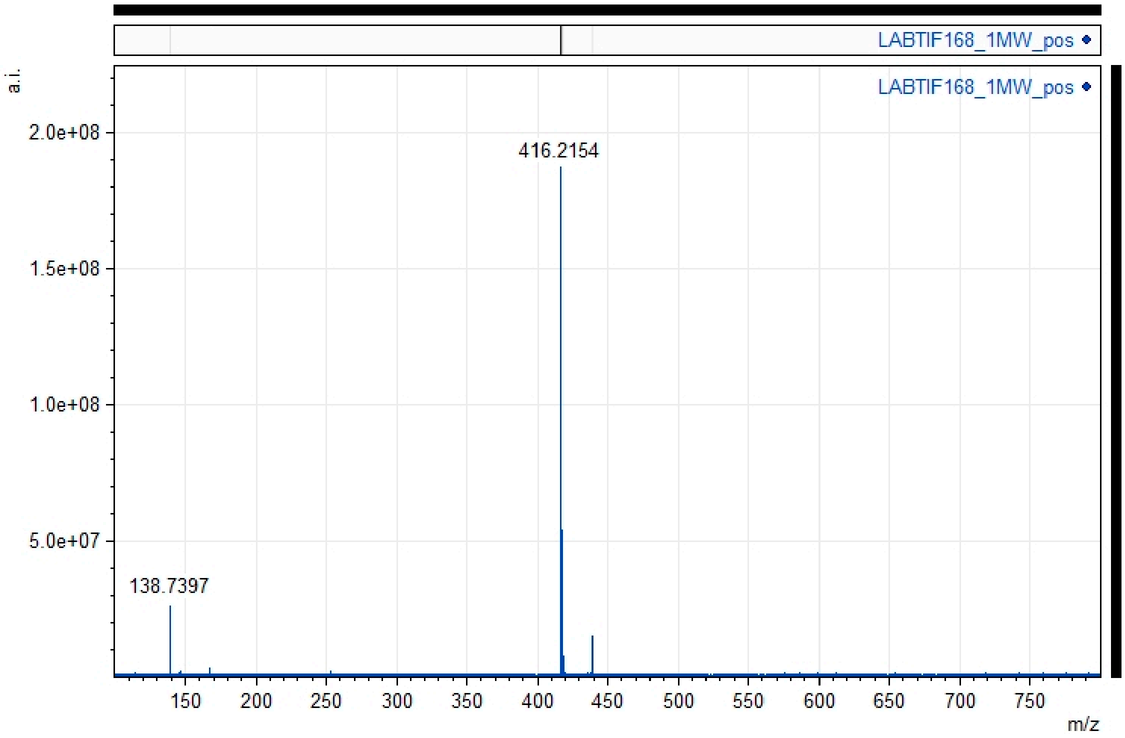

Figure S39. HR-MS spectrum of thiourea **5i**.

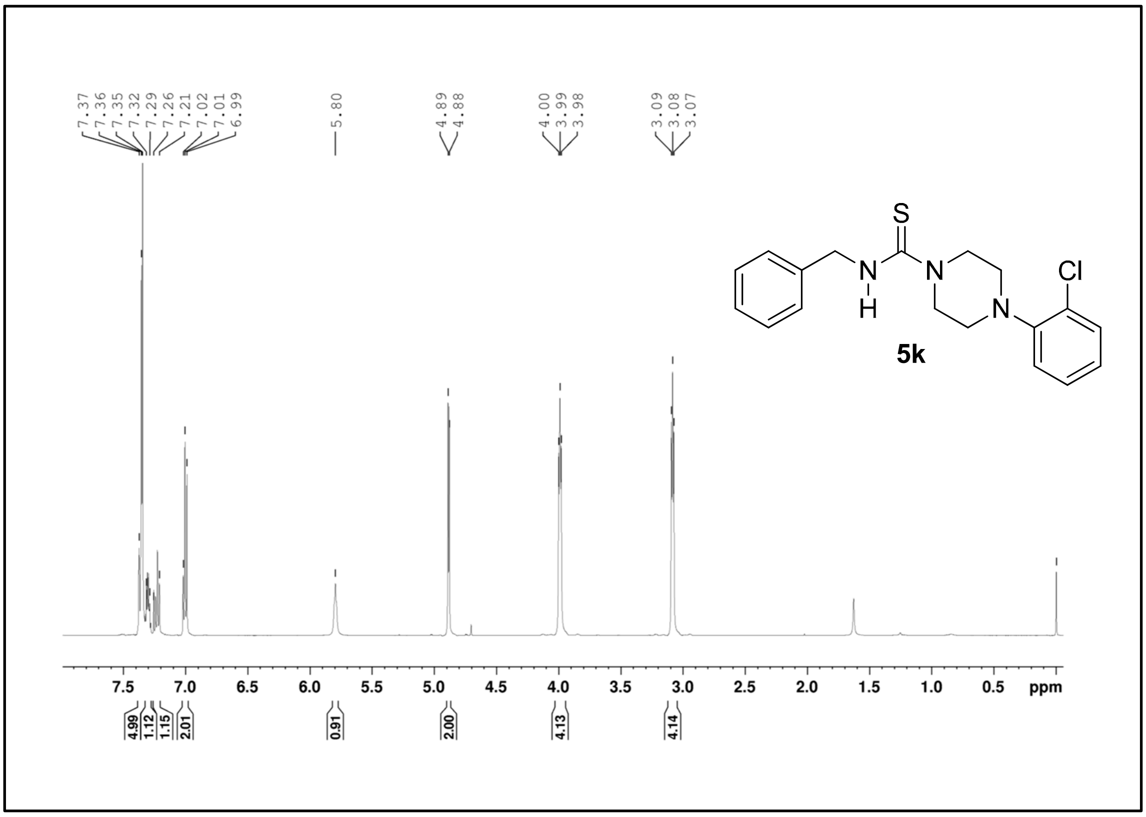

(A)

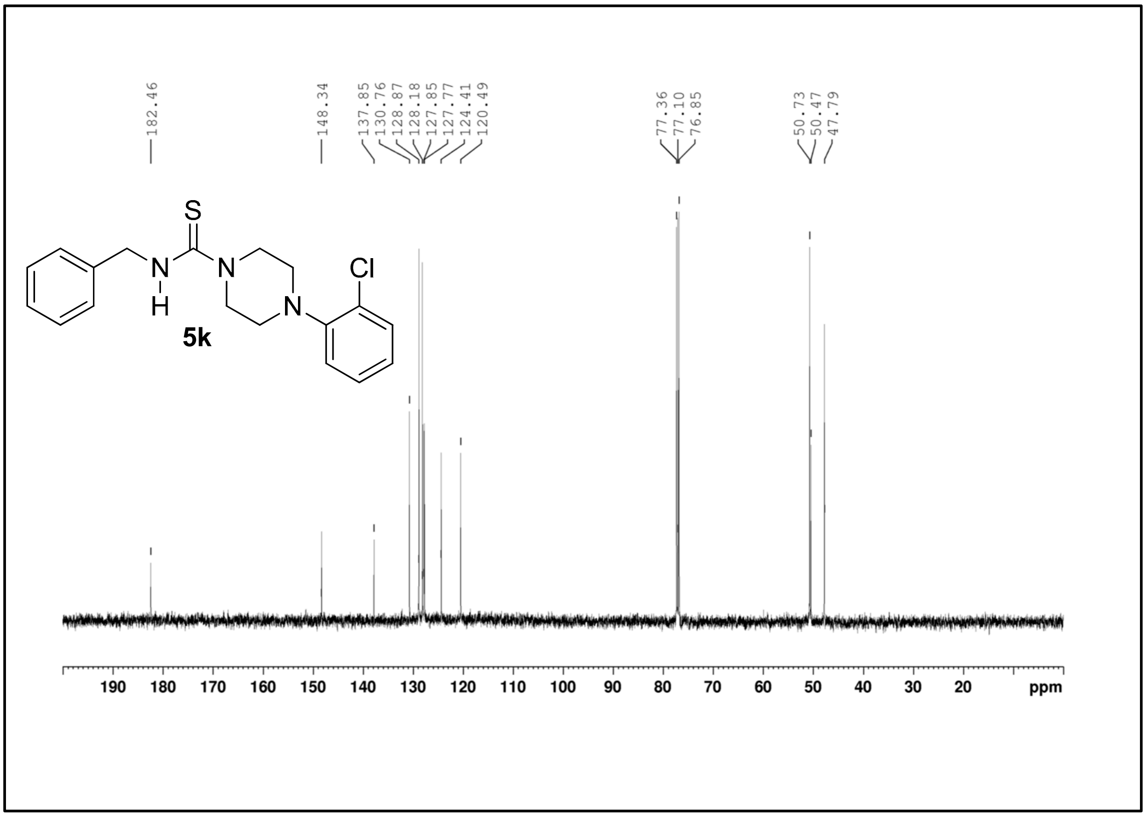

(B)

Figure S40. ^{1}H-NMR spectrum (A) and ^{13}C-NMR spectrum (B) of thiourea **5k**.

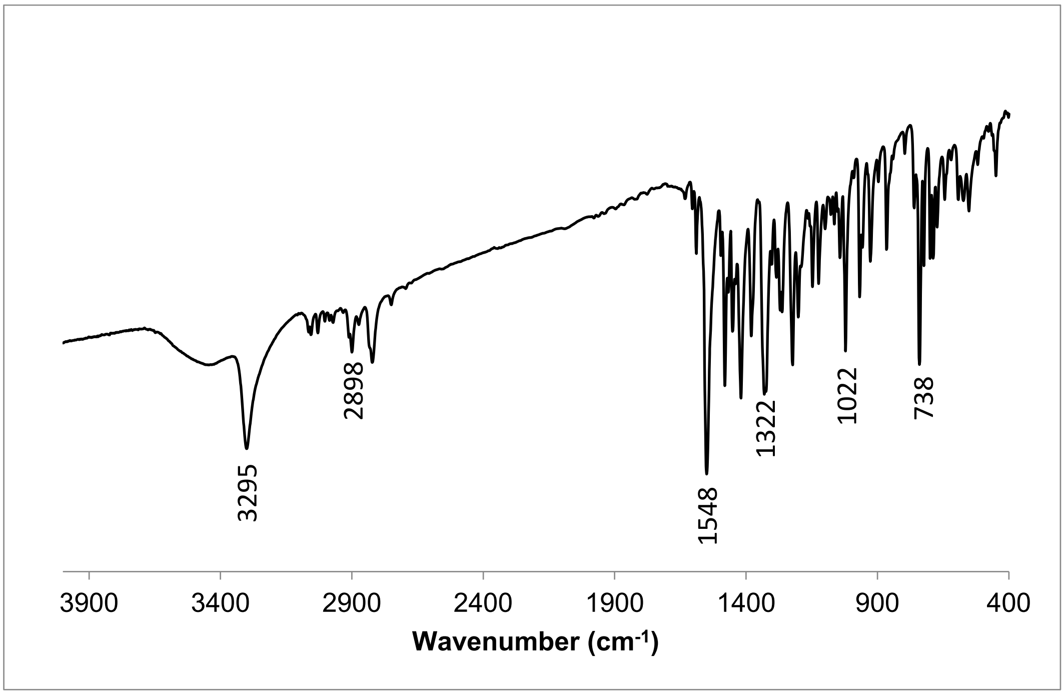

Figure S41. FT-IR spectrum of thiourea **5k**.

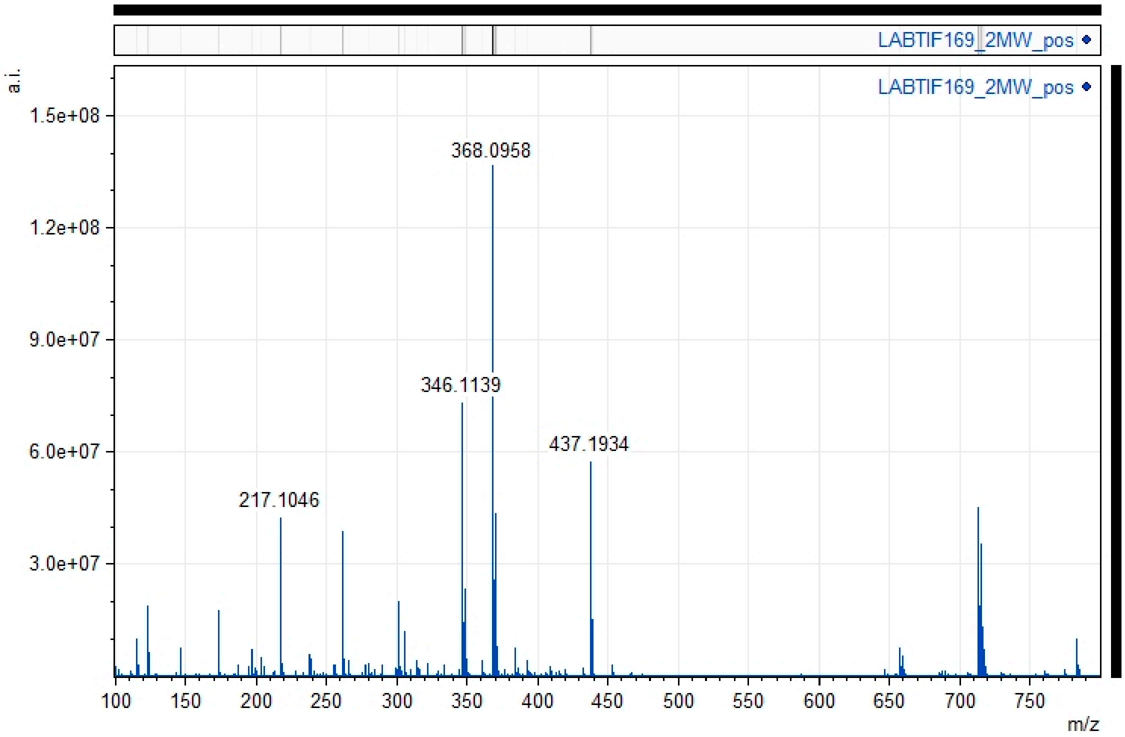

Figure S42. HR-MS spectrum of thiourea **5k**.

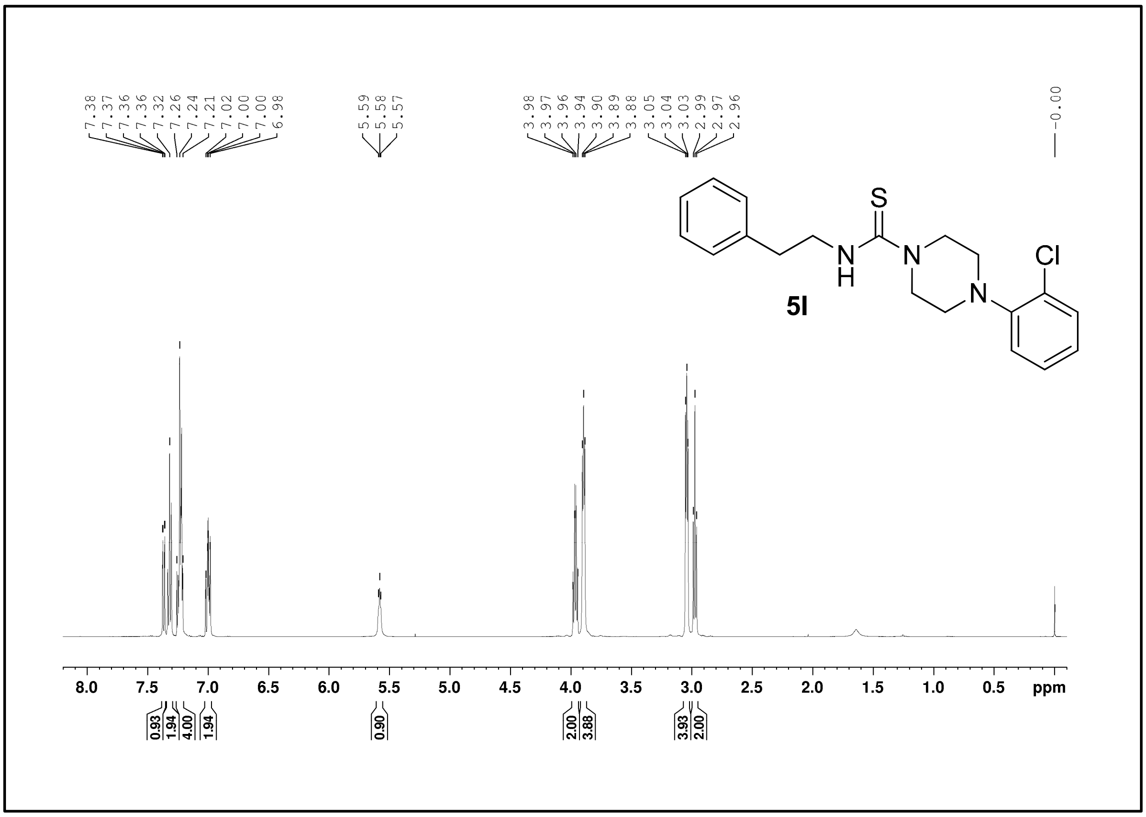

(A)

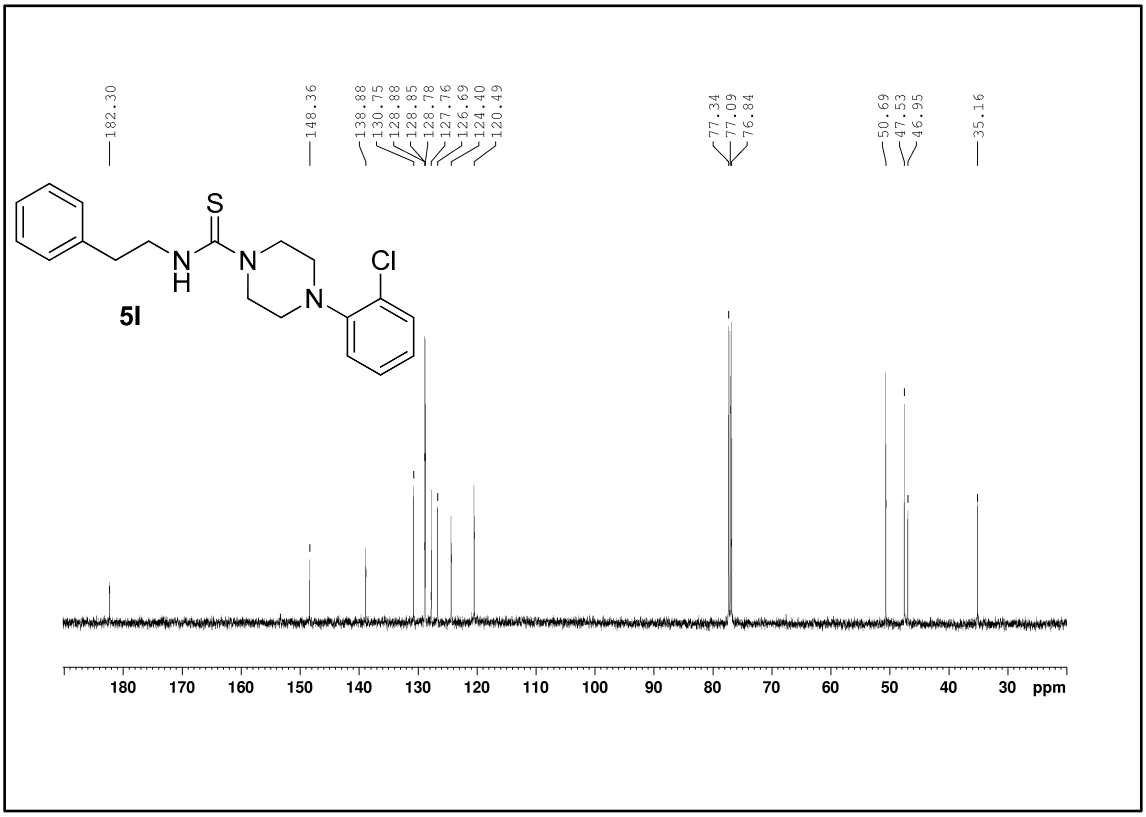

(B)

Figure S43. ^{1}H-NMR spectrum (A) and ^{13}C-NMR spectrum (B) of thiourea **5l**.

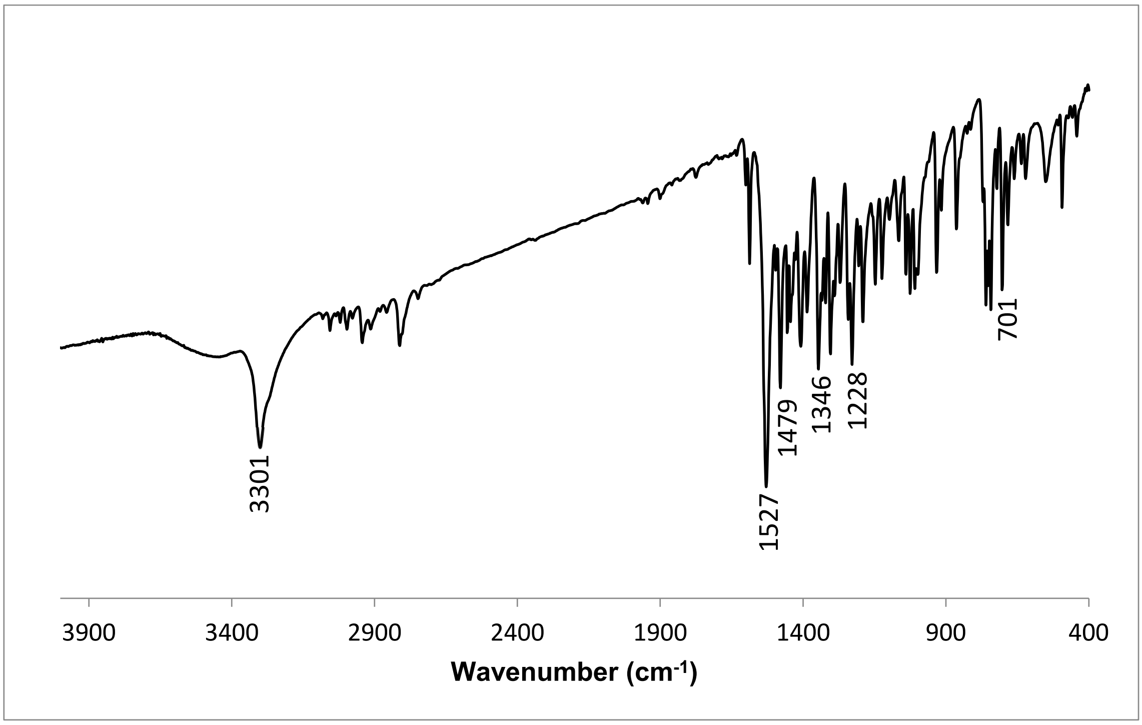

Figure S44. FT-IR spectrum of thiourea **5l**.

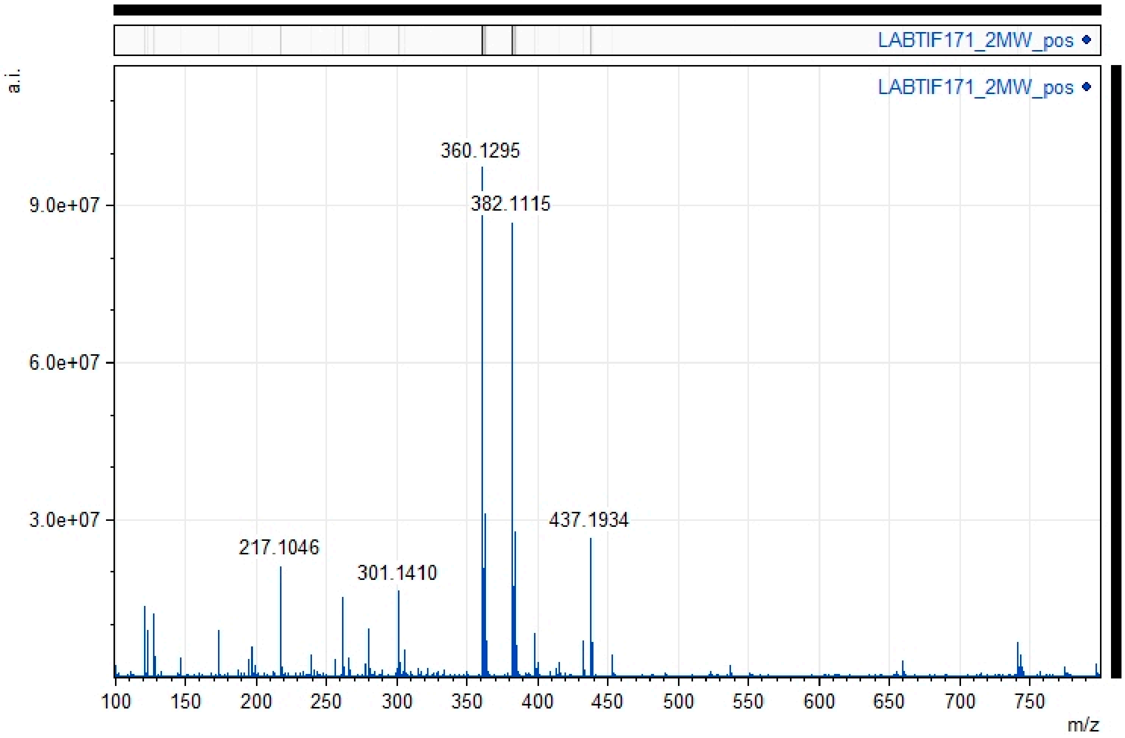

Figure S45. HR-MS spectrum of thiourea **5l**.

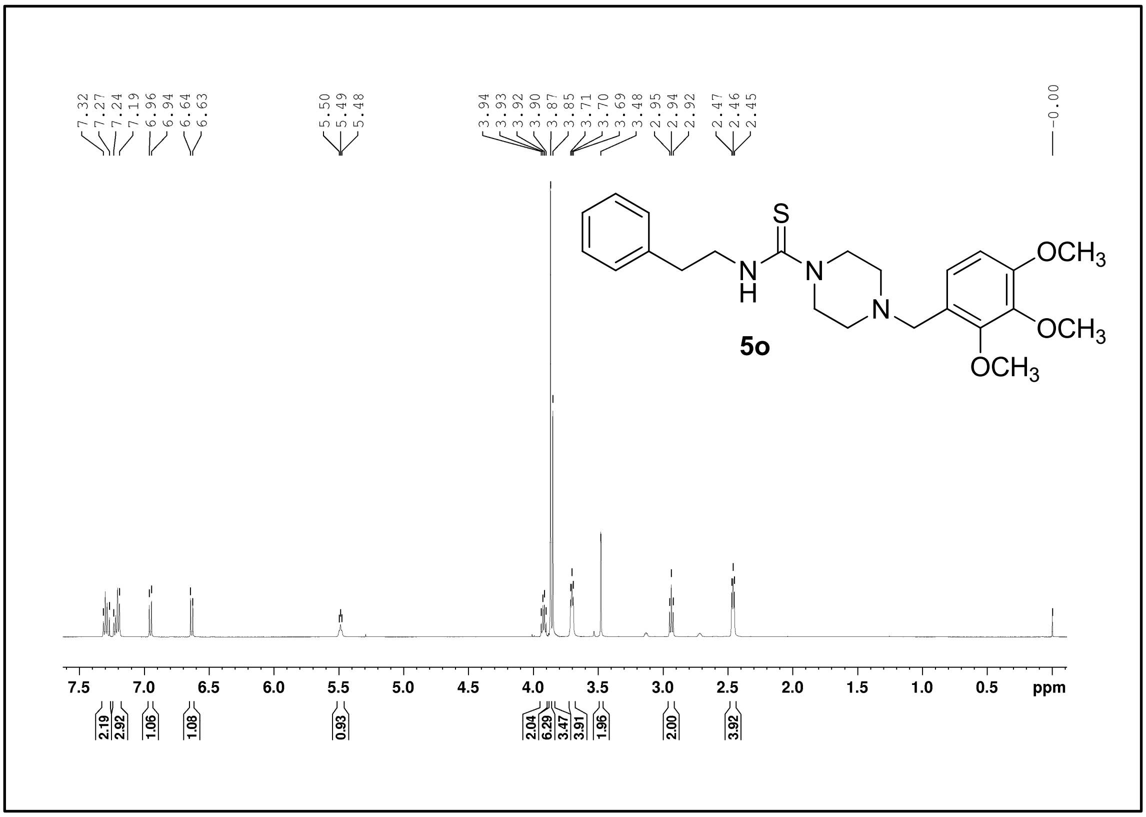

(A)

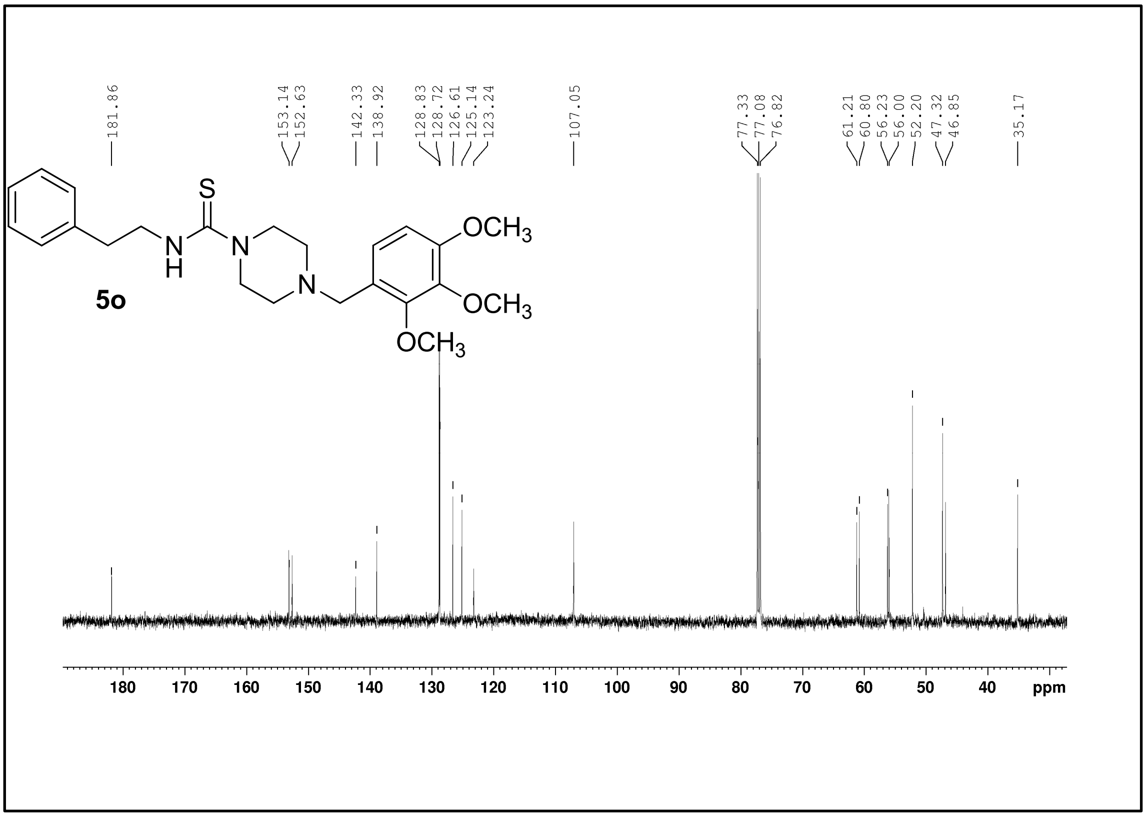

(B)

Figure S46. ^{1}H-NMR spectrum (A) and ^{13}C-NMR spectrum (B) of thiourea **5o**.

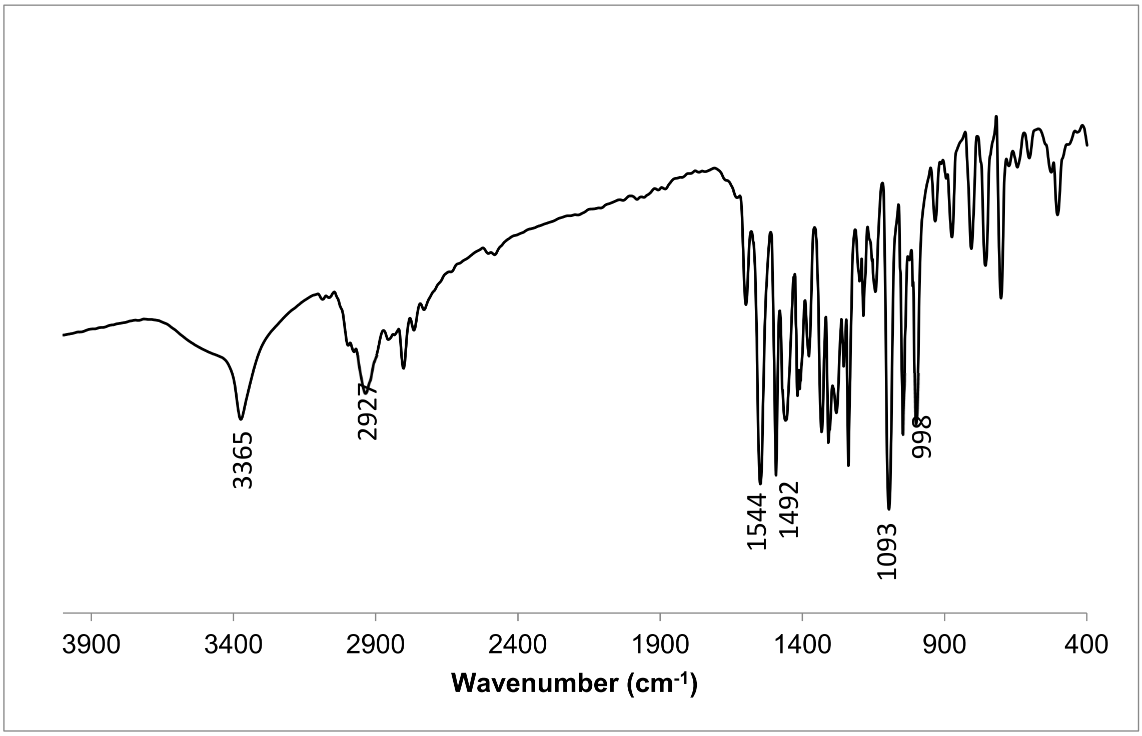

Figure S47. FT-IR spectrum of thiourea **5o**.

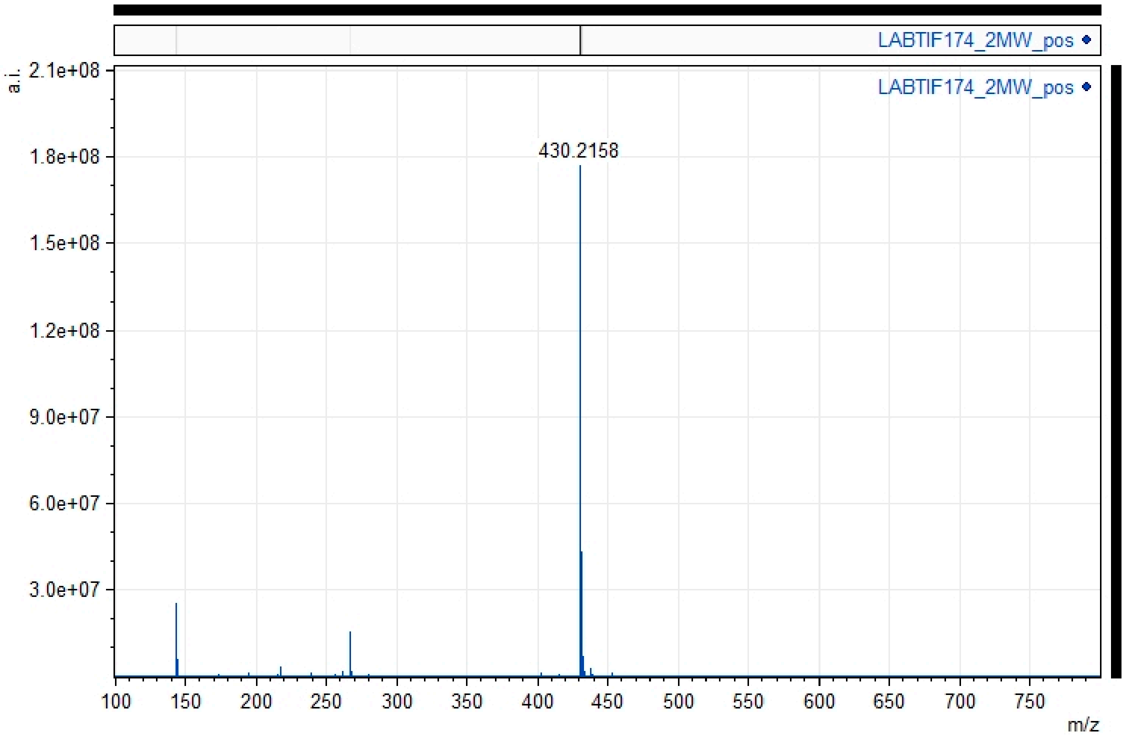

Figure S48. HR-MS spectrum of thiourea **5o**.

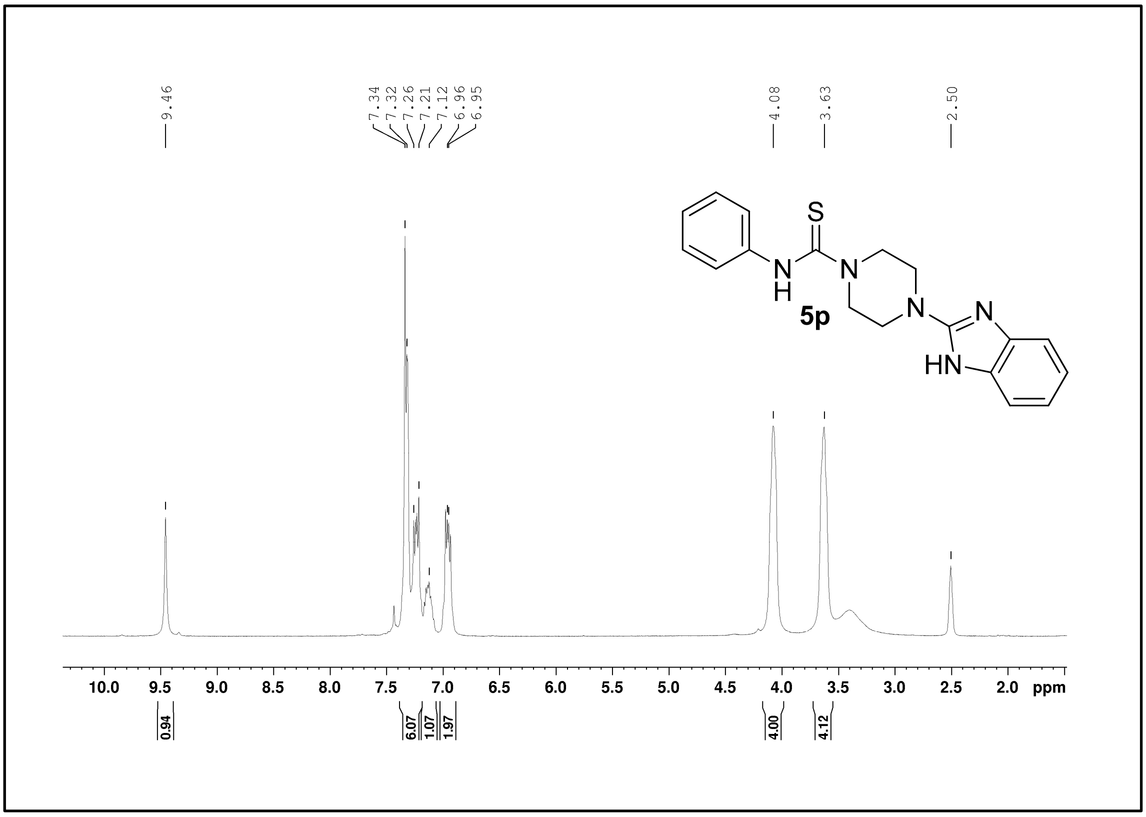
9.46
7.34
7.32
7.26
7.21
7.12
6.96
6.95
4.08
3.63
2.50
5p
10.0 9.5 9.0 8.5 8.0 7.5 7.0 6.5 6.0 5.5 5.0 4.5 4.0 3.5 3.0 2.5 2.0 ppm
0.94
6.07
1.07
1.97
4.00
4.12

(A)

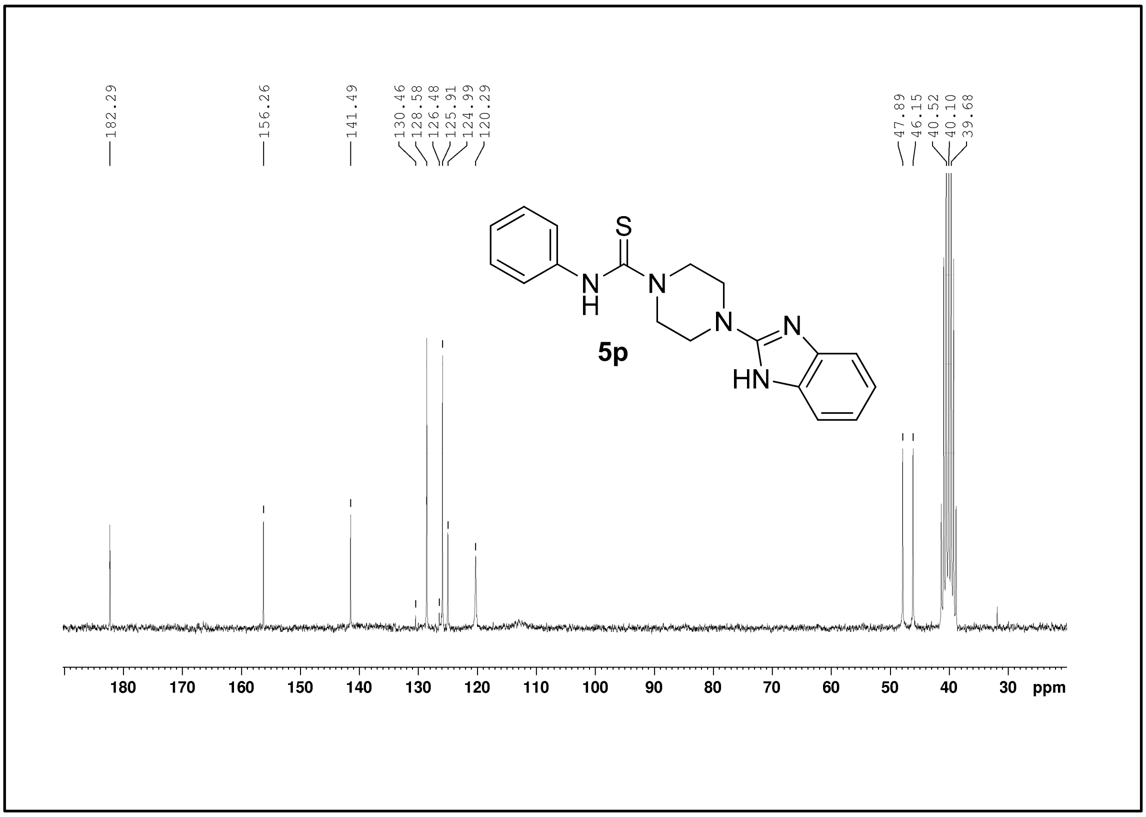
182.29
156.26
141.49
130.46
128.58
126.48
125.91
124.99
120.29
47.89
46.15
40.52
40.10
39.68
5p
180 170 160 150 140 130 120 110 100 90 80 70 60 50 40 30 ppm

(B)

Figure S49. ^{1}H-NMR spectrum (A) and ^{13}C-NMR spectrum (B) of thiourea **5p**.

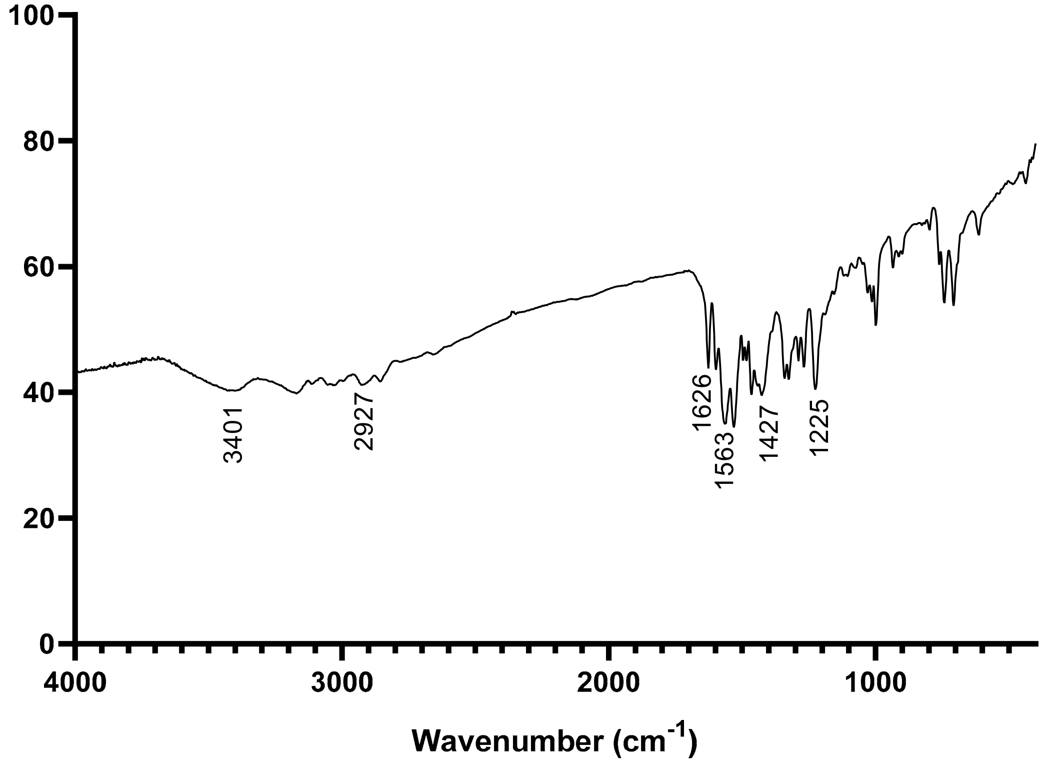

Figure S50. FT-IR spectrum of thiourea **5p**.

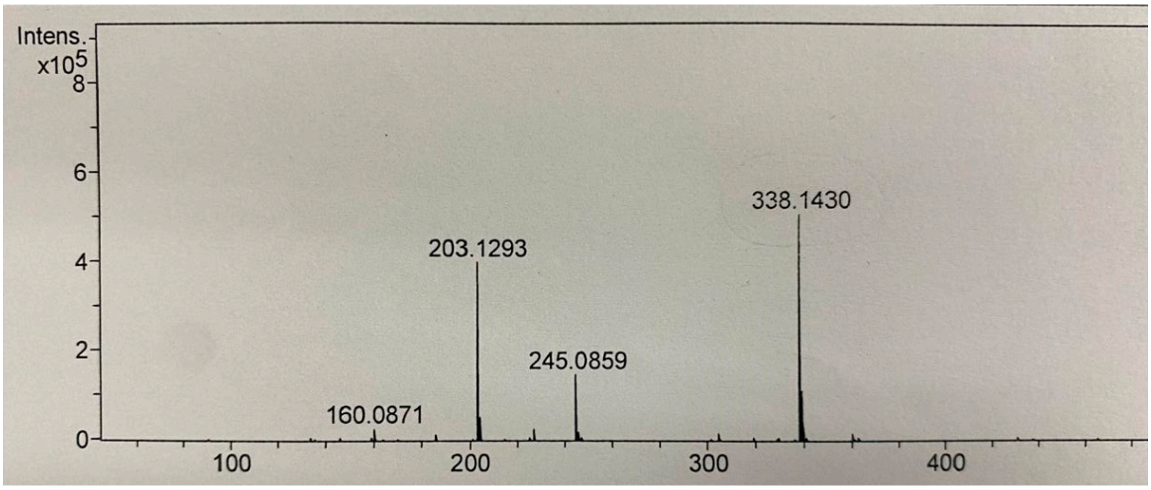

Figure S51. HR-MS spectrum of thiourea **5p**.

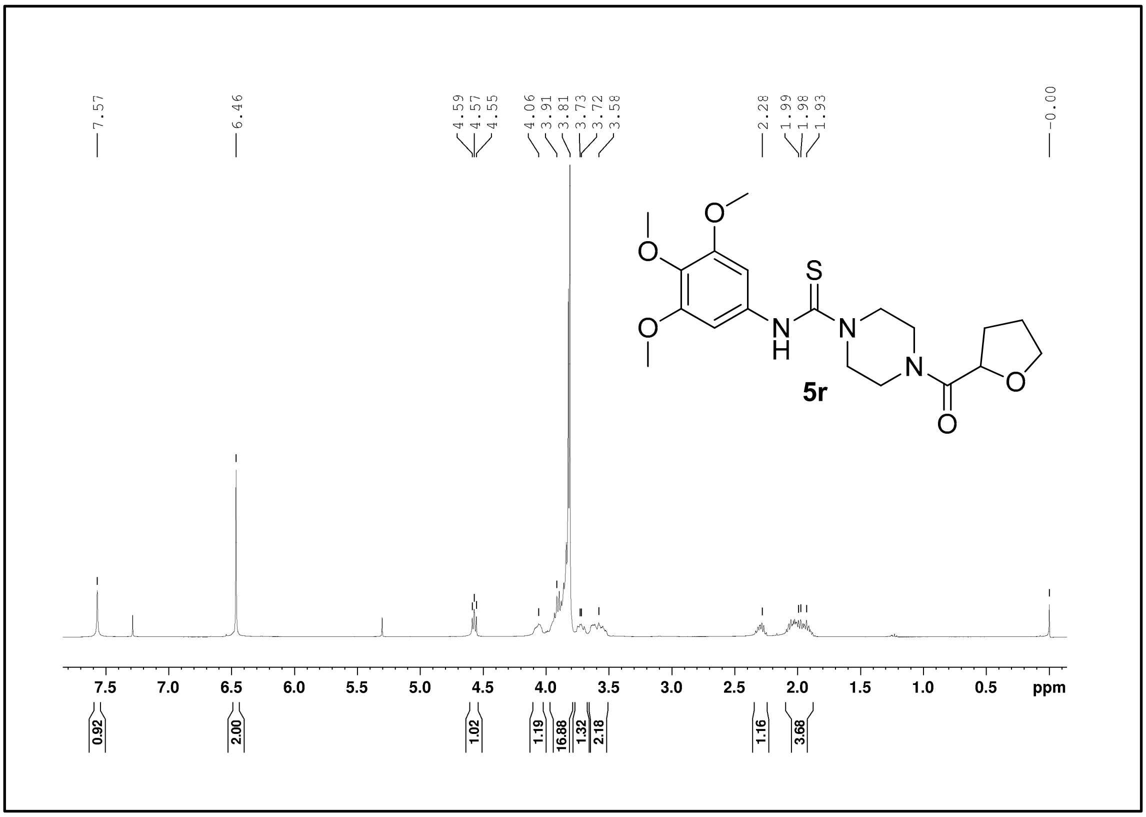

(A)

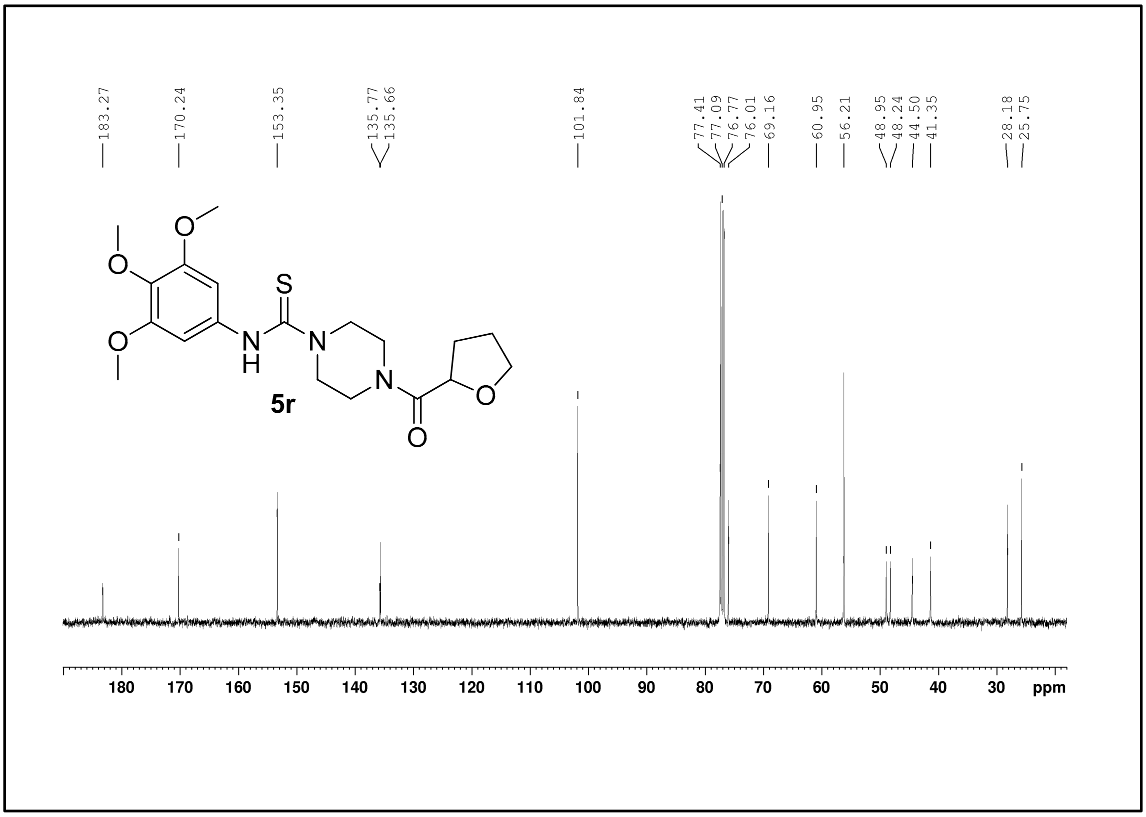

(B)

Figure S52. ^{1}H-NMR spectrum (A) and ^{13}C-NMR spectrum (B) of thiourea **5r**.

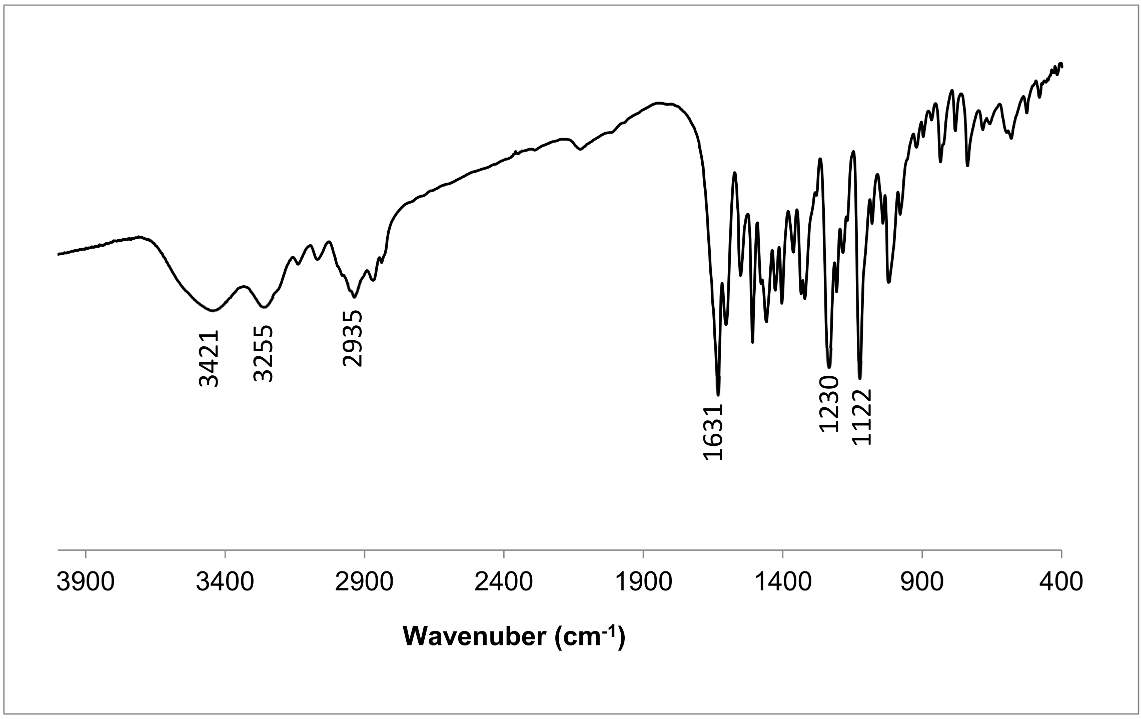

Figure S53. FT-IR spectrum of thiourea **5r**.

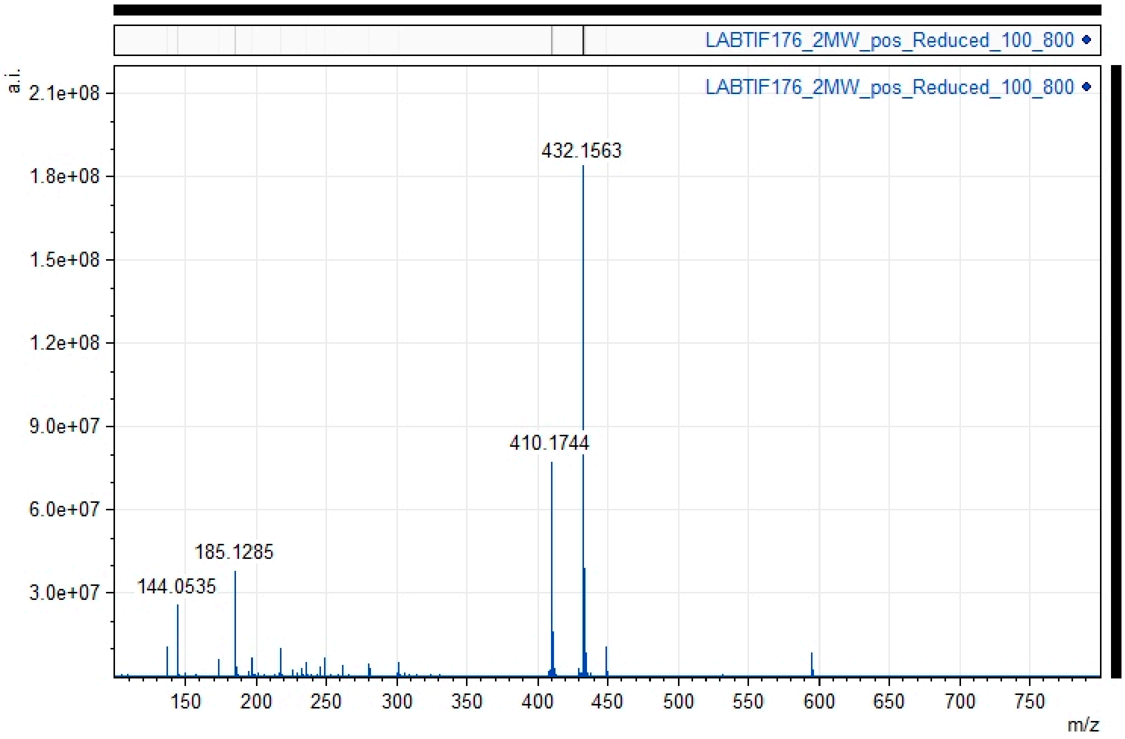

Figure S54. HR-MS spectrum of thiourea **5r**.

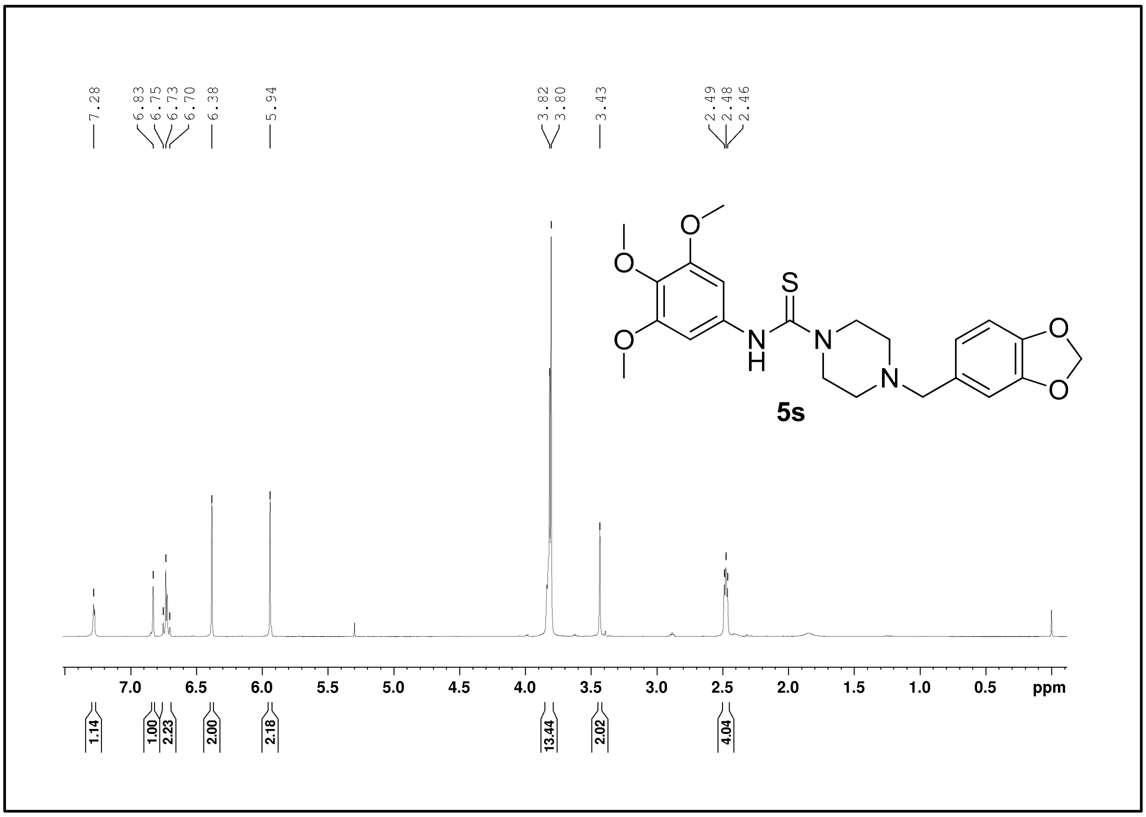

(A)

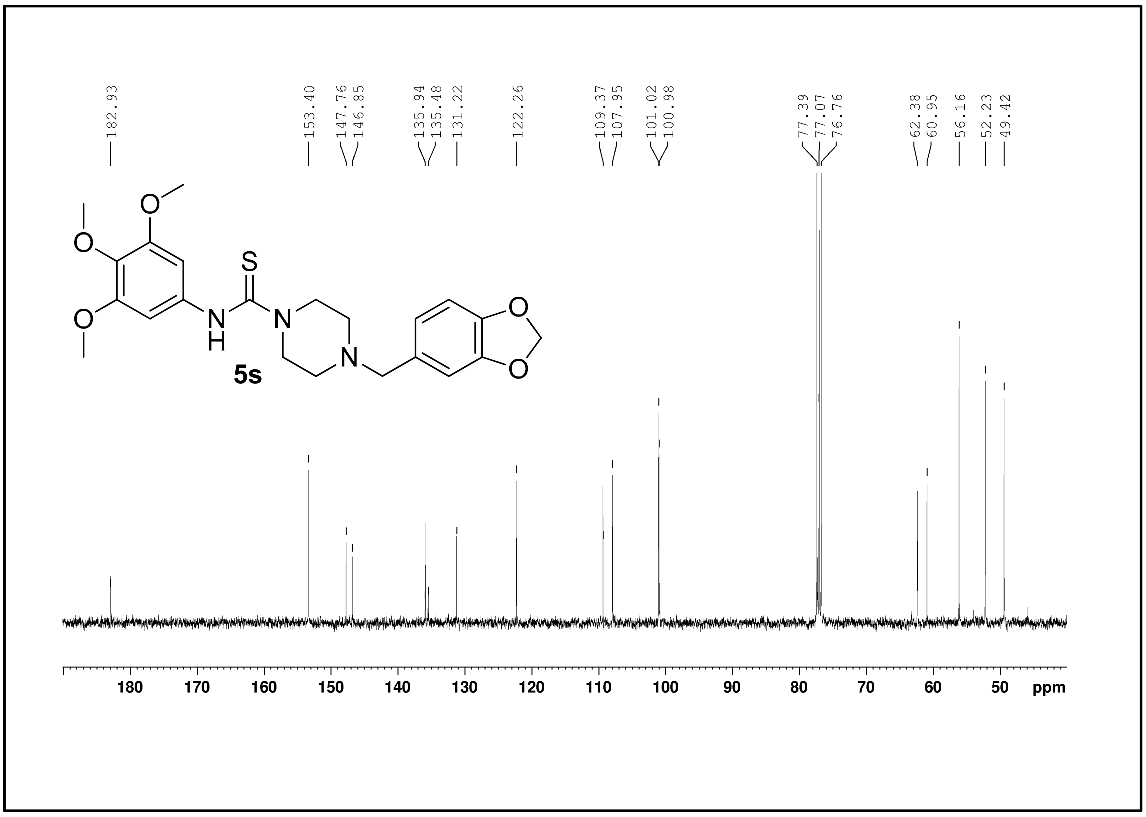

(B)

Figure S55. ^{1}H-NMR spectrum (A) and ^{13}C-NMR spectrum (B) of thiourea **5s**.

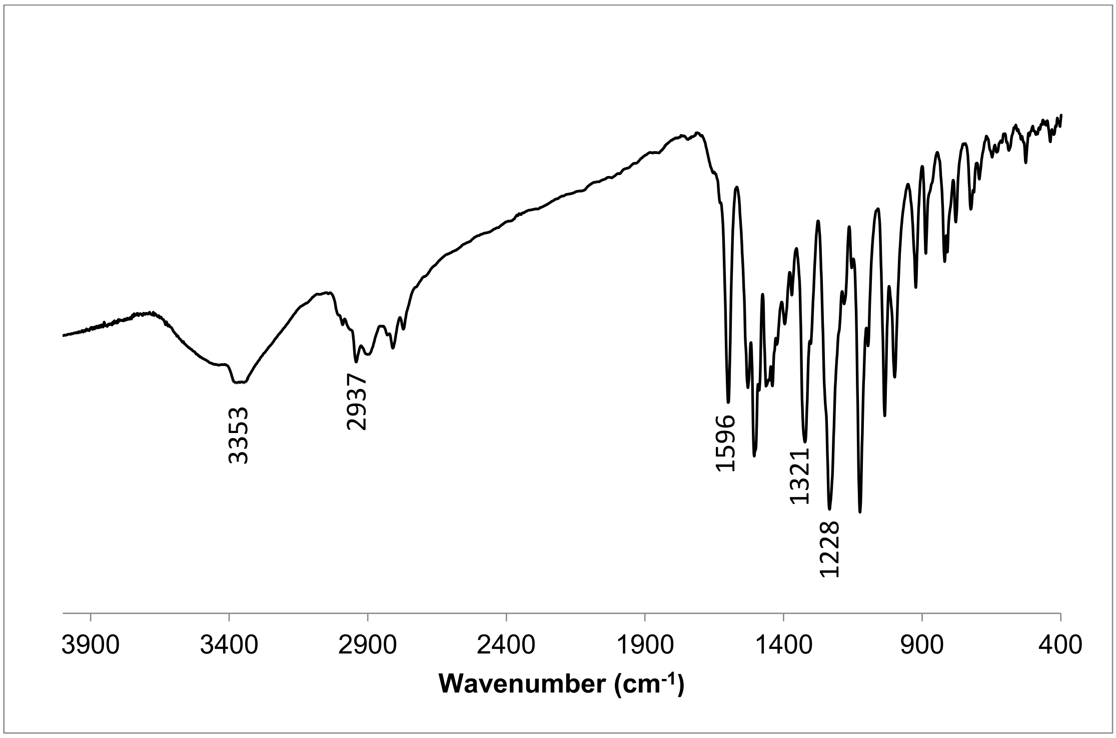

Figure S56. FT-IR spectrum of thiourea **5s**.

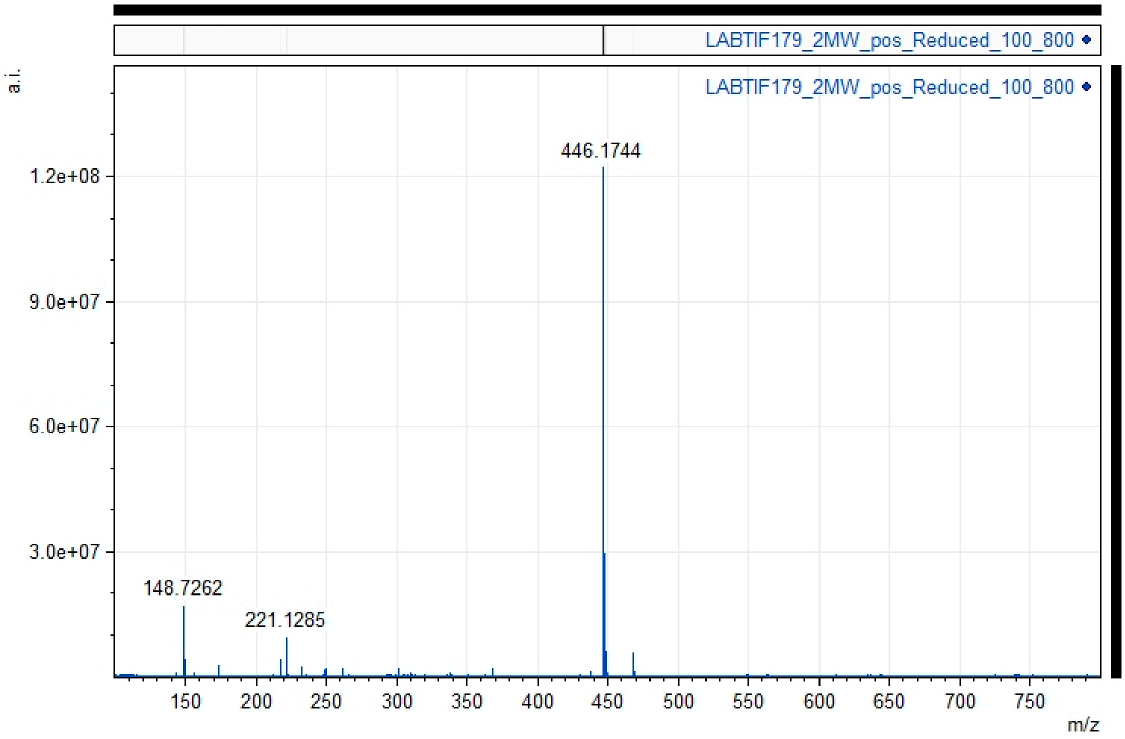

Figure S57. HR-MS spectrum of thiourea **5s**.

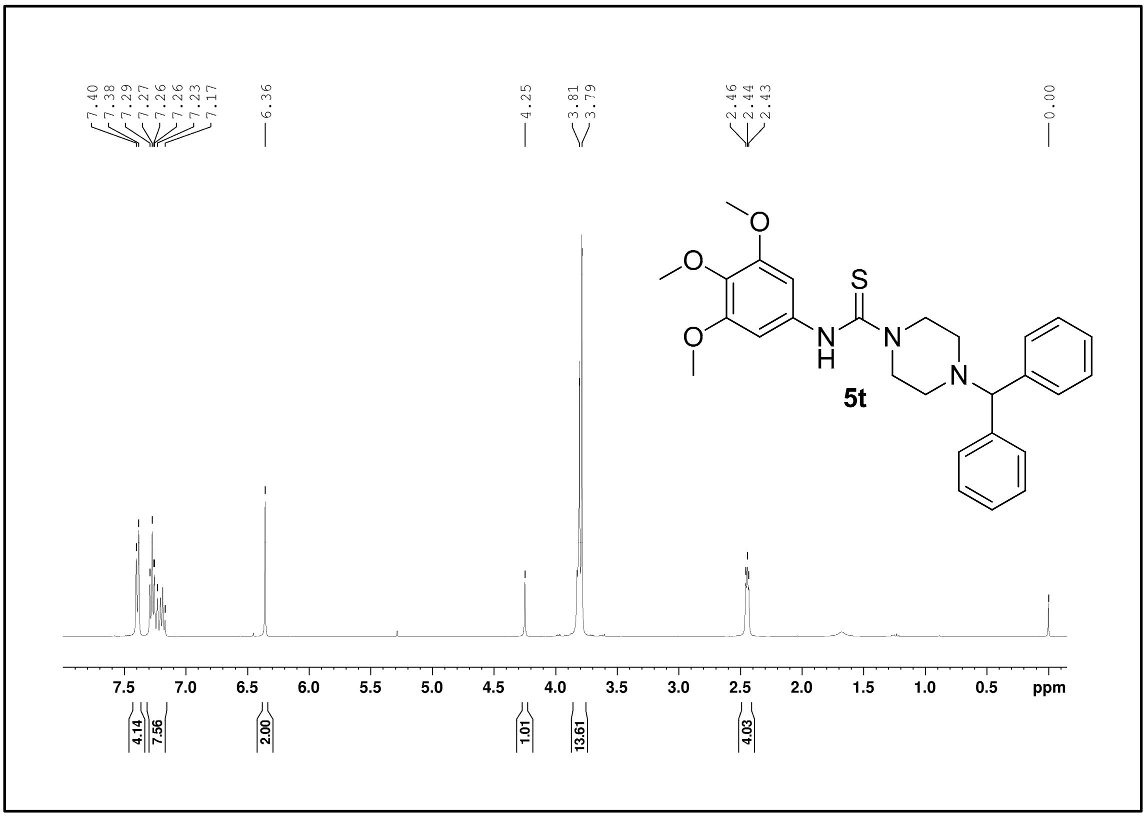

(A)

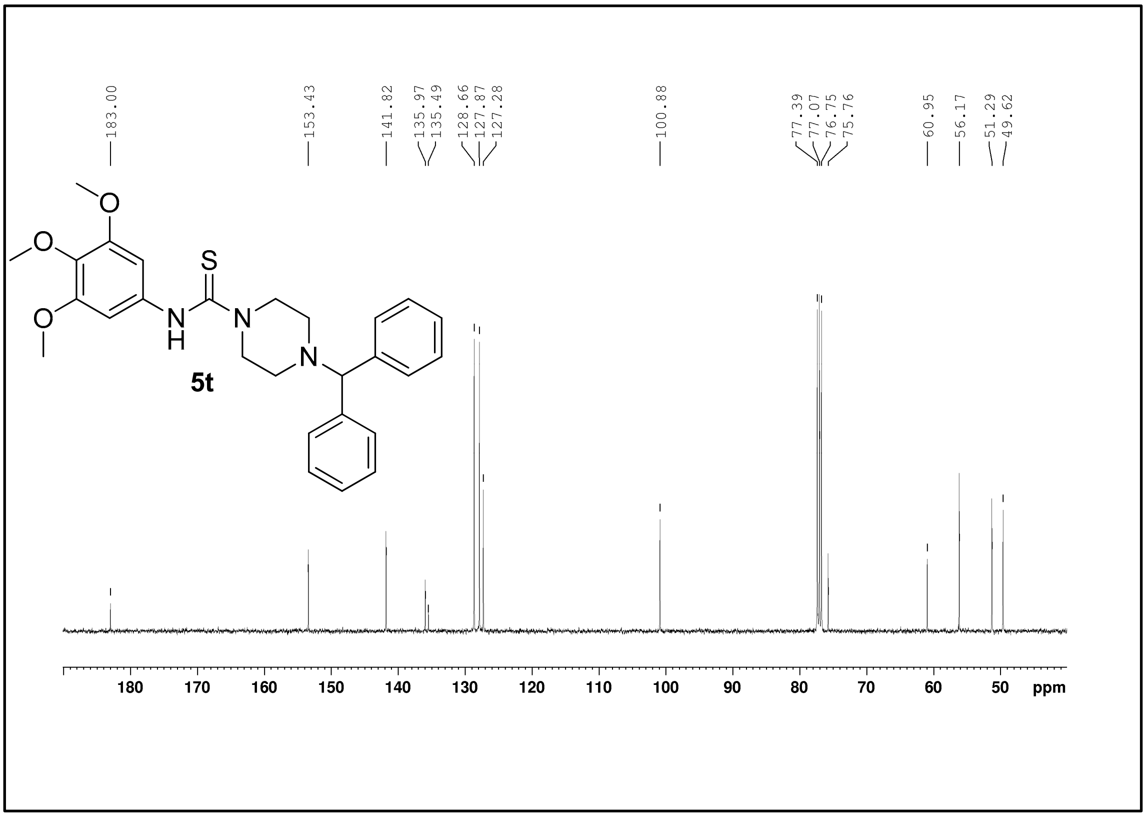

(B)

Figure S58. ^{1}H-NMR spectrum (A) and ^{13}C-NMR spectrum (B) of thiourea **5t**.

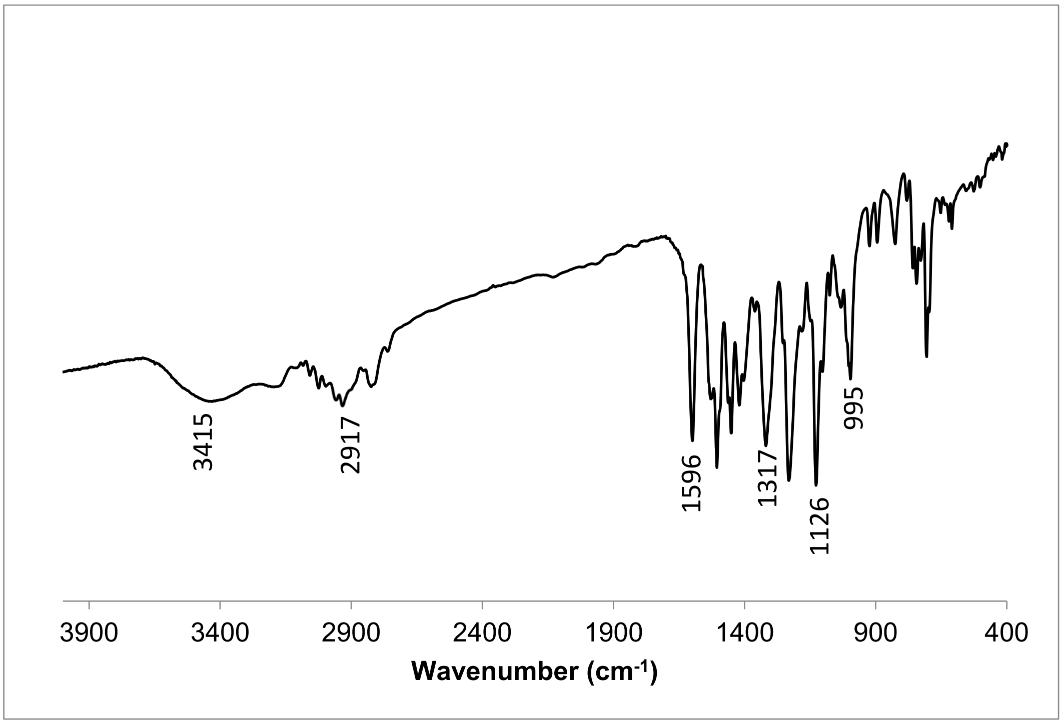

Figure S59. FT-IR spectrum of thiourea **5t**.

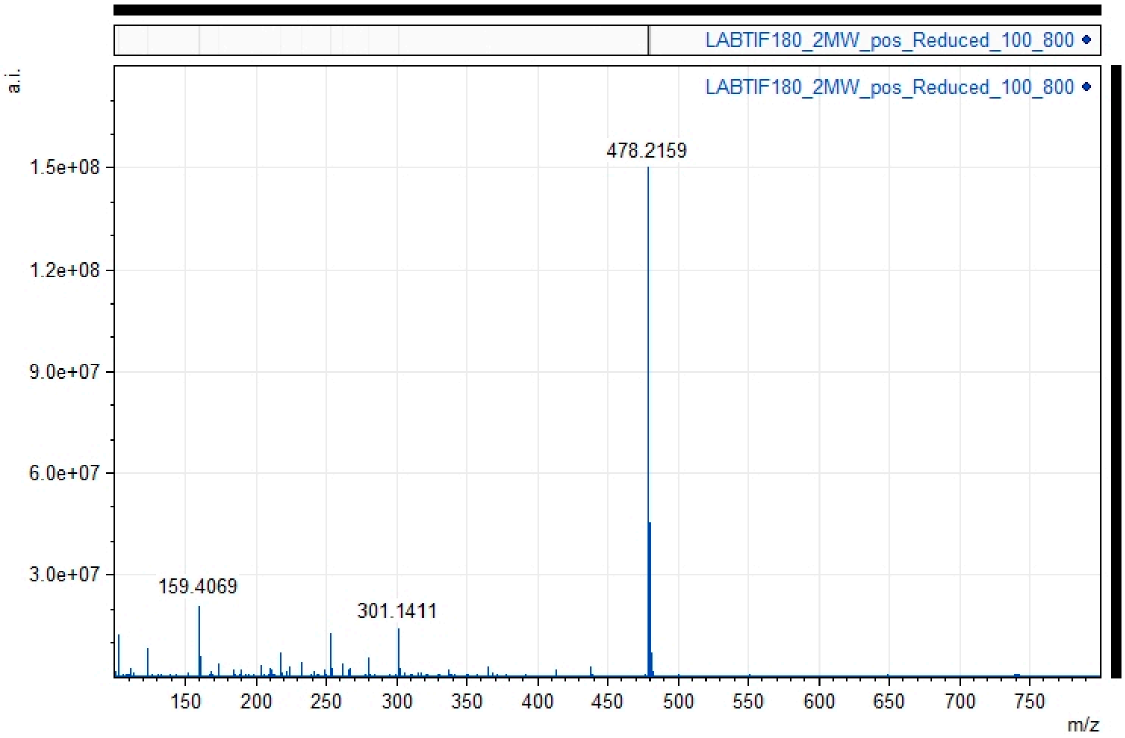

Figure S60. HR-MS spectrum of thiourea **5t**.

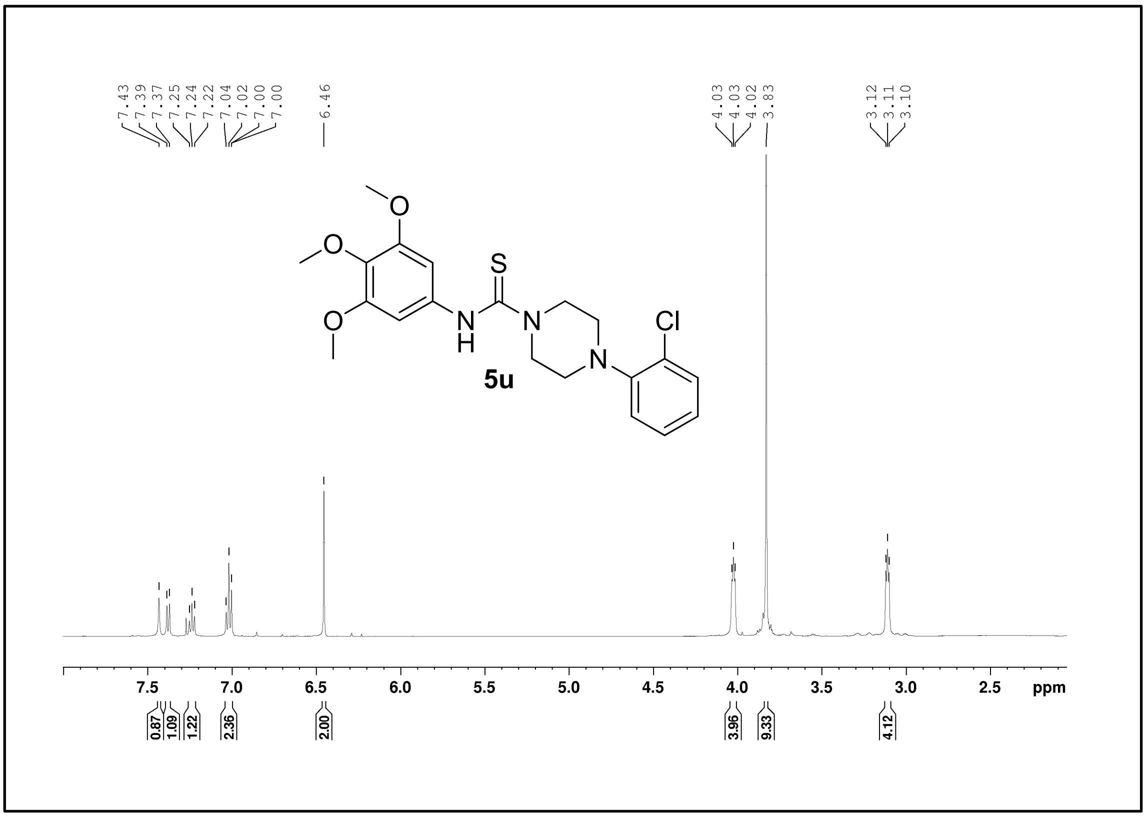

(A)

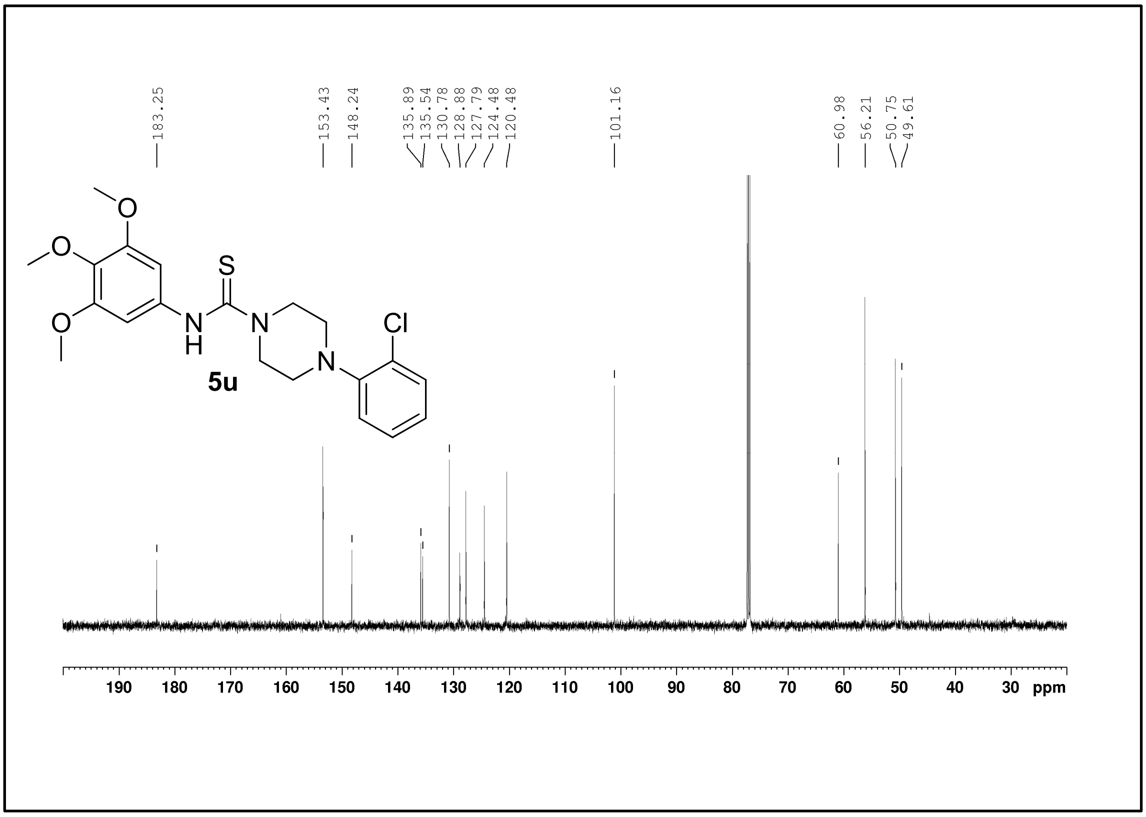

(B)

Figure S61. ^{1}H-NMR spectrum (A) and ^{13}C-NMR spectrum (B) of thiourea **5u**.

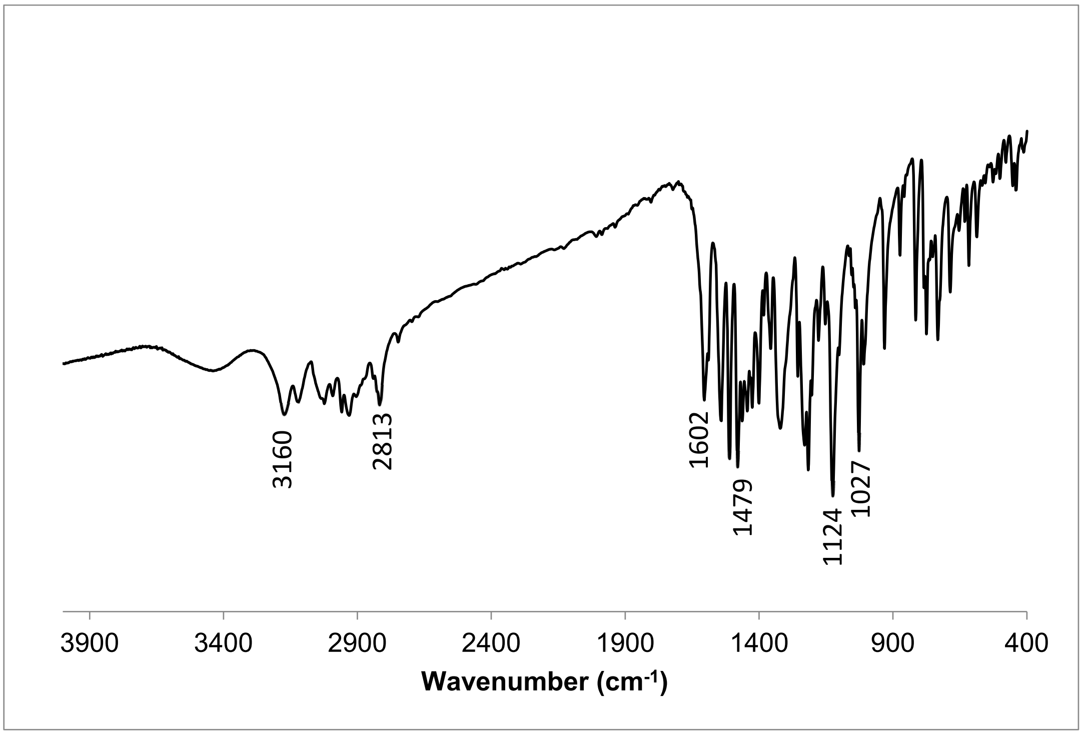

Figure S62. FT-IR spectrum of thiourea **5u**.

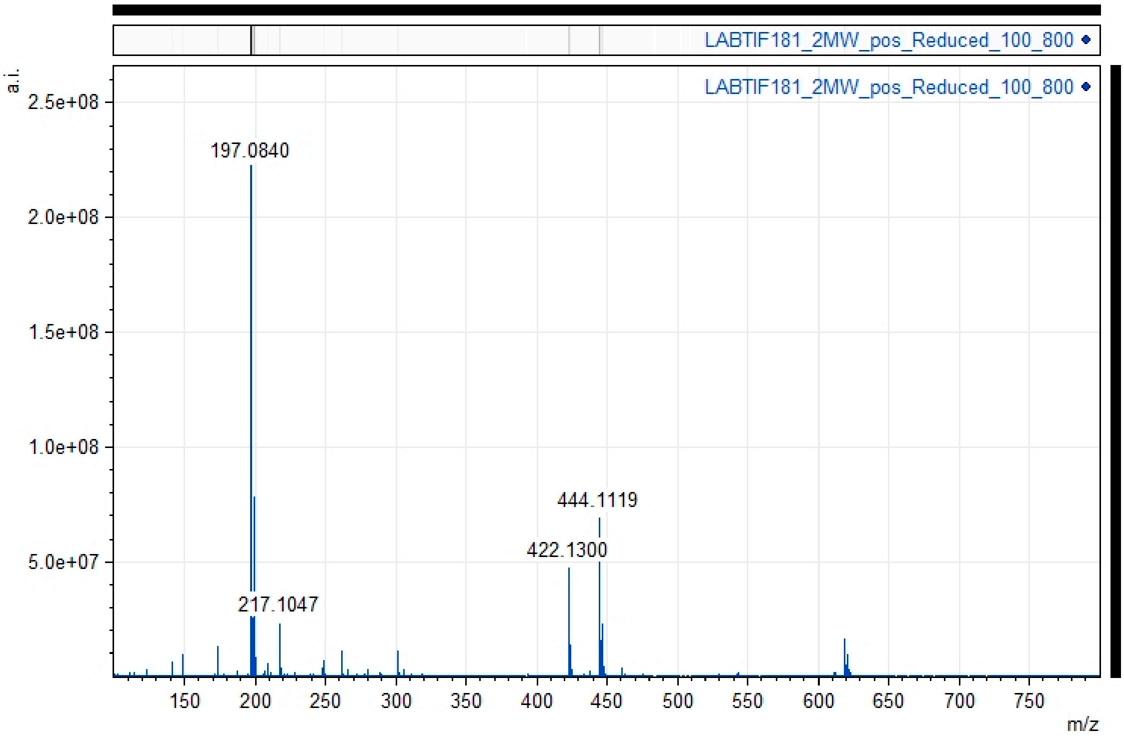

Figure S63. HR-MS spectrum of thiourea **5u**.

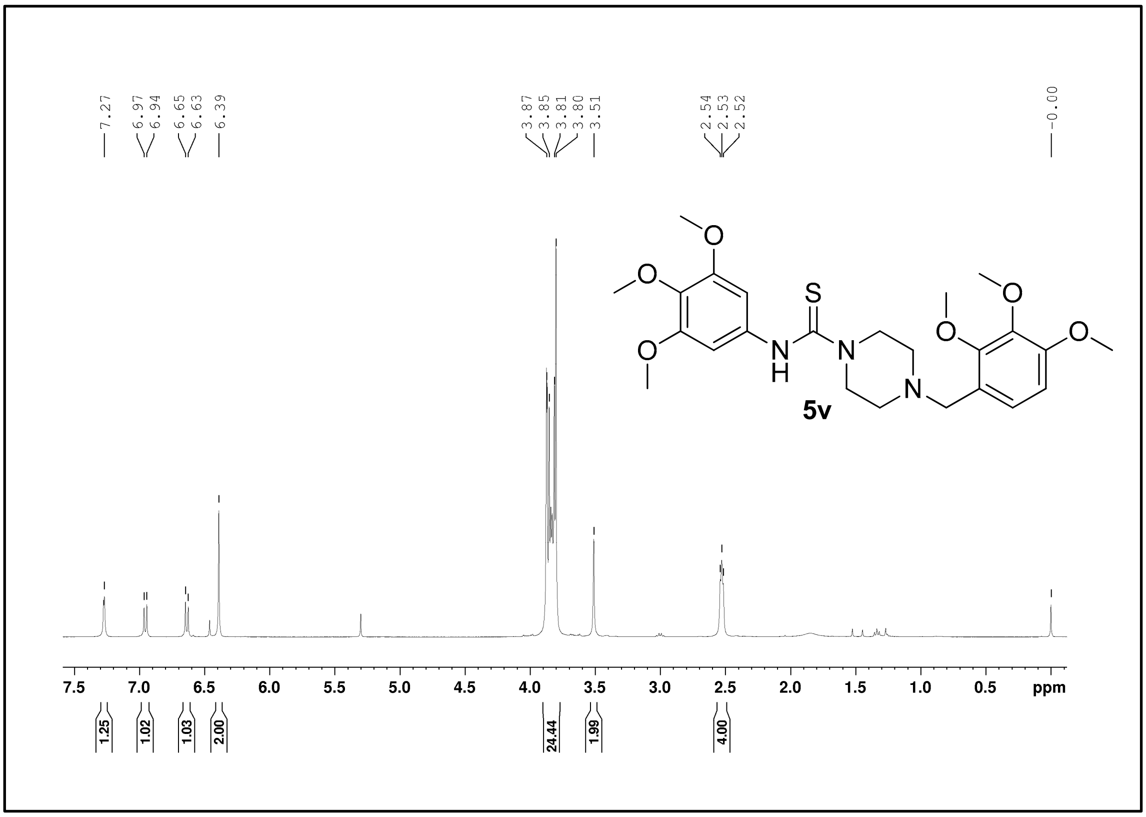

(A)

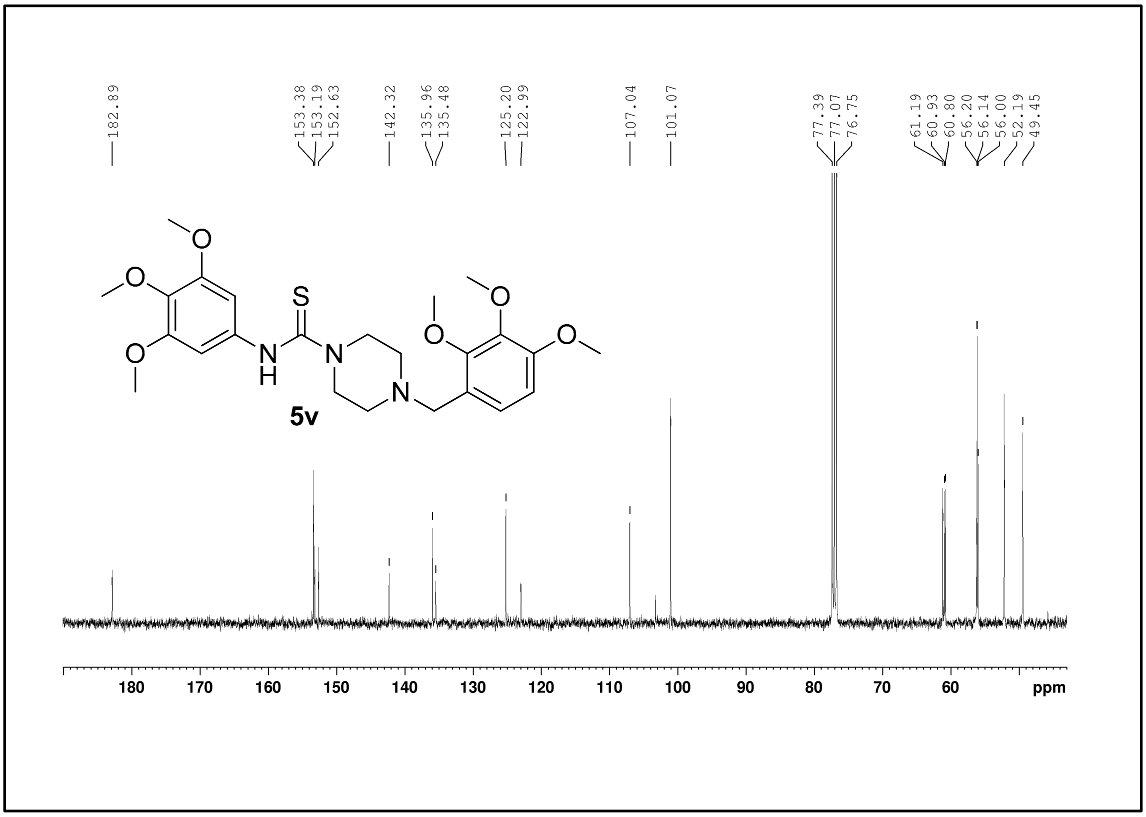

(B)

Figure S64. ^{1}H-NMR spectrum (A) and ^{13}C-NMR spectrum (B) of thiourea **5v**.

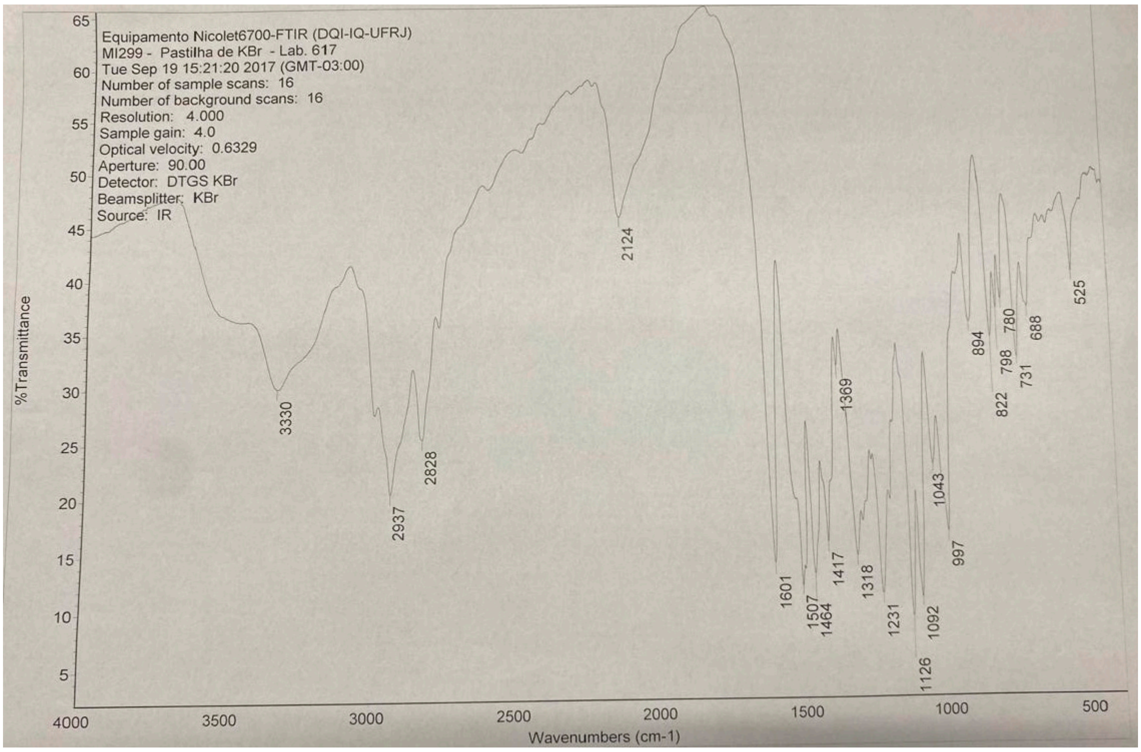

Figure S65. FT-IR spectrum of thiourea **5v**.

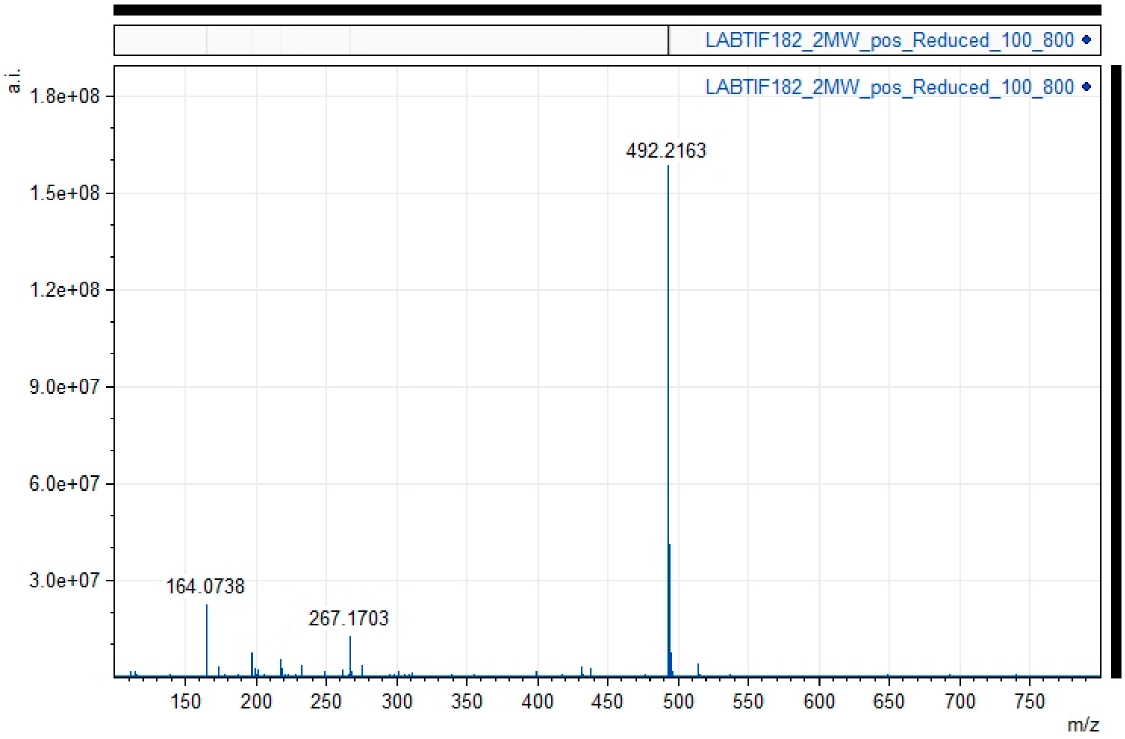

Figure S66. HR-MS spectrum of thiourea **5v**.

Table S1. Physiochemical parameters predicted by ACD/Percepta for the active thiourea compounds **3** against *L. amazonensis* promastigotes and their respective experimental IC_{50}.

**Compound**	**3d**[1]	**3e**[1]	**3h**	**3n**	**3o**[1]	**3p**[1]	**3q**	**3r**	**3s**	**3t**	**3u**	**3a'**
Isoelectric point	5.63	5.78	6.66	4.93	5.28	5	6.26	3.7	3.66	6.3	6.47	2
Molecular Weight	229.3	243.33	272.37	271.34	285.37	307.21	321.24	250.35	278.4	208.32	256.37	394.39
No. of Hydrogen Bond Donors	2	2	2	3	3	2	2	2	2	2	2	2
No. of Hydrogen Bond Acceptors	3	3	3	4	4	2	2	4	4	2	2	7
TPSA	69.04	69.04	65.38	85.25	85.25	56.15	56.15	110.17	110.17	56.15	56.15	137.86
No. of Rotatable Bonds	4	5	6	5	6	4	5	4	6	6	5	8
LogP	0.99	1.32	2.61	2.64	2.66	3.51	3.83	1.97	2.52	2.48	3.13	3.14
C Ratio	0.75	0.76	0.79	0.74	0.75	0.76	0.78	0.63	0.67	0.79	0.83	0.52
N Ratio	0.19	0.18	0.11	0.16	0.15	0.12	0.11	0.25	0.22	0.14	0.11	0.16
NO Ratio	0.19	0.18	0.16	0.21	0.2	0.12	0.11	0.25	0.22	0.14	0.11	0.28
Hetero Ratio	0.25	0.24	0.21	0.26	0.25	0.18	0.17	0.38	33	0.21	0.17	0.36
Number of Aromatic Rings	2	2	2	2	2	2	2	2	2	1	2	2
Log(BCF)	0.52	0.77	1.76	1.77	1.79	2.43	2.68	1.27	1.69	1.66	2.15	2.16
Log(Koc)	1.91	2.09	2.8	2.81	2.82	3.28	3.46	2.45	2.75	2.73	3.08	3.09
Parachor (cm^3)	501.08	546.75	609.27	589.15	634.81	557.43	603.1	516.03	600.65	499.81	590.24	705.6
Surface Tension (dyne/cm)	73.82	64.24	54.83	65.07	58.43	68.29	60.68	85.05	68.68	49.2	55.58	54.94
Polarizability	27.92	29.32	32.72	31.69	33.09	31.72	33.12	28.43	31.71	25.88	31.99	36.44
Molar Volume (cm^3)	170.95	193.13	223.9	207.43	229.61	193.91	216.09	169.93	208.65	188.72	216.18	259.17
Molar Refractivity	92.75	101.92	128.33	123.69	105.46	104.1	99.47	119.33	120.16	109.57	80.96	140.2
Average IC_{50} (μM)	77.5	90.2	6.9	7.9	30.3	28.2	30.7	42.5	30.7	18.8	56.2	22.3

[1]The compound also presented activity for amastigote form.

Table S2. Physiochemical parameters predicted by ACD/Percepta for the active thiourea compounds **5** against *L. amazonensis* promastigotes and their respective experimental IC_{50}.

**Compound**	**5b**	**5d**	**5e**	**5g**	**5h**[1]	**5i**[1]	**5k**	**5l**	**5n**	**5q**	**5t**[1]
Isoelectric point	8.42	9.18	10.84	8.8	10.45	10.29	10.2	10.03	10.63	10.75	8.97
Molecular Weight	333.45	355.46	369.48	387.54	401.57	415.59	345.89	359.92	415.55	365.5	477.62
Hydrogen Bond Donors	1	1	1	1	1	1	1	1	1	2	1
Hydrogen Bond Acceptors	5	5	5	3	3	3	3	3	6	5	6
TPSA	76.9	69.06	69.06	50.6	50.6	50.6	50.6	50.6	78.29	79.28	78.29
Rotatable Bonds	5	5	6	6	7	8	5	6	9	6	9
LogP	0.57	2.33	2.88	3.96	4.51	4.77	3.75	4.01	2.46	2.54	4.12
C Ratio	0.74	0.76	0.77	0.86	0.86	0.87	0.78	0.79	0.76	0.77	0.79
N Ratio	0.13	0.12	0.12	0.11	0.1	0.1	0.13	0.13	0.1	0.19	0.09
NO Ratio	0.22	0.2	0.19	0.11	0.1	0.1	0.13	0.13	0.21	0.19	0.18
Hetero Ratio	0.26	0.24	0.23	0.14	0.14	0.13	0.17	0.17	0.24	0.23	0.21
Number of Aromatic Rings	1	2	2	3	3	3	2	2	2	3	3
Log(BCF)	0.2	1.54	1.96	2.78	3.2	3.4	2.62	2.81	1.64	1.7	2.9
Log(Koc)	1.68	2.64	2.94	3.53	3.83	3.97	3.42	3.56	2.71	2.76	3.62
Parachor (cm^3)	736.46	763.64	803.71	887.76	927.83	967.91	753.09	793.16	931.87	817.84	1063.61
Surface Tension (dyne/cm)	60.31	69.46	61.76	61.37	55.88	54.73	57.61	56.07	51.7	69.19	55.78
Polarizability	36.77	40.4	41.81	47.64	49.04	50.87	39.43	41.27	47.31	43.44	55.58
Molar Volume (cm^3)	264.28	264.52	286.6	317.18	339.36	355.86	273.35	289.86	347.52	283.57	389.19
Molar Refractivity	92.75	101.92	105.46	120.16	123.69	128.33	99.47	104.1	119.33	109.57	140.2
IC_{50} (μM)	77.5	90.2	30.3	30.7	7.9	6.9	30.7	28.2	42.5	18.8	22.3

[1]The compound also presented activity for amastigote form.

Table S3. Pharmacokinetics and toxicological analysis of thiourea compounds with antileishmanial activity.

Thiourea	ADMET Risk	Absn Risk	CYP Risk	TOX Risk
**3d**[1]	2.668 (HEPX; 2C19; 2D6)	∅	1.668 (2C19; 2D6)	1.0 (HEPX)
**3e**[1]	0.249 (Xr; 2C19; 2D6)	∅	0.098 (2C19; 2D6)	0.151 (Xr)
**3h**	1.19 (fu; 2C9)	∅	1.0 (2C9)	∅
**3n**	1.138 (Xr; MUT)	∅	∅	1.138 (Xr; MUT)
**3o**[1]	1.4 (Xr; MUT)	∅	∅	1.4 (Xr; MUT)
**3p**[1]	2.94 (Kow; fu; 2C9-; 2D6-; CL)	0.545 (Kow)	1.805 (2C9-; 2D6-; CL)	∅
**3q**	2.136 (Kow; fu; 3A4; CL)	0.515 (Kow)	0.947 (3A4; CL)	∅
**3r**	1.622 (Xr; Xm)	∅	∅	1.622 (Xr; Xm)
**3s**	2.341 (Xr; Xm; 2D6)	∅	0.415 (2D6)	1.927 (Xr; Xm)
**3t**	1.184 (2C19; 2D6)	∅	1.184 (2C19; 2D6)	∅
**3u**	0.273 (Xr; 2D6)	∅	0.015 (2D6)	0.258 (Xr)
**3a'**	4.143 (fu; rat; Xr; Xm; 2C9; 2C19)	∅	1.845 (2C9; 2C19)	2.059 (rat; Xr; Xm)
**5b**	2.889 (rat; Xr; 2D6; 3A4)	∅	2.0 (2D6; 3A4)	0.889 (rat; Xr)
**5d**	1.471 (rat; 2C19; 2D6; 3A4)	∅	1.165 (2C19; 2D6; 3A4)	0.305 (rat)
**5e**	1.577 (rat; Xm; 2D6; 3A4)	∅	1.259 (2D6; 3A4)	0.318 (rat; Xm)
**5g**	4.987 (Kow; Sw; fu; hERG; Xm; 2D6; 3A4; CL)	1.151 (Kow; Sw)	2.216 (2D6; 3A4; CL)	0.62 (hERG; Xm)
**5h**[1]	6.096 (Kow; Sw; fu; hERG; Xm; 2D6; 3A4; CL)	1.18 (Kow; Sw)	3.0 (2D6; 3A4; CL)	1.18 (hERG; Xm)
**5i**[1]	6.701 (Kow; Sw; fu; hERG; Xm; 2D6; 3A4; CL)	1.602 (Kow; Sw)	3.0 (2D6; 3A4; CL)	1.163 (hERG; Xm)
**5k**	2.945 (Sw; fu; hERG; Xm; 2D6; 3A4; CL)	0.318 (Sw)	2.041 (2D6; 3A4; CL)	0.14 (hERG; Xm)
**5l**	4.446 (Kow; Sw; fu; hERG; 2D6; 3A4; CL)	0.853 (Kow; Sw)	2.616 (2D6; 3A4; CL)	0.266 (hERG)
**5n**	1.983 (RotB; 3A4; CL)	0.5 (RotB)	1.483 (3A4; CL)	∅
**5q**	3.005 (Sw; Xr; 2D6; 3A4)	0.222 (Sw)	1.783 (2D6; 3A4)	1.0 (Xr)
**5t**[1]	5.691 (Size; RotB; Kow; fu; rat; Xm-; 3A4; CL)	1.929 (Size; RotB; Kow)	2.0 (3A4; CL)	0.762 (rat; Xm-)
Cut-off[2]	≤7	≤4	≤2	≤2

The following codes correspond to potential pharmacokinetics and toxicological problems a compound might have: Size = molecule size; RotB=rotatable bonds; Kow=lipophilicity; Sw=water solubility; fu=fraction unbound; Vd=volume of distribution; hERG= Ether-à-go-go-Related Gene (hERG) Channel Inhibition; rat=acute rat toxicity; Xr=carcinogenicity in rat; Xm=carcinogenicity in mice; HEPX=hepatotoxicity; MUT=likely Ames positive; 2C19=high clearance by CYP 2C19; 2D6=high clearance by CYP 2D6; 2C9=high clearance by CYP 2C9; 3A4=high clearance by CYP 3A4; CL=high microsomal clearance; S_97: mutagenic in S. typhimurium TA97 + TA1537 strains without microsomal activation; m_97: mutagenic in S. typhimurium TA97 + TA1537 strains with microsomal activation; m_535: mutagenic in S. typhimurium TA1535 strains with microsomal activation; NIHS: classified as mutagenic by a model trained on the NIHS's Ames test dataset.[1] The compound also presented activity for amastigote forms. [2] Cut-off based on an empirical distribution calculated from commercial compounds available in the World Drug Index subset.

References

[1] Taha, M.; Ismail, N.H.; Jamil, W.; Khan, K.M.; Salar, U.; Kashif, S.M.; Rahim, F.; Latif, Y. Synthesis and Evaluation of Unsymmetrical Heterocyclic Thioureas as Potent β-Glucuronidase Inhibitors. *Med Chem Res* **2015**, *24*, 3166–3173, doi:10.1007/s00044-015-1369-x.

[2] Khachikyan, R.Dzh.; Ovakimyan, Z.G.; Panosyan, G.A.; Tamazyan, R.A.; Ayvazyan, A.G. Features of Reactions of (E)-1-(β-Aroylvinyl)Pyridinium Bromides with Binucleophiles. *Russ J Gen Chem* **2016**, *86*, 1574–1580, doi:10.1134/S1070363216070070.

[3] Wu, H.; Sun, Y.-F.; Zhang, C.; Miao, C.-B.; Yang, H.-T. A Facile Method for the Preparation of Carbodiimides from Thioureas and (Boc)2O. *Tetrahedron Letters* **2018**, *59*, 739–742, doi:10.1016/j.tetlet.2018.01.025.

[4] Keesara, S.; Parvathaneni, S.; Mandapati, M.R. *N,N′*-Mono Substituted Acyclic Thioureas: Efficient Ligands for the Palladium Catalyzed Heck Reaction of Deactivated Aryl Bromides. *Tetrahedron Letters* **2014**, *55*, 6769–6772, doi:10.1016/j.tetlet.2014.09.053.

[5] Pingaew, R.; Prachayasittikul, V.; Anuwongcharoen, N.; Prachayasittikul, S.; Ruchirawat, S.; Prachayasittikul, V. Synthesis and Molecular Docking of N,N′-Disubstituted Thiourea Derivatives as Novel Aromatase Inhibitors. *Bioorganic Chemistry* **2018**, *79*, 171–178, doi:10.1016/j.bioorg.2018.05.002.

[6] Gan, S.-F.; Wan, J.-P.; Pan, Y.-J.; Sun, C.-R. Highly Efficient and Catalyst-Free Synthesis of Substituted Thioureas in Water. *Mol Divers* **2011**, *15*, 809–815, doi:10.1007/s11030-010-9298-6.

[7] Begum, S.; Choudhary, M.I.; Khan, K.M. Synthesis, Phytotoxic, Cytotoxic, Acetylcholinesterase and Butrylcholinesterase Activities of N,N′-Diaryl Unsymmetrically Substituted Thioureas. *Natural Product Research* **2009**, *23*, 1719–1730, doi:10.1080/14786410802223778.

[8] Kapanda, C.N.; Masquelier, J.; Labar, G.; Muccioli, G.G.; Poupaert, J.H.; Lambert, D.M. Synthesis and Pharmacological Evaluation of 2,4-Dinitroaryldithiocarbamate Derivatives as Novel Monoacylglycerol Lipase Inhibitors. *J. Med. Chem.* **2012**, *55*, 5774–5783, doi:10.1021/jm3006004.
